# Spatiotemporal Dynamics and Human Health Risk Assessment of Potentially Toxic Elements in Global Urban Soils: A Systematic Meta-Analysis

**DOI:** 10.3390/toxics14060496

**Published:** 2026-06-07

**Authors:** Jiaxuan Cui, Jilong Lu, Yawen Lai, Qiaoqiao Wei, Xinyun Zhao

**Affiliations:** College of Geo-Exploration Science and Technology, Jilin University, Changchun 130026, China; jxcui23@mails.jlu.edu.cn (J.C.); laiyw@jlu.edu.cn (Y.L.); qiaoqiao@jlu.edu.cn (Q.W.); zhaoxy15@jlu.edu.cn (X.Z.)

**Keywords:** potentially toxic elements (PTEs), geo-accumulation index, Nemerow integrated index, pediatric exposure, urban functional zones, risk-based soil management

## Abstract

Urban soil contamination by potentially toxic elements (PTEs) is a recognized health concern in densely populated urban environments. Through a systematic meta-analysis of 91 peer-reviewed studies (2000–2025) reporting 12,174 sampling sites in capital and core cities, we characterized regional patterns in the spatiotemporal dynamics and health risks of eight PTEs across two well-represented continental subsets (Asia, k = 18–36 per element; Europe, k = 11–23 per element) with comparative reference to the Americas, Africa, and Oceania. Given the uneven geographic distribution of qualifying primary studies, continental comparisons should be interpreted as hypothesis-generating: Asia (k = 18–36 per element) and Europe (k = 11–23 per element) provide the statistically robust core of the synthesis, while results for the Americas (k = 3–7 for several elements), Africa (k = 4–15), and Oceania (k = 2) are presented as illustrative rather than statistically representative. Pooled concentrations followed Zn (138.59) > Pb (56.97) > Cr (54.26) > Cu (47.00) > Ni (31.94) > As (8.56) > Hg (3.13) > Cd (1.23) mg·kg^−1^. Within the well-represented Asian and European subsets, Asian cities showed the most severe enrichment of As, Cd, Cr, and Hg (*I_geo_* > 4 in hotspots such as Kathmandu *I_geo_* (Cd) = 7.06 and Jinan *I_geo_* (Hg) = 5.27), whereas European centres exhibited substantial legacy Pb accumulation (pooled mean 87.69 mg·kg^−1^). A reproducible pollution gradient was identified across functional zones: industrial > transportation ≥ residential > commercial > agricultural > urban green areas. The deterministic non-carcinogenic Hazard Index (*HI* = 1.49) for children in Asia exceeded the safe threshold (*HI* > 1), driven primarily by As and Cr exposure via incidental soil-and-dust ingestion. Monte Carlo probabilistic assessment (N = 10,000) confirmed elevated cumulative non-carcinogenic risk at the median of the exposure distribution for children in the data-rich Asian (P50 = 1.55; P(*HI* > 1) = 81.9%) and European (P50 = 1.28; P(*HI* > 1) = 69.8%) subsets, with adults in both subsets remaining well below the safety threshold (P(*HI* > 1) = 0.0%). Temporal analysis revealed a decoupling between economic growth and PTE accumulation in long-established cities, together with an inverse Ni–population correlation indicative of strategic resource allocation. For Asian capital and core cities, where the evidence base is strongest (k = 18–36 per element), the present synthesis supports further investigation of risk-based, child-centric soil management as a public-health priority. For European cities (k = 11–23 per element), the same direction of risk is indicated but should be confirmed in regionally focused syntheses. Policy considerations for under-represented regions should await expansion of the primary monitoring base.

## 1. Introduction

Rapid global urbanization has transformed soil ecosystems into primary sinks for potentially toxic elements (PTEs). Unlike organic pollutants, PTEs are persistent, non-biodegradable, and bioaccumulative [1,2]. Once introduced into urban soils through industrial emissions, traffic exhaust, and waste disposal, they can alter soil physicochemical properties and enter the human body via ingestion, dermal contact, and inhalation, causing acute or chronic health issues [3]. Therefore, understanding the distribution and toxicological risks of PTEs in urban environments is a critical global priority.

A growing body of literature has examined PTEs in urban soils over the past two decades, yet findings remain regionally fragmented [4,5,6]. In Europe, studies emphasize the legacy of historical industrialization and leaded gasoline, with Pb hotspots persisting decades after policy intervention [7,8]. North American research has documented Pb–Zn co-enrichment in old industrial cities such as New York and Mexico City, attributed to pre-1980s emissions and ongoing traffic loads. Russian work links PTE patterns to Soviet-era industrial planning and natural background variability [9]. In rapidly developing Asian and African cities, recent studies report severe but heterogeneous enrichment of Cd, As, Cr, and Hg driven by coal combustion, artisanal mining, and uncontrolled urban expansion [10,11,12]. Although these regional studies provide valuable insights, their differing methodologies, exposure parameters, and reference values impede direct comparison and obscure the global picture.

Four critical gaps therefore remain in the global PTE literature. (1) Methodological inconsistency: most syntheses pool raw concentration data without harmonizing analytical methods, detection-limit treatments, or background values, introducing systematic bias. (2) Functional-zone resolution: few studies have quantitatively contrasted PTE patterns across all major urban land-use types on a global scale. (3) Demographically stratified risk: cross-continental health risk assessments separating children from adults are rare, despite the disproportionate vulnerability of pediatric populations. (4) Conceptual conflation: the literature frequently uses ‘contamination’ (single-element enrichment) and ‘pollution’ (integrated multi-element degradation) interchangeably, leading to over- or under-estimation of risk [13,14].

To address these gaps, the present study tests four hypotheses by means of a systematic meta-analysis of eight priority PTEs (As, Cr, Cu, Ni, Pb, Zn, Cd, Hg) in surface soils of 91 core and capital cities, with the database weighted toward Asian (k = 18–36 per element) and European (k = 11–23 per element) urban centres and providing more limited coverage of the Americas, Africa, and Oceania:

(H1) Intercontinental disparities in PTE enrichment between the well-represented Asian and European subsets reflect differences in industrialization stage and regulatory history, after normalizing for local geochemical background via the *I_geo_* metric;

(H2) Urban functional zones impose a reproducible pollution gradient detectable across continents;

(H3) Children in the well-represented Asian and European subsets face cumulative *HI* > 1 at the median of the exposure distribution, with rapidly industrializing regions exhibiting the highest exceedance probability;

(H4) Within the data-rich subsets, population size and economic growth show element-specific (rather than uniform) coupling with soil PTE accumulation.

The work delivers a standardized global database, separately quantified single-element (*I_geo_*) and integrated (*P_N_*) indices, age- and continent-stratified US-EPA risk estimates, and an attribution analysis of socioeconomic drivers.

We emphasize at the outset that this synthesis is constrained by the geographic distribution of primary studies meeting our pre-specified inclusion criteria. The number of qualifying primary studies (k) is markedly uneven across continental subsets: Asia (k = 18–36 per element) and Europe (k = 11–23 per element) provide the statistically robust core of the synthesis, while the Americas (k = 3–7 for several elements), Africa (k = 4–15), and Oceania (k = 2 for all reported elements) provide considerably smaller evidence bases.

This uneven distribution is a direct and intended consequence of the study design rather than a sampling oversight. Our inclusion criteria (Section 2.2) deliberately privilege study quality and representativeness over study volume: we require simultaneous measurement of ≥3 priority PTEs, ≥10 sampling sites per functional zone, presence of locally determined geochemical background, explicit QC/QA documentation, and availability of paired socioeconomic covariates. Imposing these criteria on the global literature returns a relatively small but methodologically homogeneous corpus, which is essential for a quantitative meta-analysis that pools effect sizes across studies; relaxing these criteria to enlarge k would introduce uncontrolled methodological heterogeneity and risk overfitting at the inter-study level.

The resulting database is heavily weighted toward Asian and European cities, with comparatively limited representation from Oceania, sub-Saharan Africa, and Latin America. Accordingly, this work does not aim to establish universal global patterns of urban soil PTE behaviour. Rather, it provides (1) a quantitatively defensible synthesis for the well-represented Asian and European subsets and (2) hypothesis-generating comparisons for under-represented regions that we explicitly flag as exploratory throughout. Conclusions and policy recommendations are scaled to the evidence base of the corresponding region. For continental subsets with small evidence bases (Oceania, k = 2; Americas, k = 3–7 for several elements; Africa Hg, k = 4), pooled estimates are reported with wide 95% confidence intervals and are treated as illustrative rather than as the basis for continental rankings or policy recommendations.

## 2. Materials and Methods

### 2.1. Study Design and Meta-Analysis Workflow

This study uses the PRISMA (Preferred Reporting Items for Systematic Reviews and Meta-Analyses) guidelines in order to ensure a rigorous and transparent meta-analysis. We did a systematic search published in peer-reviewed journals, using Web of Science, ScienceDirect, and CNKI, as well as other publicly available academic platforms. This helped find studies on PTEs in urban soils published between 2000 and 2025. To minimize methodological bias, the quality of each included study was evaluated using the Newcastle–Ottawa Scale (NOS) [15].

The synthesis presented here meets the established methodological criteria for a quantitative meta-analysis in environmental geochemistry: (1) a transparent, PRISMA-compliant search and screening protocol with pre-specified inclusion criteria (Section 2.2); (2) quantitative pooling of effect sizes under a DerSimonian–Laird random-effects model with reporting of pooled means, 95% confidence intervals, and the heterogeneity index *I*^2^; (3) formal evaluation of publication bias via regression-based effect-size analysis, complemented by stratified sensitivity analyses across analytical techniques and sampling decades; and (4) propagation of parameter uncertainty into the downstream risk assessment through 10,000-iteration Monte Carlo simulation (Section 3.6.3). The synthesized dataset comprises 12,174 sampling sites from 91 independent primary studies. To prevent natural lithogenic variability from confounding the continental comparisons, all city-level concentrations are normalized to their locally determined geochemical backgrounds via the *I_geo_* metric prior to aggregation; the continental groupings therefore reflect socio-economic strata—industrialization stage and regulatory history—rather than geochemical units (Section 3.1).

### 2.2. Literature Search and Data Acquisition

**Figure 1:**
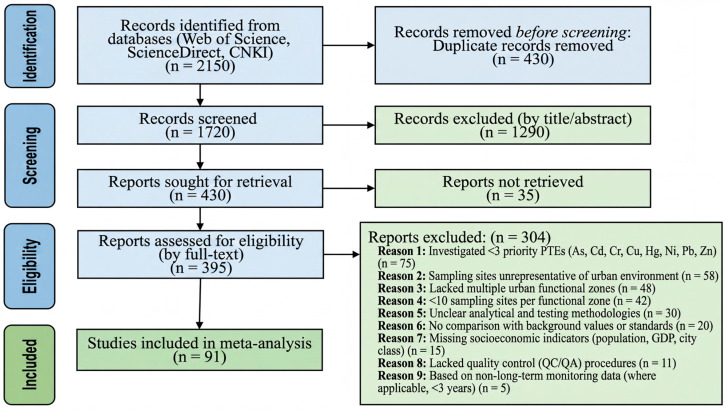
PRISMA flow diagram for the systematic literature search and study selection process. Blue-shaded boxes indicate the main PRISMA pathway through identification, screening, and eligibility; green-shaded boxes indicate decision outcomes, including exclusions, non-retrieval, and final inclusion.

The literature screening process was based on specific inclusion criteria: (1) simultaneous investigation of at least 3 priority PTEs (As, Cr, Cu, Ni, Pb, Zn, Cd, Hg); (2) high representativeness of sampling sites for the target city’s environment; (3) coverage of diverse functional zones (e.g., residential, industrial, and green spaces); (4) a minimum of 10 sampling sites per functional zone; (5) detailed description of analytical methodologies; (6) availability of local/regional geochemical background values or standards; (7) inclusion of socioeconomic indicators such as GDP and population; (8) explicit reporting of quality control (QC/QA) procedures; and (9) preference for long-term monitoring data (≥3 years) where applicable. Following these criteria, 91 high-quality peer-reviewed studies were finally selected for the global meta-analysis [16,17,18,19,20,21,22,23,24,25,26,27,28,29,30,31,32,33,34,35,36,37,38,39,40,41,42,43,44,45,46,47,48,49,50,51,52,53,54,55,56,57,58,59,60,61,62,63,64,65,66,67,68,69,70,71,72,73,74,75,76,77,78,79,80,81,82,83,84,85,86,87,88,89,90,91,92,93,94,95,96,97,98,99,100,101,102,103,104,105]. The detailed study selection process is illustrated in Figure 1. Detailed information and specific data points from each study are provided in Table S1 of the Supplementary Materials.

As shown in Figure 1, the PRISMA workflow comprises four sequential stages. The Identification phase yielded 2150 records from Web of Science, ScienceDirect, and CNKI; 430 duplicates were removed prior to screening. In the Screening phase, 1290 records were excluded based on title and abstract review (off-topic, non-empirical, or non-urban). Of the 430 reports sought for retrieval, 35 were not retrievable due to access limitations. The Eligibility phase applied the nine quantitative inclusion criteria of Section 2.2 to the remaining 395 full-text reports, excluding 304 studies. The most common exclusion reasons were investigation of fewer than three priority PTEs (*n* = 75) and an unrepresentative sampling design (*n* = 58). The final 91 studies, covering 12,174 sampling sites, form the basis of all subsequent analyses.

#### Data Harmonization and Background-Value Selection

To minimize methodological bias arising from inter-study heterogeneity, all extracted concentrations were harmonized as follows.

(1) Unit conversion: All values were standardized to mg·kg^−1^ (dry weight); studies reporting wet weight or μg·g^−1^ were converted using the original moisture content or unit factor.
(2) Below-detection-limit values were replaced by half the reported detection limit.
(3) Analytical method: Raw concentration data from diverse instruments (e.g., ICP-MS, ICP-OES, AAS, and XRF) were retained without arbitrary mathematical conversion, as standardizing across different extraction protocols (e.g., total vs. pseudo-total digestion) is unfeasible retrospectively. Instead, the analytical method was recorded for each study, and its influence on pooled estimates was evaluated via stratified sensitivity analysis (Figure S45).
(4) Background values: A hierarchical scheme was used—local soil background (preferred) > national geochemical baseline (e.g., CGS for China; FOREGS for Europe) > continental average > global crustal average.

### 2.3. Pollution and Contamination Assessment

To address the terminological gap between “contamination” and “pollution,” two quantitative indices were employed. It must be emphasized that all indices in this study were recalculated from the raw measured concentrations of each primary study, using a uniform set of exposure parameters, *RfD*s, and *SF*s. Index values reported in the original studies were not used in pooling; instead, they were recalculated.

#### 2.3.1. Calculation and Classification of the Geo-Accumulation Index (*I_geo_*)

The enrichment of individual PTEs relative to geochemical background levels was assessed using the *I_geo_* [106]:

(1) $$I_{geo}\;=\;\log_{2}\left(\frac{C_{i}}{1.5\;\times \;B_{i}}\right)$$

where *C_i_* represents the measured concentration of element *i* (mg·kg^−1^), and *B_i_* is its corresponding background value. The constant 1.5 accounts for potential lithological variations [107].

#### 2.3.2. Calculation and Classification of the Nemerow Integrated Pollution Index (*P_N_*)

While *I_geo_* assesses the contamination of single elements, the Nemerow Integrated Pollution Index (*P_N_*) was employed to evaluate the combined pollution intensity of all targeted PTEs at the site level. It is a multi-factor evaluation method for comprehensive pollutants, capable of comprehensively reflecting the pollution levels of different PTEs in the soil and calculating the pollution index of individual PTEs [108]:

(2) $$P_{i}\;=\;\frac{C_{i}}{S_{i}}$$

(3) $$P_{N}=\sqrt{\frac{\left(P_{i,\;\max}^{2}+P_{i,\;\mathrm{ave}}^{2}\right)}{2}}$$

The *P_ij_* was used to quantify the degree of PTE pollution in different functional zones:

(4) $$P_{ij\;}=\;\sqrt{\frac{{\left(\frac{\mathrm{Max}C_{i}}{S_{ij}}\right)}^{2}+{\left(\frac{1}{n}\sum \frac{C_{i}}{S_{ij}}\right)}^{2}}{2}}$$

where *i* is the PTE species, *C_i_* is the actual measured value of *i* PTE (mg·kg^−1^). *j* is the number of urban functional zones (1—residential, 2—traffic, 3—industrial, 4—commercial, 5—agricultural, 6—urban green area). *S_i_* and *S_ij_* are the background values of the soil environment, and the values are selected as indicated in the comment of Formula (1). *n* is the total number of PTE species.

### 2.4. Human Health Risk Assessment (HHRA) Methodology

Health risks were quantified using the US EPA Health Risk Assessment model [109]. The Average Daily Dose (*ADD*, mg kg^−1^ day^−1^) for three exposure pathways (ingestion, dermal contact, and inhalation) was calculated as follows:

(5) $${ADD}_{\mathrm{inh}}=\frac{C\times {IR}_{\mathrm{inh}}\times EF\times ED}{PEF\times BW\times AT}$$

(6) $${ADD}_{\mathrm{der}}=\frac{C\times SA\times AF\times ABF\times EF\times ED}{BW\times AT}\times 10^{-6}$$

(7) $${ADD}_{\mathrm{ing}}=\frac{C\times {IR}_{\mathrm{ing}}\times EF\times ED}{BW\times AT}\times 10^{-6}$$

where parameters such as body weight (*BW*) and exposure frequency (*EF*) were stratified by continent and age groups (children and adults) based on the Exposure Factors Handbook of the Chinese Population [110] and the US EPA supplemental guidance. Detailed exposure parameters are summarized in Table 1.

Non-carcinogenic risks were evaluated via the Hazard Quotient (*HQ*) and Hazard Index (*HI*):

(8) $$HQ\;=\;\frac{ADD}{RfD}$$

(9) $$\begin{array}{c} HI=\sum \frac{ADD}{RfD}=\sum \left(HQ_{\mathrm{inh}}+HQ_{\mathrm{der}}+HQ_{\mathrm{ing}}\right) \end{array}$$

Carcinogenic risks (*CR)* were determined using pathway-specific slope factors (*SF*):

(10) $$\begin{array}{c} CR=\sum ADD\times SF=\sum \left({CR}_{\mathrm{inh}}+{\;CR}_{\mathrm{der}}{+\;CR}_{\mathrm{ing}}\right) \end{array}$$

The toxicological parameters (*RfD* and *SF*) used in this study are listed in Table A1. It is well documented that published soil PTE risk assessments differ in their toxicological reference values, with some authors citing the USEPA IRIS database while others draw from PPRTV, RAIS, or non-peer-reviewed compilations; such inconsistency systematically biases inter-study comparisons. To eliminate this source of variation, all *RfD* and *SF* values used in the present study were taken from a single authoritative source—the USEPA IRIS database—supplemented only where IRIS lacks entries by the USEPA Regional Screening Level Generic Tables. The full provenance of each value is given in the footnote to Table A1.

To address the conservative bias inherent in deterministic point-estimate risk assessment and to quantify the uncertainty propagation through the USEPA exposure model, we performed a Monte Carlo simulation (N = 10,000 iterations) treating soil concentration C, body weight *BW*, soil ingestion rate *IR*_ing_, inhalation rate *IR*_inh_, skin surface area *SA*, soil adherence factor *AF*, and exposure frequency *EF* as random variables drawn from probability distributions, rather than as fixed point values. Distribution choices follow USEPA Exposure Factors Handbook (EFH) 2011 recommendations:

(1) Soil concentration C: lognormal, with arithmetic mean and SD taken from the meta-analytic pooled estimates by continent and element (Section 3.2). Lognormality is consistent with the right-skewed empirical distribution of metal concentrations in urban surface soils. (2) Body weight *BW*, skin area *SA*, inhalation rate *IR*_inh_: truncated normal, with means equal to the deterministic values in Table 1 and a coefficient of variation of 20%; lower and upper truncation bounds were set to physiologically plausible limits (e.g., children’s *BW*: 8–30 kg). (3) Soil ingestion rate *IR*_ing_ and adherence factor *AF*: lognormal, with median equal to the Table-1 deterministic value and geometric standard deviation GSD = 1.5–1.6, capturing the right-skewed inter-individual variability documented in EFH Chapter 5. (4) Exposure frequency *EF*: triangular distribution with minimum 180, mode 350, and maximum 365 d·yr^−1^, reflecting the realistic range of indoor/outdoor activity scheduling. Reference doses (*RfD*s) and slope factors (*SF*s) were retained as deterministic regulatory anchors (Table A1) because their probabilistic representation would introduce circularity with the regulatory framework that the assessment is intended to inform. *ED*, *AT* and *PEF* were also fixed at their guideline values.

For each iteration, the Average Daily Doses for ingestion, dermal contact, and inhalation pathways (Equations (5)–(7)) were computed from the sampled inputs, and the Hazard Quotient *HQ*_i_ for each element i and the cumulative Hazard Index *HI* = *ΣHQ*_i_ were derived (Equations (8) and (9)). Results are reported as the 5th percentile (P5), median (P50), 95th percentile (P95), and the probability of exceeding the safety threshold P(*HI* > 1), separately for children and adults across the five continental groupings. A sensitivity analysis was performed by computing Spearman’s rank correlation coefficient ρ between each input variable and the resulting *HI* for the highest-risk subgroup (Asian children).

### 2.5. Assessment Criteria for Pollution and Risk

The classification standards for the calculated *I_geo_*, *P_N_*, and health risk indices are summarized in Table 2.

### 2.6. Statistical Analysis and Source Apportionment

Statistical analyses, including descriptive statistics and correlation matrices, were performed using SPSS 26.0 and Origin 2024 [114]. Spatial distributions and global mapping were conducted using ArcGIS 10.8.2.

## 3. Results

### 3.1. Database Characteristics and Geographic Coverage

The 91 included studies form a purposive sample of capital and core cities—the primary administrative, economic, and demographic hubs of each nation. Capital and core cities concentrate the largest exposure populations and carry the greatest policy relevance for urban soil management, and we judged that this purposive design was more appropriate for a risk-oriented synthesis than a probability sample of the global urban literature. Our pre-registered inclusion criteria (Section 2.2) require simultaneous measurement of ≥3 priority PTEs, ≥10 sampling sites per functional zone, presence of locally determined geochemical background, explicit QC/QA documentation, and availability of paired socioeconomic covariates. These criteria are the methodological foundation of the pooled effect-size analysis: relaxing them to enlarge k would introduce uncontrolled inter-study heterogeneity in digestion protocols, background-value conventions, functional-zone definitions, and QC/QA reporting, which in a quantitative meta-analysis produces a larger but less interpretable result. We considered this trade-off explicitly and chose methodological homogeneity over study volume. The consequence is that per-element k is markedly uneven across continental subsets: Asia (k = 18–36 per element) and Europe (k = 11–23 per element) provide the statistically robust core of the synthesis; the Americas (k = 3–7 for several elements), Africa (k = 4–15), and Oceania (k = 2 for all reported elements) provide smaller evidence bases. Throughout this paper, continental subsets with k ≥ 10 form the primary basis for pooled comparisons, hypothesis tests, and policy-relevant statements; subsets with smaller k are reported with their wide 95% confidence intervals and used only as illustrative context.

### 3.2. Global Pooled Concentrations and Heterogeneity Analysis

**Figure 2:**
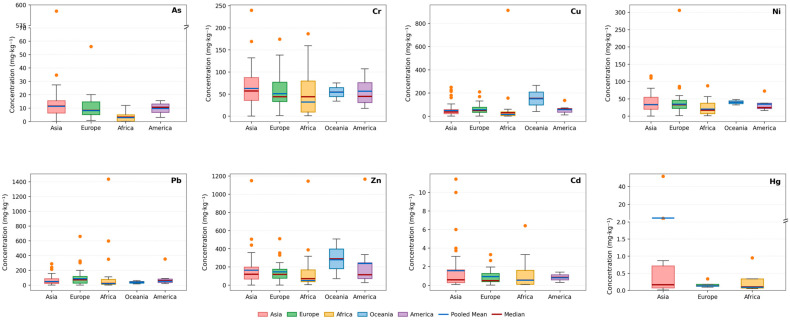
Global pooled concentrations and variability of eight PTEs in urban soils. The Oceanian subgroup is based on only k = 2 underlying studies for all reported elements; the apparently elevated means for Zn (280.3 mg·kg^−1^) and Cu (150.4 mg·kg^−1^) reflect the influence of two outlying observations from Fiji and should be interpreted as illustrative rather than statistically representative.

The global pooled concentrations and their associated variability for the eight PTEs are presented in Figure 2 and Table 3. The synthesized results indicated that the global pooled concentrations followed a descending order of: Zn (138.589 mg·kg^−1^) > Pb (56.969 mg·kg^−1^) > Cr (54.262 mg·kg^−1^) > Cu (46.998 mg·kg^−1^) > Ni (31.936 mg·kg^−1^) > As (8.563 mg·kg^−1^) > Hg (3.129 mg·kg^−1^) > Cd (1.228 mg·kg^−1^) (Figures S1–S8).

Extreme heterogeneity was observed across all eight elements, with *I*^2^ values consistently reaching 100%. This near-total variability is characteristic of global-scale urban soil surveys, reflecting the complex interplay between diverse lithogenic backgrounds and varying intensities of anthropogenic pressures (e.g., traffic emissions, industrial legacies, and waste management), which justifies the application of the random-effects model.

### 3.3. Subgroup Analysis: Intercontinental Disparities

Detailed forest plots for each continental subgroup across all elements are provided in the Supplementary Materials (Figures S9–S44).

Within the data-rich subsets, intercontinental disparities in urban-soil PTE accumulation are evident: Asian *I_geo_* values are highest for Cr, As, Cd, and Hg, while European cities show the highest Pb levels. Because *I_geo_* is normalized to local lithogenic background, these contrasts cannot be attributed to natural biogeochemical variation; they reflect—by construction—differences in industrialization stage and regulatory history between the two well-represented subsets. The illustrative observations for Africa, Oceania, and the Americas (Section 3.3.2) are reported with wide 95% confidence intervals (Table 3) and are not used to extend the ranking.

#### 3.3.1. Primary Findings from the Data-Rich Subsets (Asia and Europe)

Overall, widespread anthropogenic enrichment of PTEs was observed across all continents, though the magnitude of contamination and levels of heterogeneity varied significantly. Asia emerged as a primary hotspot, with seven of the eight studied metals (As, Cd, Cr, Cu, Ni, Pb, and Zn) exhibiting significant or extremely significant enrichment (*p* < 0.05). In contrast, Hg showed no statistical significance (*p* = 0.141), largely due to the influence of localized extreme values. Asian urban soils were characterized by exceptionally high between-study heterogeneity (*I*^2^ ≥ 99.8%), with the highest concentrations observed for Zn (163.738 mg·kg^−1^), followed by Cr (62.905 mg·kg^−1^) and Pb (49.185 mg·kg^−1^), reflecting intense anthropogenic inputs driven by rapid urbanization and industrialization.

In Europe, all eight elements showed extremely significant enrichment (*p* < 0.001) without substantial interference from outliers. Contamination levels followed the order: Zn (147.038 mg·kg^−1^) > Pb (87.694 mg·kg^−1^) > Cu (56.563 mg·kg^−1^) > Cr (51.142 mg·kg^−1^). Notably, European cities exhibited significantly higher Pb concentrations than other continents, a “legacy effect” likely attributed to historical accumulation from leaded gasoline and lead-based paints. Conversely, Hg concentrations (0.131 mg·kg^−1^) were much lower than the global average. It shows the efficacy of long-term pollution control policies.

#### 3.3.2. Continental Subsets with Smaller Evidence Bases (Illustrative)

The pooled estimates reported below for Africa, Oceania, and the Americas are based on smaller evidence bases (k = 4–15, k = 2, and k = 3–7 per element, respectively) and are presented for transparency and comparative reference. They are not used to support continental rankings, hypothesis tests, or policy recommendations in this paper, and their wide 95% confidence intervals (Table 3) should be borne in mind when reading the descriptions that follow.

Africa exhibited the lowest overall PTE concentrations among the studied regions, despite all eight metals showing extremely significant enrichment (*p* < 0.001) and high heterogeneity (*I*^2^ ≥ 96.4%). Accumulation levels ranked as Zn (51.128 mg·kg^−1^) > Cr (31.884 mg·kg^−1^) > Pb (21.073 mg·kg^−1^), while Hg (0.093 mg·kg^−1^) was the lowest in the world. This reflects lower levels of anthropogenic impact. These levels stay close to natural background levels.

Owing to the very small sample size in Oceania (k = 2 for all reported elements), inferences for this region are illustrative rather than statistically representative; pooled means are reported with their wide 95% CIs to convey this uncertainty (Table 3).

In the Americas, Cu, Pb, and Zn showed extremely significant enrichment (*p* < 0.001). As, Cr, and Ni showed significant enrichment (*p* < 0.05). However, Cd enrichment was not statistically significant (*p* = 0.138), and data for Hg were insufficient for robust meta-analysis. The Americas had a high Zn concentration (238.742 mg·kg^−1^), with Cu levels comparable to Europe and Pb levels similar to Asia. The extreme heterogeneity (*I*^2^ ≥ 93.4%) in this subgroup reflects the big developmental contrasts between North and Latin American urban cities.

Detailed *I_geo_* and *P_N_* values for all PTEs, cities, and functional zones are summarized in Table S2. The robustness of the *I_geo_*-based continental signals to lithogenic confounding is discussed in Section 4.5.1.

### 3.4. Evaluation of Publication Bias and Robustness

**Figure 3:**
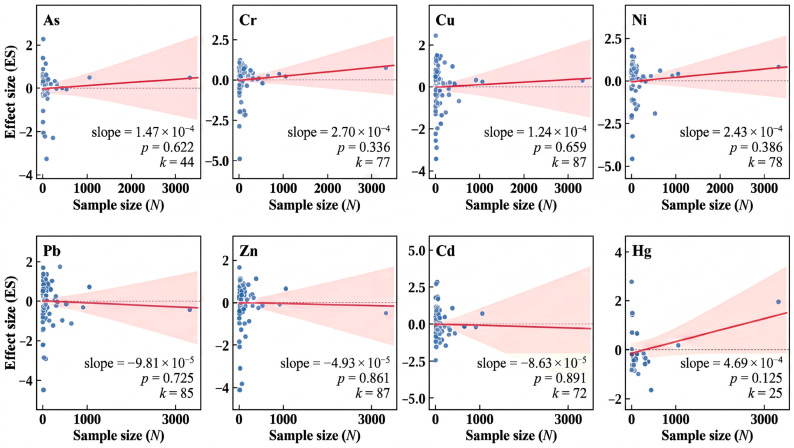
Assessment of publication bias for eight PTEs using regression-based *ES* analysis. The scatter plots illustrate the relationship between the number of samples and the standardized *ES* for each element across 91 studies. *ES*—Calculated based on the log-transformed mean concentrations of PTEs. Red line—Represents the linear regression trend.

The robustness of the meta-analysis was further assessed by regressing the standardized log-effect size (*ES*) of each study on its sample size. For all eight elements, the regression slope was indistinguishable from zero (|b_1_| ≤ 5 × 10^−4^; all *p* > 0.05; 95% *CI*s spanning zero), indicating no detectable sample-size-related publication bias (Figure 3).

To examine whether the observed pooled means and continental rankings are robust to methodological and temporal heterogeneity, two stratified sensitivity analyses were performed on the same DerSimonian–Laird random-effects framework as Table 3. Pooled means stratified by analytical technique (ICP-MS, ICP-AES/OES, AAS, XRF, Other) revealed substantial between-method differences for several elements (Figure S45). Between-method differences in pooled means reflect confounding between instrument choice and study site rather than systematic analytical bias, because the studies employing each technique are non-randomly distributed across cities and contamination levels.

Compress to one sentence: The potential confounding between analytical technique and site is partially mitigated by the *I_geo_* normalization, which applies a common analytical procedure to both numerator and denominator within a study, and by the wide 95% confidence intervals reported in Table 3.

Stratification by sampling decade (2000–2009, 2010–2019, 2020–2025; Figure S46) likewise revealed temporal evolution in some continental rankings, yet the four element-continent associations highlighted in this study remained robust: Asia ranked first or second for As in two of three decades, for Cr in all three decades, for Hg in two of three decades, and rose monotonically from rank 4 to rank 1 for Cd; Europe ranked first for Pb in two of three decades. The temporal mobility of rankings—particularly the rising Cd signal in Asia and the emerging As signal in the Americas—is itself consistent with hypothesis H1: had continental disparities been driven primarily by geochemical background, decadal rankings would have been static. The observed shifts therefore support, rather than undermine, the interpretation that anthropogenic loading and regulatory history are the dominant drivers.

### 3.5. Risk Assessment of PTEs in Urban Soils

#### 3.5.1. *I_geo_*-Based Pollution Levels of PTEs in Urban Soils

**Figure 4:**
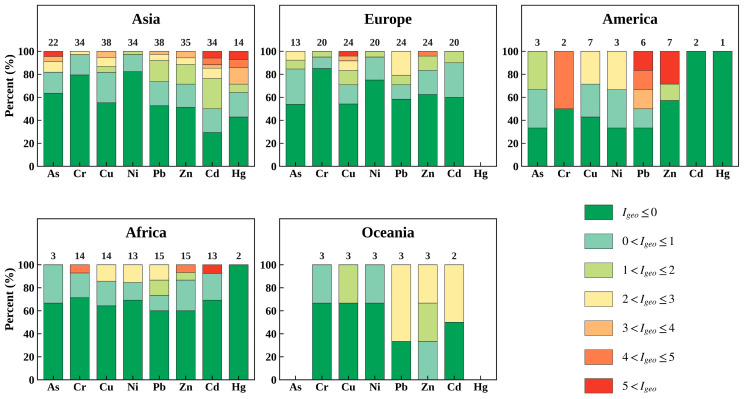
Proportional distribution of urban soil contamination levels across five continents based on the Geo-accumulation Index (*I_geo_*). The contamination degrees are categorized from “Unpolluted” to “Extremely polluted” according to the *I_geo_* classification scale. Sample sizes (k) are indicated above each stacked bar. Oceania (k = 2–3 per element) and Africa (k = 2–15 per element) categories should be interpreted with caution due to their limited sample sizes.

The *I_geo_* classification was employed to characterize the proportional distribution of PTEs across five continents under varying contamination levels (Figure 4). In this study, cities with *I_geo_* ≤ 2 were considered clean, whereas *I_geo_* > 2 was designated as the threshold for prioritized monitoring.

The global analysis reveals that over 80% of the investigated cities are subject to relatively low levels of PTE contamination:

- Oceania: With the exception of Pb, Zn, and Cd, the remaining elements remain near background levels, indicating that overall soil quality in this region is comparatively favourable.
- Europe: Contamination from Cr, Cu, and Cd appears limited. Most European cities exhibit moderate contamination; severe pollution is uncommon. These regions warrant closer surveillance.
- Americas: Contamination from As, Cd, and Hg remains comparatively limited, whereas Pb and Zn display substantial enrichment affecting more than 20% of the sites assessed, warranting closer surveillance.
- Asia and Africa: Asia exhibits the most pervasive enrichment footprint, with only Ni failing to show widespread severe contamination. In Africa, although As and Hg remain at baseline levels, occasional severe contamination events involving Pb and Cd—albeit infrequent—still warrant targeted monitoring owing to the potential for localized hotspots.

**Figure 5:**
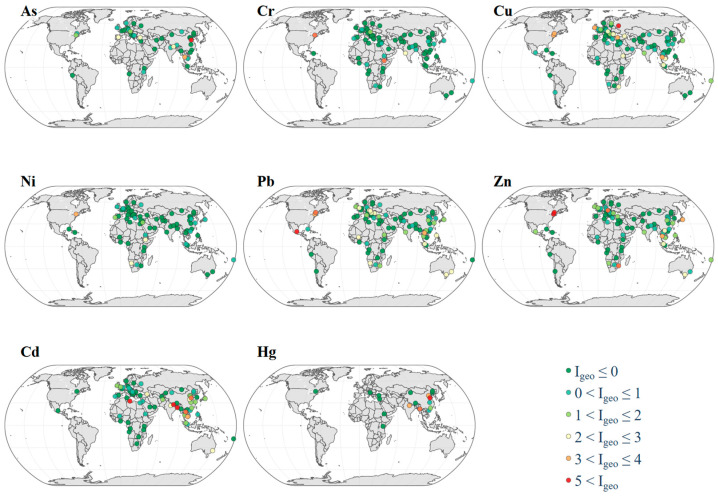
Global spatial distribution of *I_geo_* of individual PTE contamination hotspots in urban soils.

The spatial distribution of PTE contamination hotspots, defined by an *I_geo_* > 2, exhibited significant elemental and geographic heterogeneity (Figure 5).

We found that As, Cd, and Hg hotspots mostly stay in Asian cities. For example, Cd has very high *I_geo_* numbers in Kathmandu (7.06). Dhaka also shows a high level of 6.99. Hg enrichment is concentrated at sites such as Jinan (5.27) and Mandalay (4.10). The principal anthropogenic drivers are industrial atmospheric emissions, artisanal small-scale gold mining (for Hg specifically), and the cumulative legacy of long-term agrochemical inputs.

Within the data-rich Asian and European subsets, Pb and Zn are widely enriched (highest European Pb at Prague 2.74; highest Asian Pb at Vientiane 3.18; highest European Zn at Dublin 4.19; highest Asian Zn at Tokyo 3.12). For comparative reference, the highest city-level *I_geo_* values across the broader database appear in North American cities (Pb: Mexico City 5.67, Ottawa 4.82; Zn: Ottawa 6.08, New York 5.27), although these cities sit in the smaller-evidence-base subset and are reported for context only. Vehicular emissions, including dust from tire and brake-pad wear, are the dominant anthropogenic source across all five continents and reflect the global influence of road traffic on urban soil quality.

Cu and Ni hotspots occur in North America, Europe, and Southeast Asia, with Riga (5.51) and Bangkok (3.88) the most affected for Cu and New York (3.17) for Ni. These patterns reflect the combined influence of legacy industrial activity and contemporary transportation networks.

Cr exhibits a bifurcated spatial pattern: extreme values at Addis Ababa (4.78) reflect weathering of Cr-rich volcanic bedrock and are predominantly lithogenic, whereas the New York hotspot (4.32) is anthropogenically driven by historical metallurgical activity. This dual origin underscores that elemental *I_geo_* values must be interpreted in the context of local geology (Section 4.5.1).

In summary, within the data-rich subsets, industrial atmospheric emissions and vehicular exhaust are the dominant anthropogenic drivers, with Asia characterized by pronounced As, Cd, and Hg enrichment and Europe by legacy Pb accumulation. Elevated Pb and Zn observed in several North American cities (Section 3.3.2) are noted for comparative reference but are not part of the primary continental ranking. Effective mitigation will therefore require source-specific interventions targeting the locally dominant emission pathways.

#### 3.5.2. *P_N_*-Based Integrated Pollution Risk Patterns in Urban Soils

**Figure 6:**
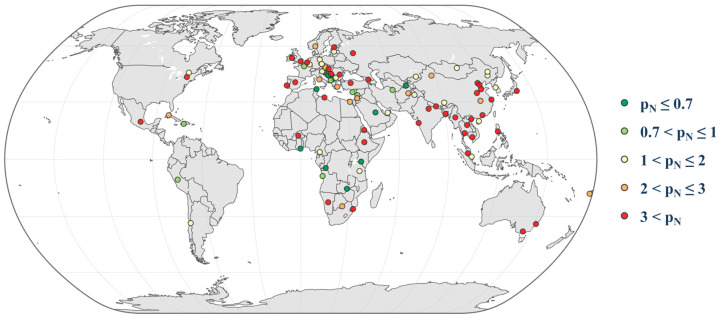
Global spatial distribution of *P_N_* of PTEs in urban soils.

The Nemerow integrated pollution index (*P_N_*) was employed to quantitatively evaluate the comprehensive pollution status of PTEs in global urban soils (Figure 6). In this assessment, cities with *P_N_* > 2 were identified as severely polluted areas requiring urgent mitigation.

Within the data-rich subsets, *P_N_* > 2 hotspots concentrate in East Asian industrial hubs (Beijing, Shanghai, Jinan, Tokyo) and South Asian metropolises (Kanpur, Mumbai), while European cities show comparatively fewer *P_N_* > 2 hotspots. Hotspots observed in legacy North American industrial centres (New York, Ottawa) and in African resource-extraction zones (Misrata, Addis Ababa) are reported in Figure 6 and Section 3.3.2 as illustrative observations from the smaller-evidence-base subsets and are not used in continental ranking.

Cities with the highest *P_N_* values are typically characterized by intensive heavy industry, high population density, and elevated energy consumption, where metallurgical operations and vehicular emissions co-deposit multiple PTEs onto urban soils. Inland cities situated in basins or deep river valleys (e.g., Jinan, Kathmandu) consistently exhibit higher *P_N_* values than their coastal counterparts, owing to the restricted atmospheric dispersion imposed by surrounding mountainous terrain, which prolongs pollutant residence time and enhances both dry and wet deposition. Elevated *P_N_* values further coincide spatially with mineral-extraction belts in South Asia and parts of North Africa, whereas industrialized nations continue to bear the legacy of historical activities, rapid contemporary industrialization imposes comparable pressure on developing regions; both trajectories must therefore be jointly considered when interpreting the global pattern of urban soil quality.

### 3.6. Human Health Risk Assessment Results 

#### 3.6.1. Non-Carcinogenic Risk

**Figure 7:**
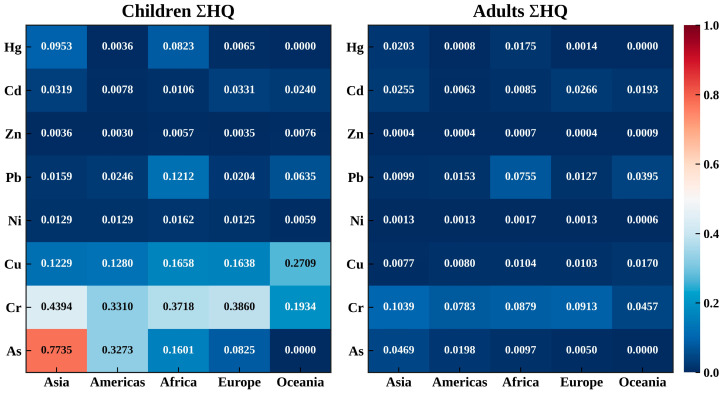
Global heatmap of non-carcinogenic health risks (*ΣHQ*) from potentially toxic elements (PTEs) in urban soils, stratified by children and adults across five continents. Cell values for Oceania (k = 2 underlying studies for all reported elements) and Africa Hg (k = 4) carry wider uncertainty than other cells; interpretation should focus on order-of-magnitude contrasts rather than absolute *HQ* values. Deterministic *ΣHQ* values in this heatmap represent point estimates at pooled mean concentrations; corresponding probabilistic results (Monte Carlo *P*50 and *P*(*HI* > 1)) are reported in Table A5 and Figure S47. Cell values for Oceania (*k* = 2 underlying studies for all reported elements) and Africa Hg (*k* = 4) carry wider uncertainty than other cells.

A non-carcinogenic risk assessment was conducted across three exposure pathways—inhalation, dermal contact, and hand-to-mouth ingestion—with pathway-specific exposure doses summarized in Table S3. Incidental soil-and-dust ingestion is the dominant pediatric exposure pathway in the USEPA framework, not because urban soils are used for food cultivation, but because the recommended residential *IR*_ing_ = 100 mg·d^−1^ for children integrates multiple behavioural routes—hand-to-mouth contact, direct soil mouthing during ground-level play, and ingestion of dust-contaminated food and beverages prepared in residential environments—documented in tracer, hand-rinse, dust-loading, and biomarker studies summarized in USEPA EFH (2011) Chapter 5 [115]. The spatial distribution of *HI* across global urban centres (Figure 7) shows that health risks were consistently higher for children than for adults on every continent (Table A2).

Across the data-rich Asian and European subsets, single-element *HQ* values were generally below 1 under the deterministic assessment. Within these subsets, the *HQ* for As in Asian children reached 0.773—the highest single-element pediatric value in the primary analysis and approximately 7.4 times the corresponding adult value. Adult *HQ*s were consistently below 0.15 in both subsets (Figure 7), confirming the disproportionate susceptibility of pediatric populations to soil-borne PTEs. Corresponding *HQ* values for the smaller-evidence-base subsets are shown in Figure 7 for transparency but are not used in this ranking.

It should be noted that the deterministic point estimates in Table A2 represent a regulator-aligned reference calculated at the pooled mean concentration under fixed central-tendency exposure parameters. The complementary Monte Carlo probabilistic assessment (Section 3.6.3) reveals that when the full empirical distributions of concentrations and exposure parameters are propagated, the cumulative *HI* at the median exceeds unity for children not only in Asia (*P*50 = 1.55) but also in Europe (*P*50 = 1.28) and the Americas (*P*50 = 1.29). The two assessment approaches are therefore complementary: Table A2 is the appropriate reference for single-value regulatory comparison, while Table A5 reflects the full probabilistic risk distribution. Both confirm that adults in all five continents face negligible non-carcinogenic risk, and that Asian children face the highest exceedance probability.

The dominant pediatric exposure pathway in the USEPA framework is incidental soil-and-dust ingestion (modelled as *ADD*_ing_), which encompasses hand-to-mouth contact, direct soil mouthing during ground-level play, and ingestion of dust-contaminated food and beverages under residential conditions. Incidental soil-and-dust ingestion is the dominant pediatric exposure pathway in the USEPA framework, not because urban soils are used for food cultivation, but because the recommended residential *IR*_ing_ = 100 mg·d^−1^ for children integrates multiple behavioural routes—hand-to-mouth contact, direct soil mouthing during ground-level play, and ingestion of dust-contaminated food and beverages prepared in residential environments, documented in tracer, hand-rinse, dust-loading, and biomarker studies summarized in USEPA EFH (2011) Chapter 5 [115]. The recommended *IR*_ing_ = 100 mg·d^−1^ for children reflects this integrated flux rather than any single behaviour. The pathway-specific Average Daily Doses in Table A3 indicate that *ADD*_ing_ exceeds *ADD*_inh_ by five to ten orders of magnitude for all eight PTEs in this study, reflecting the near-zero vapour pressure of non-volatile metals at ambient temperature. We refer readers to USEPA EFH (2011) Chapter 5 [115] for the underlying empirical basis.

The pathway-specific exposure doses underlying these results are summarized in Table A3, and the mechanistic implications for pediatric vulnerability are discussed in Section 4.4.

#### 3.6.2. Carcinogenic Risk

**Figure 8:**
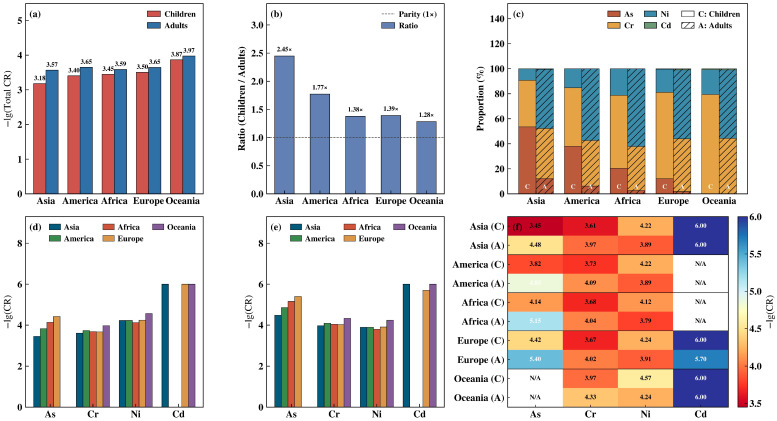
Carcinogenic risk (*CR*) of heavy metals via exposure pathways across five continents (Asia, America, Africa, Europe, and Oceania), compared between children and adults. All *CR* values are presented as −lg(*CR*); a smaller −lg(*CR*) value corresponds to a higher carcinogenic risk. (**a**) Total *CR* for children (brick red) and adults (steel blue) across continents; numerical values are labelled above each bar. (**b**) Ratio of children-to-adults total *CR* by continent; the dashed line marks parity (1×). (**c**) Stacked composition (%) of individual elements (As, Cr, Ni, Cd) in the total *CR* for each continent, with children (C, solid fill) on the left and adults (A, hatched fill) on the right of each pair. (**d**,**e**) Element-wise −lg(*CR*) by continent for children (**d**) and adults (**e**), respectively. (**f**) Heatmap of −lg(*CR*) for all continent–group–element combinations; warmer colours (red) indicate higher carcinogenic risk and cooler colours (blue) indicate lower risk. *Note:* As data are unavailable for Oceania (marked as N/A), Cd CR values equal to zero in America and Africa are treated as missing in the −lg transformation and shown as N/A.

Carcinogenic risk (*CR*) was assessed for four PTEs—As, Cr, Ni, and Cd—across all three exposure pathways (Table S4). Figure 8 shows that cumulative *CR* for the children’s group exceeded the 10^−4^ threshold across all five continents (*ΣCR* ranging from 1.36 × 10^−4^ in Oceania to 6.61 × 10^−4^ in Asia), indicating a significant carcinogenic risk requiring prioritized monitoring.

The relative contribution of the four metals to the total *CR* for children exhibited a continent-specific pattern. In Asia, where soil As concentrations are highest, As emerged as the dominant carcinogenic driver, followed by Cr (As > Cr > Ni > Cd; As contributing 53.5% of cumulative *CR* in Asian children). In the Americas, As and Cr were co-dominant (Cr > As > Ni > Cd), whereas in Africa, Europe, and Oceania, Cr remained the predominant driver (Cr > Ni > As > Cd). Cd consistently exhibited the lowest risk across all continents. Within the data-rich subsets, the cumulative *CR* for children was higher in Asia (6.61 × 10^−4^) than in Europe (3.13 × 10^−4^); both exceeded the 10^−4^ threshold. The corresponding illustrative values for the smaller-evidence-base subsets (Americas 3.95 × 10^−4^, Africa 3.57 × 10^−4^, Oceania 1.36 × 10^−4^) are reported in Figure 8 but are not used to rank the continental signal. In the adult group, the total *CR* similarly exceeded 10^−4^ across all continents, with the contribution sequence Ni > Cr > As > Cd reflecting the relatively lower *ADD*_ing_ in adults (longer averaging time, higher body weight) and the dominance of the dermal-inhalation pathway for Ni. Within the data-rich subsets, the cumulative adult *CR* was higher in Asia (2.71 × 10^−4^) than in Europe (2.24 × 10^−4^); illustrative values for the other subsets are given in Figure 8 (Americas 2.24 × 10^−4^, Africa 2.60 × 10^−4^, Oceania 1.07 × 10^−4^) and are not used in this ranking. Overall, *CR* values for children were consistently higher than those for adults—particularly for As, where the pediatric–adult *CR* ratio reached approximately 10-fold in Asia—corroborating that children exhibit greater age-dependent susceptibility to PTE exposure and may face more severe health implications. The elevation of As to the dominant carcinogenic driver in Asian children, in particular, underscores the public-health priority of pediatric-specific soil quality criteria for As in rapidly urbanizing Asian core and capitals.

#### 3.6.3. Probabilistic Risk Assessment

Monte Carlo probabilistic risk assessment (N = 10,000 iterations) confirmed and quantified the uncertainty around the deterministic findings of Section 3.6.1. The full set of *HI* percentiles and exceedance probabilities is reported in Table A5, and the corresponding probability density distributions are visualized in Figure S47.

For Asian children, the Monte Carlo median *HI* was 1.55 (P5–P95: 0.70–3.43), and the probability of exceeding the safety threshold, P(*HI* > 1), reached 81.9%. This is fully consistent with—and quantitatively strengthens—the deterministic point estimate of *HI* = 1.49 reported in Section 3.6.1, demonstrating that the central finding is not an artefact of conservative parameter choices but the median of a wide probability distribution. Within the data-rich subsets, the Monte Carlo results show that the elevated pediatric risk identified deterministically for Asia (Section 3.6.1) is paralleled in Europe: the median *HI* for European children was 1.28 with P(*HI* > 1) = 69.8%, driven primarily by legacy Pb. The illustrative Monte Carlo results for the smaller-evidence-base subsets—the Americas (P50 = 1.29; P(*HI* > 1) = 67.5%), Oceania (P50 = 1.19; P(*HI* > 1) = 62.4%), and Africa (P50 = 0.67; P(*HI* > 1) = 19.0%)—are reported in Table A5 for transparency but are not used to extend the continental ranking. Across all five subsets, no Monte Carlo iteration yielded *HI* > 1 for any adult subgroup.

For all five continents, the adult *HI* distributions lie entirely below *HI* = 1, with P(*HI* > 1) = 0.0%; no Monte Carlo iteration yielded *HI* > 1 for any adult subgroup. This near-binary contrast between children and adults across all continents (Figure S47) is a key probabilistic finding of the study: it demonstrates that pediatric vulnerability to soil-borne PTEs is robust to plausible parameter variation across the full distribution of exposure inputs, reinforcing the deterministic conclusions of Section 3.6.1 In other words, the headline conclusion that children, but not adults, face elevated cumulative non-carcinogenic risk in rapidly industrializing regions is a probabilistically robust finding rather than a methodological artefact.

The modest upward shift of MC P50 (1.55) relative to the deterministic *HI* (1.49) for Asian children—approximately 4%—reflects the non-linear aggregation of right-skewed inputs across eight elements and seven exposure variables (see reconciliation above).

The larger probabilistic upward shift for Europe and the Americas relative to Asia is consistent with the greater skewness of their Pb concentration distributions, as explained above.

The Monte Carlo median (P50) exceeds the deterministic point estimate (Table A2) for most continents—most notably for Europe and the Americas—because propagating right-skewed concentration and exposure-parameter distributions through a multiplicative risk model shifts the central tendency upward relative to a fixed point-estimate evaluation (analogous to Jensen’s inequality). The magnitude of this shift scales with the skewness of each continental Pb distribution: legacy-industrial hotspots such as Mexico City (*I_geo_* (Pb) = 5.67), Ottawa (4.82), and New York (5.27) drive pronounced upper-tail behaviour in Europe and the Americas, whereas Asia’s risk is driven by elevated mean concentrations across many studies (k = 18–36), yielding a smaller probabilistic shift. The two metrics are complementary: Table A2 provides the regulator-aligned reference for threshold comparison; Table A5 reflects population-level risk across the full input distribution.

#### 3.6.4. Sensitivity Analysis: Drivers of HI Variability

**Figure 9:**
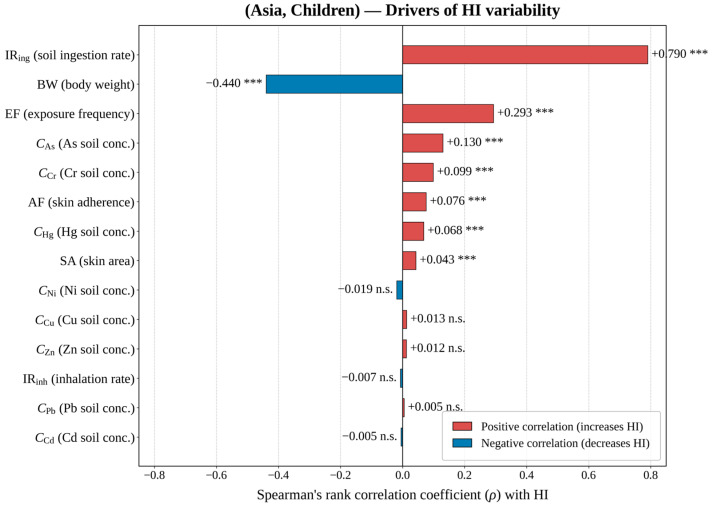
Tornado plot of input-parameter sensitivity for the Hazard Index (*HI*) of Asian children, computed as Spearman’s rank correlation coefficient ρ between each input variable and the simulated *HI* across 10,000 Monte Carlo iterations. Red bars indicate inputs that increase *HI* when increased (positive ρ); blue bars indicate inputs that decrease *HI* when increased (negative ρ). Significance levels: *p* < 0.001, *p* < 0.01, *p* < 0.05, n.s. = not significant. The soil ingestion rate *IR*_ing_ emerges as the dominant driver of *HI* variability (ρ = +0.79), followed by body weight *BW* (ρ = −0.44) and exposure frequency *EF* (ρ = +0.29). Among soil concentrations, As shows the strongest correlation with *HI* (ρ = +0.13)—consistent with the deterministic identification of As as the leading single-element risk driver in Asian children. Significance levels: *** *p* < 0.001; n.s. = not significant (*p* ≥ 0.05).

To identify which input variables most strongly drive the variability in *HI* for the highest-risk subgroup, Spearman’s rank correlation coefficients ρ between each input and the simulated *HI* were computed for Asian children (Figure 9).

The dominant driver was the soil ingestion rate *IR*_ing_ (ρ = +0.79, *p* < 0.001), followed by body weight *BW* (ρ = −0.44, *p* < 0.001) and exposure frequency *EF* (ρ = +0.29, *p* < 0.001). Among soil concentrations, As exhibited the strongest positive contribution (ρ = +0.13, *p* < 0.001), consistent with the deterministic identification of As as the leading single-element risk in Section 3.6.1, followed by Cr (ρ = +0.10) and Hg (ρ = +0.07). Soil concentrations of Pb, Zn, Cd, Cu, and Ni showed |ρ| < 0.02 and were not statistically significant drivers of *HI* variability—a result reflecting the orders-of-magnitude differences in element-specific *RfD*s (Table A1) rather than the ranking of soil concentrations.

This sensitivity profile carries direct policy implications. Because the dominant driver of pediatric *HI* variability is the soil ingestion rate (a behavioural and physiological parameter that is difficult to modify at the population scale), source reduction at the upstream end—lowering As and Cr concentrations in surface soils through targeted remediation—is the most leveraged intervention. Behavioural mitigation (reducing hand-to-mouth contact; promoting hand-washing) addresses only a single risk driver, whereas concentration reduction simultaneously lowers *HI* and reduces the probability mass above the safety threshold. This underscores the call in Section 5 for risk-based, source-targeted urban soil management.

### 3.7. Analysis of Influencing Factors on PTE Accumulation

PTE accumulation in urban soils is shaped by anthropogenic activities and environmental conditions, which together determine the spatial and temporal patterns of enrichment. This section examines three categories of driving forces: urban functional zonation, temporal trends, and demographic correlations.

#### 3.7.1. Impact of Urban Functional Zones

To evaluate the influence of land use on PTE enrichment, soil data from 12 representative cities across four continents were categorized into six functional zones: industrial, transportation, residential, commercial, agricultural, and urban green areas (Table A4). The results indicate a clear descending gradient of pollution intensity (*P_ij_*): industrial > transportation ≥ residential > commercial > agricultural > urban green areas.

Industrial and transportation zones exhibited the highest Pb, Zn, and Cr concentrations, reflecting metallurgical processes, machinery manufacturing, and vehicular emissions including tire and brake-pad wear [116]. The industrial zone in Zhengzhou, China, reached Zn = 683.54 mg·kg^−1^, directly attributable to local manufacturing. Agricultural and urban green areas generally showed lower contamination [117,118]; however, anomalous Cr enrichment in agricultural soils of Urumqi, China, indicates the influence of intensive agrochemical use and wastewater irrigation [119]. PTE concentrations generally decline with distance from industrial and transportation hubs, although a quantitative distance–decay function could not be derived from the heterogeneous source studies.

#### 3.7.2. Temporal Variations in PTE Concentrations

**Figure 10:**
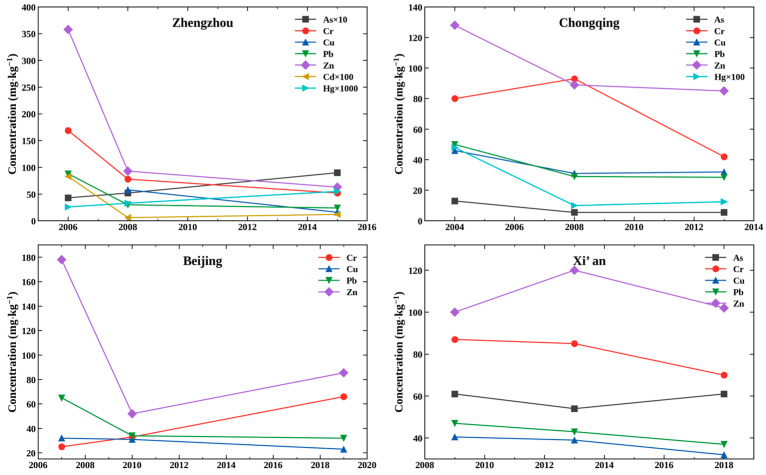
Temporal variations in the contents of potentially toxic elements (PTEs) in urban soils of selected major cities (Zhengzhou, Chongqing, Beijing, and Xi’an).

The relationship between PTE levels and urbanization history was analyzed using longitudinal data from four ancient Chinese cities with over 3000 years of history (Figure 10) [20,70,120,121,122,123,124,125,126,127,128,129]. Table S5 shows the full time-series dataset for four Chinese cities.

Most elements exhibit gradual declines over time, consistent with strengthened environmental regulations and the relocation of polluting industries; localized anomalies, however, persist:

- Urban Expansion: A transient increase in soil Zn was recorded in Beijing in 2019, attributable to accelerated infrastructure development and intensified traffic flow [130,131,132,133].
- Emergency Infrastructure Works: Elevated Zn and Cr concentrations observed in Xi’an in 2013 were associated with extensive post-disaster highway reconstruction, further exacerbated by concurrent dust and haze episodes [134,135,136].
- Hydrochemical Migration: A progressive rise in soil As was documented in Zhengzhou between 2006 and 2015. Given its high mobility under variable redox conditions, As is readily mobilized through domestic and industrial wastewater discharges and subsequently accumulates in receiving soils, particularly during periods of high precipitation [137,138,139].

#### 3.7.3. Correlation with Population Dynamics

**Figure 11:**
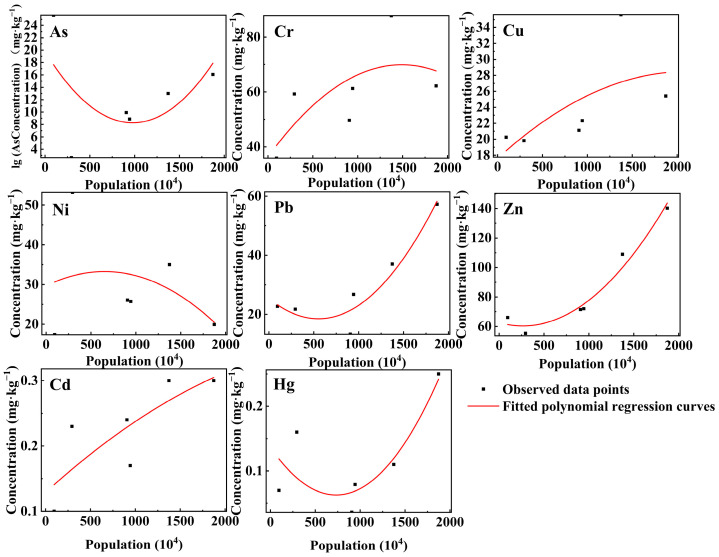
Polynomial regression relationships between concentrations of eight potentially toxic elements (PTEs: As, Cr, Cu, Ni, Pb, Zn, Cd, Hg) in urban soils and urban population size in six selected Chinese cities (Lhasa, Haikou, Changchun, Harbin, Wuhan, and Guangzhou). The red lines represent the fitted polynomial curves, and the black squares denote the observed data points.

Due to the limited availability of paired population–concentration time-series for non-Chinese cities, this sub-analysis is restricted to six Chinese megacities and should be interpreted as an illustrative case study rather than a global generalization. Figure 11 illustrates the relationship between urban population size and PTE concentrations across six Chinese cities (Lhasa, Haikou, Changchun, Harbin, Wuhan, and Guangzhou).

Concentrations of Pb, Zn, Cd, and Cu increase monotonically with population size, consistent with the corresponding intensification of industrial activity and vehicular emissions.

In contrast, Ni exhibits an inverse relationship with population size, a pattern attributable to structural resource scarcity and strategic national allocation [132,140,141,142,143,144,145,146,147,148,149,150]:

- Ni Strategic Use: Domestic Ni production in China is reserved primarily for high-end aerospace and defence applications (e.g., turbine blades, radar systems), restricting diffuse dispersion into common urban soils—unlike Pb.
- As Trend: As concentrations follow a U-shaped pattern, remaining elevated in both very small and very large cities but declining in mid-sized centres.

## 4. Discussion

### 4.1. Global Patterns and Continental Disparities of PTEs

This global meta-analysis reveals pronounced intercontinental disparities in urban soil PTE accumulation, which are intrinsically linked to varying stages of industrialization and the stringency of regional environmental regulations [151,152,153]. Asia currently exhibits a “heavy pollution” profile, particularly concerning Cd, Hg, and As. As the region has served as a global manufacturing hub for decades, intensive coal consumption and metallurgical activities have left a substantial “pollution legacy” in urban top soils [154,155,156,157,158]. Conversely, Oceania and parts of Europe present lower *I_geo_* and *P_N_* values. The efficacy of long-term environmental management, such as the early phase-out of leaded gasoline and the strict enforcement of industrial emission standards, is evident in these regions [159,160]. In the Americas, localized hotspots for Pb and Zn persist despite their transition to post-industrial economies [161]. This indicates that historical contamination, compounded by emissions from high-density vehicular traffic, continues to exert significant environmental pressure on modern urban ecosystems.

### 4.2. Anthropogenic Drivers Distribution Across Urban Functional Zones

The observed pollution gradient—industrial > transportation > residential > commercial > agricultural > green areas—highlights the direct impact of land-use optimization on soil quality [162]. The notable enrichment of Pb, Zn, and Cu in transportation zones serves as a global indicator of vehicular influence, primarily originating from tire wear, brake pad abrasion, and exhaust emissions [163]. While agricultural zones generally exhibit lower contamination levels, anomalous Cr enrichment in cities like Urumqi suggests the adverse impacts of prolonged, intensive agrochemical application and wastewater irrigation, which are established pathways for Cr and As accumulation [164,165,166]. Furthermore, topography significantly exacerbates pollution intensity; inland basin cities, such as Jinan and Kathmandu, display higher *P_ij_* values compared to coastal areas [167,168]. The surrounding mountainous terrain restricts atmospheric dispersion, particularly during stable meteorological conditions, leading to the localized accumulation of PTE-laden particulates via dry and wet deposition [169,170,171]. These findings emphasize the necessity of incorporating topographical and microclimatic considerations into urban planning and pollution mitigation frameworks [172].

### 4.3. Temporal Dynamics and Socioeconomic Drivers

Longitudinal analyses of ancient cities demonstrate a “decoupling” effect, indicating that economic growth and PTE accumulation do not necessarily progress in tandem. Generally, declining temporal trends in PTE concentrations validate the effectiveness of industrial relocation and enhanced waste management policies. However, localized anomalies exist. For instance, the escalating As concentrations in Zhengzhou can be attributed to specific hydrochemical mechanisms; As is highly mobile and prone to dissolution under fluctuating redox conditions or extreme precipitation events, facilitating its migration from industrial effluents into the soil matrix [173,174,175].

Additionally, the inverse relationship between Ni concentrations and population size in certain Chinese cities deviates from the conventional positive population–PTE relationship. As urban populations expand, Ni accumulation remains restricted, a phenomenon driven by structural resource scarcity and strategic national planning [176]. Unlike widespread pollutants such as Pb, Ni is prioritized for high-tech and military applications, thereby limiting its dissemination into general urban soils. This highlights that market supply and macro-industrial demands are critical socio-economic drivers of element-specific distribution, factors that are frequently overlooked in global PTE mapping [177,178,179,180].

### 4.4. Health Implications and the Vulnerability of Children

The most critical public-health finding from the probabilistic assessment is that the Monte Carlo median *HI* for children exceeds unity in four of five continents: Asia (*P*50 = 1.55; *P*(*HI* > 1) = 81.9%), Europe (*P*50 = 1.28; 69.8%), the Americas (*P*50 = 1.29; 67.5%), and Oceania (*P*50 = 1.19; 62.4%, noting the *k* = 2 study limitation). Asia exhibits both the highest absolute median *HI* and the highest exceedance probability. Africa remains below the threshold (*P*50 = 0.67; *P*(*HI* > 1) = 19.0%). The corresponding deterministic estimates (Table A2) show *HI* > 1 only for Asian children (1.49), while Europe (0.708) and the Americas (0.838) fall below the threshold—a difference attributable to the legacy Pb upper-tail concentration distributions in these continents (Section 3.6.3). Both metrics confirm that adults across all five continents face negligible non-carcinogenic risk (*P*(*HI* > 1) = 0.0%). The convergence of both assessment frameworks on Asia as the highest-risk continent, and their agreement that adults face no threshold exceedance, constitutes a robust, dual-method basis for the public-health conclusions of this study.

The carcinogenic risk assessment further corroborates this pattern: cumulative *CR* exceeded the 10^−4^ threshold in pediatric populations across all five continents, with As emerging as the dominant single-element carcinogenic driver in Asian children (53.5% of cumulative *CR*, Σ*CR*_As_ = 3.54 × 10^−4^). Children possess an inherent physiological and behavioural susceptibility to soil-borne PTEs [181,182]. Their higher respiration rates and enhanced gastrointestinal absorption ratios significantly amplify their internal toxicological dose. Behaviourally, frequent ground-level activities increase dermal and respiratory contact with contaminated soil, while total oral ingestion—the integrated flux of soil and dust entering the gastrointestinal tract via multiple behavioural routes including hand-to-mouth contact, direct soil mouthing during ground-level play, and ingestion of dust-contaminated food prepared in residential environments—constitutes the quantitatively dominant exposure pathway in the USEPA framework (Table A3; [115]). Chronic accumulation of As, Cr, and Pb can induce neurodevelopmental and carcinogenic outcomes that may be partially irreversible. These findings signal a critical need for a paradigm shift toward risk-based management, with prioritized focus on pediatric populations in rapidly urbanizing Asian megacities—while acknowledging that legacy Pb in European and American cities creates probabilistically elevated risk that warrants targeted monitoring [183].

Building on the pathway-resolved exposure analysis presented in Section 4.1, multiple independent epidemiological and toxicological lines converge on the consequences of soil-and-dust ingestion in children. An international pooled analysis by Lanphear and colleagues demonstrated an inverse exposure–response relationship between blood lead levels and IQ that remained significant at blood Pb levels well below 10 μg·dL^−1^ [184], and population-level analyses subsequently linked low-level Pb exposure to elevated cardiovascular mortality in adulthood [185]. For arsenic—identified by our sensitivity analysis as the dominant element-specific driver of pediatric *HI* in Asian core and capital cities—chronic low-dose exposure has been linked to deficits in IQ, memory, and executive function in children, with effects detectable at urinary As concentrations corresponding to soil exposures observed in the high-*I_geo_* Asian cities of our database [186,187]. The ATSDR Toxicological Profile for Arsenic similarly identifies developmental neurotoxicity as a critical effect endpoint underpinning the chronic oral *RfD*. For chromium, the IARC Working Group has classified inhaled hexavalent chromium as a Group 1 human carcinogen, with sufficient evidence for lung cancer in exposed workers [188]. These independent toxicological lines reinforce the soil-exposure findings of this meta-analysis and underscore the need for risk-based, child-centric soil quality criteria.

### 4.5. Limitations and Future Perspectives

Four limitations should be borne in mind when reading this synthesis. (1) The Asian and European subsets (k = 18–36 and k = 11–23 per element, respectively) are the only continental groupings on which we draw quantitative inferences; pooled values for the Americas (k = 3–7), Africa (k = 4–15), and Oceania (k = 2) are illustrative only. (2) The continental partitioning reflects socio-economic strata (industrialization stage and regulatory history), not geochemical units; per-city lithogenic variability is removed by *I_geo_* normalization before aggregation. (3) Definitive city-level source attribution would require Pb–Sr isotope analysis, sequential extraction, or PMF source apportionment, which were not consistently available across primary studies. (4) Our soil-total-Cr measurements do not resolve Cr(III)/Cr(VI) speciation, which is the toxicologically relevant distinction; this limits the precision with which the Cr signal can be linked to the toxicological literature.

While the present meta-analysis provides a comprehensive global synthesis, three methodological considerations warrant further discussion to guide future refinement. First, although we have moved beyond deterministic point-estimate risk assessment by implementing a 10,000-iteration Monte Carlo simulation (Section 2.4 and Section 3.6.3), the probabilistic model still assumes 100% gastrointestinal bioaccessibility of soil-bound PTEs, whereas in vivo and physiologically based extraction studies indicate that bioaccessibility for As and Pb in urban soils typically falls within 20–60%. Future probabilistic risk assessments should incorporate empirical bioaccessibility distributions, particularly for As, which our sensitivity analysis identified as the dominant element-specific driver of pediatric *HI* variability (Figure 9). Second, the limited availability of high-quality, long-term monitoring data in regions like Oceania (k = 2 studies) constrains the statistical power of intercontinental comparisons for specific elements. Third, although our toxicological reference values are drawn uniformly from USEPA IRIS to ensure inter-study consistency (Section 2.4), the IRIS *RfD*s themselves carry intrinsic uncertainty (typically a factor of 3–10). This component was intentionally excluded from the probabilistic framework to avoid circular logic with the regulatory thresholds that the assessment is designed to inform. Future research should prioritize the integration of localized, region-specific bioaccessibility data and the implementation of two-dimensional Monte Carlo techniques separating variability and uncertainty, both of which would refine the human health risk estimates and support the formulation of targeted, child-centric soil quality standards. However, even with the most conservative 20% bioaccessibility correction, the probability of *HI* > 1 among Asian children remains 28.4%, which still constitutes a precautionary signal warranting risk-based management.

#### 4.5.1. Lithogenic vs. Anthropogenic Sources

A potential confound in any global synthesis of urban soil PTEs is the contribution of natural biogeochemical provinces—regions where elevated soil concentrations reflect bedrock mineralogy rather than human activity. Examples include mafic and ultramafic terrains with naturally high Cr and Ni (e.g., parts of the East African Rift, the Indian Deccan, ophiolite belts), alluvial systems with geogenic As anomalies (e.g., the Bengal Basin), and carbonate weathering provinces with elevated Cd. Our use of the geo-accumulation index *I_geo_*, which normalizes each city’s concentrations to its locally determined background, partially mitigates this confound: where the local background was measured within the same lithological setting as the urban samples, the lithogenic component cancels in numerators and denominators. However, this cancellation may be incomplete where the reported ‘local background’ is established at a regional rather than city-specific lithological scale, or where the reference soil itself carries legacy anthropogenic loading—situations that we addressed during data extraction by adopting a hierarchical background-selection scheme (Section “Data Harmonization and Background-Value Selection”). The Cr enrichment we report for Addis Ababa (4.78), in particular, is documented to reflect weathering of Cr-rich volcanic bedrock [80] rather than industrial activity. We therefore caution that city-level *I_geo_* values for Cr, Ni, and locally As should be interpreted as joint indicators of lithogenic and anthropogenic loading, while the principal continental contrasts reported in this study—particularly the Asian As–Cd signal and the European Pb signal—are driven by industrially active cities outside known lithogenic anomalies and are therefore robust to this confound. A definitive city-by-city source attribution would require Pb–Sr isotope analysis, sequential extraction, or PMF source apportionment, which were not consistently available across the source studies; this is identified as a priority for follow-up city-scale geochemical work.

A further cross-disciplinary caveat applies to the Cr signal. The toxicologically relevant species is Cr(VI), whereas our pooled measurements are of soil total Cr, the great majority of which, under typical urban surface-soil redox conditions, is Cr(III). The HI and *CR* estimates we report for Cr therefore represent upper bounds on the toxicologically active fraction; reconciling our soil-total-Cr signal with the IARC Group 1 Cr(VI) classification will require coordinated speciation analysis in future primary studies.

#### 4.5.2. Geographic Representation and Analytical Heterogeneity

Geographic representation in the present synthesis reflects the distribution of primary studies meeting our pre-specified inclusion criteria (Section 2.2), with Asian and European cities contributing the majority of records and Oceanian and American cities comparatively fewer. While more than one hundred element-content studies exist for the United States alone, only a small subset met our pre-registered inclusion criteria—particularly the requirements for ≥3 priority PTEs simultaneously measured, ≥10 sites per functional zone, explicit QC/QA reporting, and presence of socioeconomic covariates. Future region-specific syntheses—particularly a North American synthesis built on the rich US literature—would complement the present global perspective. Analytical heterogeneity across primary studies is addressed in Section 3.4; future syntheses would benefit from coordinated round-robin reference-material analyses to explicitly deconvolve analytical and site-selection components of inter-study variability.

## 5. Conclusions

This meta-analysis tested four hypotheses on the global behaviour of urban-soil PTEs and arrived at the following findings:

- H1 supported: *I_geo_* normalization to local lithogenic background eliminates geochemical variability by construction, leaving industrialization-stage differences as the dominant explanation for the observed continental contrasts (Asian As-Cd-Cr-Hg enrichment; European Pb legacy). The decadal mobility of rankings (Figure S46) further corroborates an anthropogenic rather than lithogenic origin.
- H2 supported: A reproducible functional-zone gradient (industrial > transportation ≥ residential > commercial > agricultural > urban green) was confirmed across 12 cities on four continents.
- H3 supported for Asia; indicative for Europe and the Americas: Probabilistic risk assessment (Monte Carlo, N = 10,000) showed that the cumulative non-carcinogenic *HI* at the median exceeded the safety threshold for children in Asia (P50 = 1.55; P(*HI* > 1) = 81.9%; 95% *CI* 0.70–3.43; k = 18–36 per element), supporting the hypothesis for the most data-rich continental subset. The corresponding signals for European children (P50 = 1.28; 69.8%; k = 11–23 per element) and American children (P50 = 1.29; 67.5%; k = 3–7 for several elements) are directionally consistent but should be regarded as indicative pending synthesis of larger regional datasets. Findings for Oceania (k = 2) are reported in Table A5 and the main-text figures, with explicit small-sample warnings, and are not used to support the continental rankings or policy recommendations of this paper. Across all continental subsets, no Monte Carlo iteration yielded *HI* > 1 for any adult subgroup.
- H4 partially supported: While Pb, Zn, Cd, and Cu correlated positively with population size, Ni showed an inverse relationship attributable to strategic resource allocation rather than diffuse urban deposition.

We therefore tentatively suggest three directions for policy and future research, scaled to the evidence base of each region: (1) For rapidly urbanizing Asian core and capital cities, where the evidence base is strongest (k = 18–36 per element), the adoption of risk-based, child-specific soil quality criteria for As and Cr deserves prioritization by national environmental and public-health agencies. (2) For European core and capital cities (k = 11–23 per element), the directionally consistent pediatric HI signal supports targeted monitoring of legacy Pb hotspots, pending confirmation in regionally focused syntheses. (3) For under-represented regions, the immediate priority is expansion of the primary monitoring base under harmonized background-value and QC/QA reporting; analogous policy considerations should follow, not precede, that expansion.

## Figures and Tables

**Table 1:** Exposure parameters and toxicological indicators used for human health risk assessment.

Indices	Definition	Adult	Children	Unit	References
*BW*	Average weight	53.2	15.9	kg	[109,110]
*SA*	Skin Exposure Area	5700	2800	cm^2^	[109]
*IR*_inh_	Daily air intake	14.5	7.5	m^3^/d	[111]
*IR*_ing_	Daily intake rate	20	100	mg/d	[111]
*ED*	Duration of exposure	24	6	a	[109]
*EF*	exposure frequency	350	350	d/a	[112]
*AT*	Average exposure time (non-carcinogenic)	8760	2190	d	[111]
*PEF*	Particulate release factor	1.36 × 10^9^	1.36 × 10^9^	m^3^/kg	[111]
*AF*	skin adhesion factor	0.07	0.02	mg/cm^2^	[112]
*ABF*	skin absorption factor	0.03 for As and 0.001 for others.	0.03 for As and 0.001 for others.	dimensionless	[113]

**Table 2:** Classification standards for geo-accumulation index (*I_geo_*), Nemerow integrated pollution index (*P_N_*), and human health risk levels.

Indices	Range	Level
*I_geo_*	$I_{geo}$ ≤ 0	Uncontaminated
	0 < $I_{geo}$ ≤ 1	Uncontaminated to moderately contaminated
	1 < $I_{geo}$ ≤ 2	Moderately contaminated
	2 < $I_{geo}$ ≤ 3	Moderately to heavily contaminated
	3 < $I_{geo}$ ≤ 4	Heavily contaminated
	4 < $I_{geo}$ ≤ 5	Heavily to extremely contaminated
	5 < $I_{geo}$	Extremely contaminated
*P_N_*	*P_N_* ≤ 0.7	Unpolluted
	0.7 < *P_N_* ≤ 1.0	Pre-warning
	1.0 < *P_N_* ≤ 2.0	Slightly polluted
	2.0 < *P_N_* ≤ 3.0	Moderately polluted
	3.0 ≤ *P_N_*	Heavily polluted
*HI*	*HI* ≤ 1	No significant non-carcinogenic risk
	1 < *HI*	Potential non-carcinogenic risk
*CR*	*CR* < 10^−6^	Negligible carcinogenic risk
	10^−6^ ≤ *CR* ≤ 10^−4^	Acceptable or tolerable risk
	10^−4^ < *CR*	Significant carcinogenic risk

**Table 3:** Global pooled concentrations (mg·kg^−1^), 95% confidence intervals (CIs), and heterogeneity (*I*^2^) of eight PTEs in urban soils.

Continent	Parameters	As	Cr	Cu	Ni	Pb	Zn	Cd	Hg
Asia	Pooled Mean	11.316	62.905	48.275	33.319	49.185	163.738	1.560	4.834
	SD	4.466	5.192	4.710	1.857	5.798	15.224	0.537	3.287
	95% CI	[2.564, 20.069]	[52.730, 73.080]	[39.044, 57.507]	[29.679, 36.959]	[37.822, 60.548]	[133.901, 193.576]	[0.506, 2.613]	[−1.609, 11.277]
	*p*-value	0.011	<0.001	<0.001	<0.001	<0.001	<0.001	0.004	0.141
	*I* ^2^	100.0%	100.0%	99.9%	99.8%	100.0%	100.0%	100.0%	100.0%
	k	18	30	31	27	33	36	30	11
Europe	Pooled Mean	8.178	51.142	56.563	34.606	87.694	147.038	0.939	0.131
	SD	1.560	5.739	7.443	2.648	4.280	15.014	0.120	0.014
	95% CI	[5.120, 11.236]	[39.894, 62.390]	[41.975, 71.151]	[29.417, 39.796]	[79.306, 96.082]	[117.610, 176.466]	[0.704, 1.174]	[0.104, 0.158]
	*p*-value	<0.001	<0.001	<0.001	<0.001	<0.001	<0.001	<0.001	<0.001
	*I* ^2^	99.8%	100.0%	99.9%	99.9%	100.0%	100.0%	99.9%	92.1%
	k	11	17	22	21	23	22	18	4
Africa	Pooled Mean	2.919	31.884	14.643	20.387	21.073	51.128	0.536	0.093
	SD	0.373	2.033	1.203	1.300	2.158	3.256	0.052	0.020
	95% CI	[2.188, 3.650]	[27.900, 35.868]	[12.286, 17.001]	[17.840, 22.934]	[16.843, 25.303]	[44.746, 57.509]	[0.435, 0.637]	[0.054, 0.132]
	*p*-value	<0.001	<0.001	<0.001	<0.001	<0.001	<0.001	<0.001	<0.001
	*I* ^2^	99.9%	99.9%	99.8%	99.6%	99.4%	99.4%	99.6%	96.4%
	k	5	12	13	15	12	14	11	4
Oceania	Pooled Mean	—	54.201	150.446	39.780	37.335	280.283	—	—
	SD	—	20.498	112.824	7.789	21.648	217.826	—	—
	95% CI	—	[14.026, 94.376]	[−70.686, 371.578]	[24.515, 55.046]	[−5.093, 79.764]	[−146.648, 707.213]	—	—
	*p*-value	—	0.008	0.182	<0.001	0.085	0.198	—	—
	*I* ^2^	—	98.0%	97.9%	91.5%	98.4%	96.0%	—	—
	k	—	2	2	2	2	2	—	—
America	Pooled Mean	9.708	56.680	56.827	34.743	51.494	238.742	0.814	—
	SD	3.202	25.483	11.075	16.126	13.885	20.012	0.549	—
	95% CI	[3.432, 15.984]	[6.734, 106.626]	[35.121, 78.534]	[3.138, 66.349]	[24.281, 78.708]	[199.518, 277.966]	[−0.262, 1.889]	—
	*p*-value	0.002	0.026	<0.001	0.031	<0.001	<0.001	0.138	—
	*I* ^2^	100.0%	100.0%	100.0%	100.0%	100.0%	100.0%	93.4%	—
	k	3	3	7	4	5	7	2	—
Global	Pooled Mean	8.563	54.262	46.998	31.936	56.969	138.589	1.228	3.129
	SD	1.286	3.075	2.300	1.846	2.814	4.300	0.212	1.444
	95% CI	[6.043, 11.083]	[48.234, 60.289]	[42.489, 51.506]	[28.318, 35.555]	[51.453, 62.484]	[130.162, 147.017]	[0.812, 1.643]	[0.298, 5.960]
	*p*-value	<0.001	<0.001	<0.001	<0.001	<0.001	<0.001	<0.001	<0.001
	*I* ^2^	100.0%	100.0%	100.0%	100.0%	100.0%	100.0%	93.4%	100.0%
	k	38	68	77	67	75	78	61	21

Pooled Mean—random-effects pooled mean; SD—standard error; 95% CI—95% confidence interval; *p*-value—Tests the null hypothesis that the pooled mean = 0. *p* < 0.05 confirms the statistical significance of the pooled effect size; *I*^2^—heterogeneity index (%); k—number of studies included; — = insufficient data (k < 2). Method: DerSimonian–Laird random-effects model.

## Data Availability

No new data were created or analyzed in this study. Data sharing is not applicable to this article.

## Supplementary Materials

The following supporting information can be downloaded at https://www.mdpi.com/article/10.3390/toxics14060496/s1, Figures S1–S8: Forest plot of meta-analysis for 8 priority potentially toxic elements (PTEs: As, Cr, Cu, Ni, Pb, Zn, Cd, Hg) concentrations in global urban soils, with a total of 8 independent plots; Figures S9–S44: present the forest plots of the meta-analysis results for 8 priority potentially toxic elements (PTEs: As, Cr, Cu, Ni, Pb, Zn, Cd, Hg) in urban soils across 5 continents (Asia, Europe, Africa, Americas, Oceania), with a total of 36 independent plots; Figure S45: Method-stratified sensitivity of pooled PTE concentrations. Inverse-variance-weighted random-effects pooled means by analytical technique (ICP-MS, ICP-AES/OES, AAS, XRF, Other) for the eight priority PTEs; Figure S46: Decadal sensitivity of continental pollution rankings. Continental rankings of inverse-variance-weighted pooled means by sampling decade (2000–2009, 2010–2019, 2020–2025); Figure S47: Probability density distributions of the cumulative Hazard Index (*HI*) from Monte Carlo simulation (N = 10,000 iterations), stratified by age group and continent. Table S1: List of Global Core and Capital Cities and Metadata on PTEs Concentrations; Table S2: Geo-accumulation Index (*I_geo_*) and Nemerow Composite Pollution Index (*P_N_*) of PTEs in Urban Soils; Table S3: Non-Carcinogenic Health Risk Indices (*HQ/HI*) for Children and Adults Based on US EPA Model; Table S4: Carcinogenic Risk (*CR*) of PTEs for Children and Adults Based on US EPA Model; Table S5: Temporal Variations in PTEs Concentrations in Selected Urban Soils.

## Abbreviations

The following abbreviations are used in this manuscript:

*ADD*	Average Daily Dose
As	Arsenic
Cd	Cadmium
CI	Confidence Interval
Cr	Chromium
Cu	Copper
*CR*	Carcinogenic Risk
*HI*	Hazard Index
*HQ*	Hazard Quotient
Hg	Mercury
HHRA	Human Health Risk Assessment
*I*^2^	Heterogeneity Index
*I_geo_*	Geo-accumulation Index
Ni	Nickel
Pb	Lead
*P_ij_*	Nemerow Pollution Index for element *i* in functional zone *j*
*P_N_*	Nemerow Integrated Pollution Index
PRISMA	Preferred Reporting Items for Systematic Reviews and Meta-Analyses
PTEs	Potential Toxic Elements
USEPA	United States Environmental Protection Agency
Zn	Zinc

## Appendix A

**Table A1:** Reference dose (*RfD*) and slope factor (*SF*) values for potentially toxic elements (PTEs) used in the health risk assessment, categorized by exposure pathway.

PTEs	*RfD*			*SF*		
	*RfD*_inh_ (mg·kg^−1^·d^−1^)	*RfD*_der_ (mg·kg^−1^·d^−1^)	*RfD*_ing_ (mg·kg^−1^·d^−1^)	*SF*_inh_ (kg·d·mg^−1^)	*SF*_der_ (kg·d·mg^−1^)	*SF*_ing_ (kg·d·mg^−1^)
Hg	8.57 × 10^−5^	2.10 × 10^−5^	3.00 × 10^−4^	—	—	—
Cd	1.00 × 10^−5^	1.00 × 10^−5^	1.00 × 10^−3^	6.30	6.10	0.0061
As	1.230 × 10^−4^	1.230 × 10^−4^	3.00 × 10^−4^	15.10	1.50	1.5
Pb	3.52 × 10^−3^	5.25 × 10^−4^	3.50 × 10^−3^	—	—	—
Cr	2.86 × 10^−5^	6.00 × 10^−5^	3.00 × 10^−4^	42.00	20.00	0.5
Ni	2.06 × 10^−2^	5.40 × 10^−3^	2.00 × 10^−2^	0.84	42.50	—
Cu	4.02 × 10^−2^	1.20 × 10^−2^	3.00 × 10^−3^	—	—	—
Zn	3.00 × 10^−1^	6.00 × 10^−2^	3.00 × 10^−1^	—	—	—

All *RfD* and *SF* values were drawn from the USEPA Integrated Risk Information System, where available. *RfD*_inh_ for Pb, Zn, Cu and Ni were calculated from the corresponding USEPA *RfC* values using the standard conversion *RfD*_inh_ = *RfC* × *IR*_inh_/*BW* with adult parameters (*IR*_inh_ = 20 m^3^/d, *BW* = 70 kg) per the USEPA RAGS framework. *RfD*_der_ were derived from *RfD*_ing_ using gastrointestinal absorption factors (GIABS) per USEPA RAGS Part E (2004): GIABS = 1.0 for As, Cd, Cu, Ni, Pb, Zn and Hg, and GIABS = 0.025 for Cr (yielding the lower *RfD*_der_ for Cr). The oral *RfD* for Cr is based on the 1998 IRIS Cr(VI) value (3 × 10^−3^ mg·kg^−1^·d^−1^) adjusted by an assumed 10% Cr(VI) fraction in soil total Cr. ‘—’ indicates that no value is available in IRIS or carcinogenicity is not classified.

**Table A2:** Global comprehensive index for non-carcinogenic risk of PTEs in soils.

Index	Exposed Population	Asia	America	Africa	Europe	Oceania
*HI*	Children	1.49	8.38 × 10^−1^	9.34 × 10^−1^	7.08 × 10^−1^	5.65 × 10^−1^
	Adults	2.16 × 10^−1^	1.30 × 10^−1^	2.12 × 10^−1^	1.49 × 10^−1^	1.23 × 10^−1^

**Table A3:** Global average exposure dose of PTEs via different exposure routes in urban soils.

Exposed Population	Ingestion Mode	As	Cr	Cu	Ni	Pb	Zn	Cd	Hg
Children	*ADD*_ing_	1.00 × 10^−4^	3.15 × 10^−4^	5.10 × 10^−4^	2.37 × 10^−4^	1.25 × 10^−3^	1.37 × 10^−3^	1.38 × 10^−5^	1.30 × 10^−5^
	*ADD*_inh_	1.28 × 10^−14^	2.22 × 10^−14^	4.48 × 10^−14^	1.39 × 10^−8^	2.24 × 10^−8^	5.87 × 10^−8^	1.13 × 10^−9^	1.46 × 10^−9^
	*ADD*_der_	3.90 × 10^−6^	2.25 × 10^−6^	4.54 × 10^−6^	1.78 × 10^−6^	1.73 × 10^−5^	1.24 × 10^−5^	1.19 × 10^−7^	1.48 × 10^−7^
Adults	*ADD*_ing_	6.02 × 10^−6^	1.88 × 10^−5^	3.05 × 10^−5^	1.42 × 10^−5^	7.48 × 10^−5^	8.18 × 10^−5^	8.23 × 10^−7^	7.79 × 10^−7^
	*ADD*_inh_	7.39 × 10^−15^	1.28 × 10^−14^	2.59 × 10^−14^	1.01 × 10^−14^	9.84 × 10^−14^	7.05 × 10^−14^	6.77 × 10^−16^	8.43 × 10^−16^
	*ADD*_der_	8.30 × 10^−6^	4.79 × 10^−6^	9.68 × 10^−6^	3.79 × 10^−6^	3.68 × 10^−5^	2.64 × 10^−5^	2.51 × 10^−7^	3.16 × 10^−7^

**Table A4:** Concentrations of potentially toxic elements (PTEs) and Nemerow comprehensive pollution index (*P_ij_*) in urban soils across different functional areas and global cities (mg·kg^−1^).

Cities	Cr	Cu	Ni	Pb	Zn	Cd	Functional Zones	(*P_ij_*)
Gaborone, Botswana	79.00	35.00	51.00	50.00	175.00	1.30	R	1.55
	73.00	37.00	44.00	74.00	250.00	1.60	I	2.05
	72.00	36.00	48.00	112.00	248.00	1.60	C	2.11
	136.00	69.00	121.00	36.00	121.00	2.00	A	2.99
Mexico City, Mexico	—	43.50	—	49.70	195.80	1.10	U	2.20
	—	98.20	—	1188.90	741.70	1.60	T	107.49
Havana, Cuba	—	105.00	69.00	140.00	292.00	—	I	2.15
	—	87.00	65.00	49.00	161.00	—	U	1.16
Lisbon, Portugal	49.60	—	37.90	6.40	—	0.39	T	2.19
	39.40	—	73.40	6.90	—	0.72	R	5.11
	50.90	—	39.80	2.10	—	0.22	U	2.17
Bratislava, Slovakia	32.30	54.00	20.40	38.90	149.00	—	T	2.26
	44.10	40.90	25.60	32.30	109.00	0.30	R	1.50
Budapest, Hungary	25.44	25.85	17.74	170.5	33.73	—	I	5.01
	147.66	32.63	28.78	217.93	48.23	—	U	6.58
	138.19	56.24	28.20	302.94	47.11	—	R	9.03
Changchun, China	51.10	19.11	28.11	54.01	135.45	0.28	I	1.71
	53.77	25.92	28.76	68.03	146.17	0.32	T	1.90
	44.21	17.58	22.54	33.78	117.51	0.18	U	1.39
	48.90	20.25	25.35	38.97	138.15	0.21	R	1.63
	48.15	17.03	24.92	26.97	132.94	0.21	C	1.54
Shanghai, China	362.00	73.00	58.00	128.00	362.00	0.50	I	4.64
	134.00	64.00	55.00	161.00	239.00	0.34	T	3.28
	98.00	44.00	55.00	101.00	165.00	0.29	R	2.34
	111.00	39.00	55.00	81.00	158.00	0.22	R	2.16
Tianjin, China	78.00	44.00	34.00	38.00	120.00	0.26	U	1.67
	77.00	40.00	32.00	36.00	120.00	0.47	T	1.82
	91.00	65.00	34.00	74.00	241.00	0.40	I	2.96
	77.00	40.00	32.00	37.00	128.00	0.29	R	1.73
Zhengzhou, China	151.73	25.87	8.95	87.80	112.89	1.20	C	3.06
	177.67	31.84	11.94	133.32	683.54	1.06	I	7.41
	185.16	28.01	14.59	76.16	877.21	0.79	R	8.97
	199.91	15.08	9.96	131.3	638.98	1.25	T	7.09
Jinan, China	61.13	18.76	47.38	19.05	119.27	2.58	U	8.45
	49.12	21.91	59.02	53.74	120.87	3.37	T	2.20
	70.35	30.84	71.56	26.96	81.45	4.03	I	7.72
	49.65	26.79	51.49	80.33	244.29	4.83	C	16.97
	75.85	26.99	38.69	74.59	127.73	4.45	A	10.30
Urumqi, China	18.00	11.00	8.90	9.68	27.60	0.06	A	1.40
	47.00	32.00	38.00	28.30	89.90	0.25	U	1.32
	47.00	35.00	37.00	37.80	103.00	0.29	R	1.49

R—Residential; I—Industrial; A—Agricultural; U—Urban green area; T—Transportation; C—Commercial.

**Table A5:** Probabilistic estimates of the cumulative Hazard Index (*HI*) for global urban soil PTEs by continent and age group, based on Monte Carlo simulation (*N* = 10,000 iterations). *P*5, *P*50 (median), and *P*95 are the 5th, 50th, and 95th percentiles of the simulated *HI* distribution; *P*(*HI* > 1) is the percentage of iterations that exceeded the safety threshold of unity. Values differ from the first-round submission due to corrected parameterization fully consistent with USEPA EFH (2011); see Section 3.6.3 for reconciliation with the deterministic estimates of Table A2.

Continent	Age Group	P5	P50 (Median)	Mean	P95	P(*HI* > 1) [%]
Asia	Children	0.70	1.55	1.74	3.43	81.9
Asia	Adults	0.06	0.12	0.13	0.25	0.0
Europe	Children	0.58	1.28	1.44	2.83	69.8
Europe	Adults	0.05	0.10	0.11	0.20	0.0
Africa	Children	0.30	0.67	0.74	1.44	19.0
Africa	Adults	0.02	0.05	0.05	0.10	0.0
America	Children	0.51	1.29	1.51	3.26	67.5
America	Adults	0.04	0.10	0.11	0.23	0.0
Oceania	Children	0.48	1.19	1.41	3.10	62.4
Oceania	Adults	0.03	0.08	0.09	0.19	0.0

The Asian-children row represents the headline finding (*HI* > 1 in 81.9% of MC iterations). The deterministic point estimate of *HI* = 1.49 (Table A2) falls within the interquartile range of the probabilistic distribution (*P*50 = 1.55; 95% CI = 0.70–3.43), confirming internal consistency. *P*(*HI* > 1) = “0.0” indicates that no single iteration of the 10,000 produced *HI* > 1 for any adult subgroup. Oceania estimates inherit the small-sample limitation discussed in Section 3.3 (*k* = 2 underlying studies); interpret with caution. Revised Figure S47 (attached) displays the full probability density distributions corresponding to this table.
