# Supplementary material for: Spatiotemporal Dynamics and Human Health Risk Assessment of Potentially Toxic Elements in Global Urban Soils: A Systematic Meta-Analysis
Source: Toxics. 2026 Jun 7;14(6):496. doi: 10.3390/toxics14060496 (PMC13307911; doi:10.3390/toxics14060496)
Supplement: Supplementary file 1 [file toxics-14-00496-s001.zip › toxics-4296084-supplementary.pdf]

Supplementary Materials

Spatiotemporal Dynamics and Human Health Risk Assessment of Potentially Toxic Elements in Global Urban Soils: A Systematic Meta-Analysis

This file includes：

A. Supplementary figures

Figures S1 to S8 (global)

Figures S9 to S44 (continental)

Figures S45 to S46(stratified sensitivity analyses)

Figures S47(Monte Carlo simulation)

B. Supplementary tables

Table S1 to S5

A. Supplementary figures

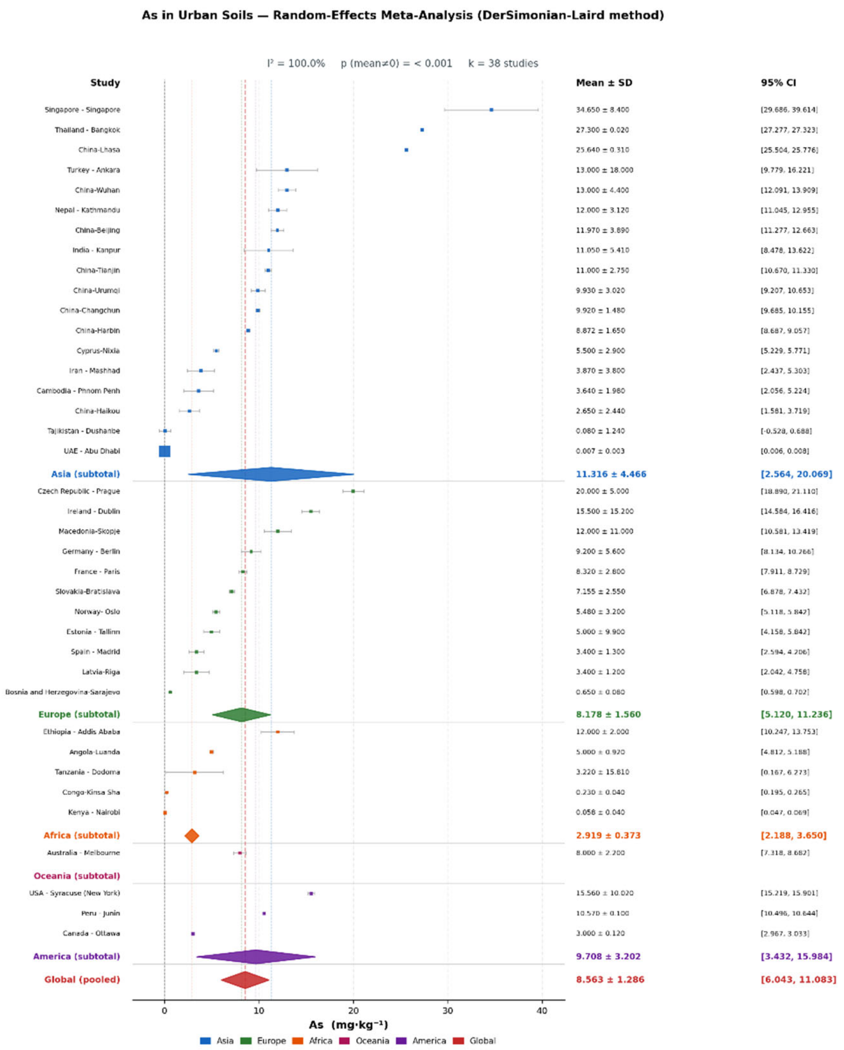

Figure S1. Forest plot of meta-analysis for As concentrations in global urban soils.

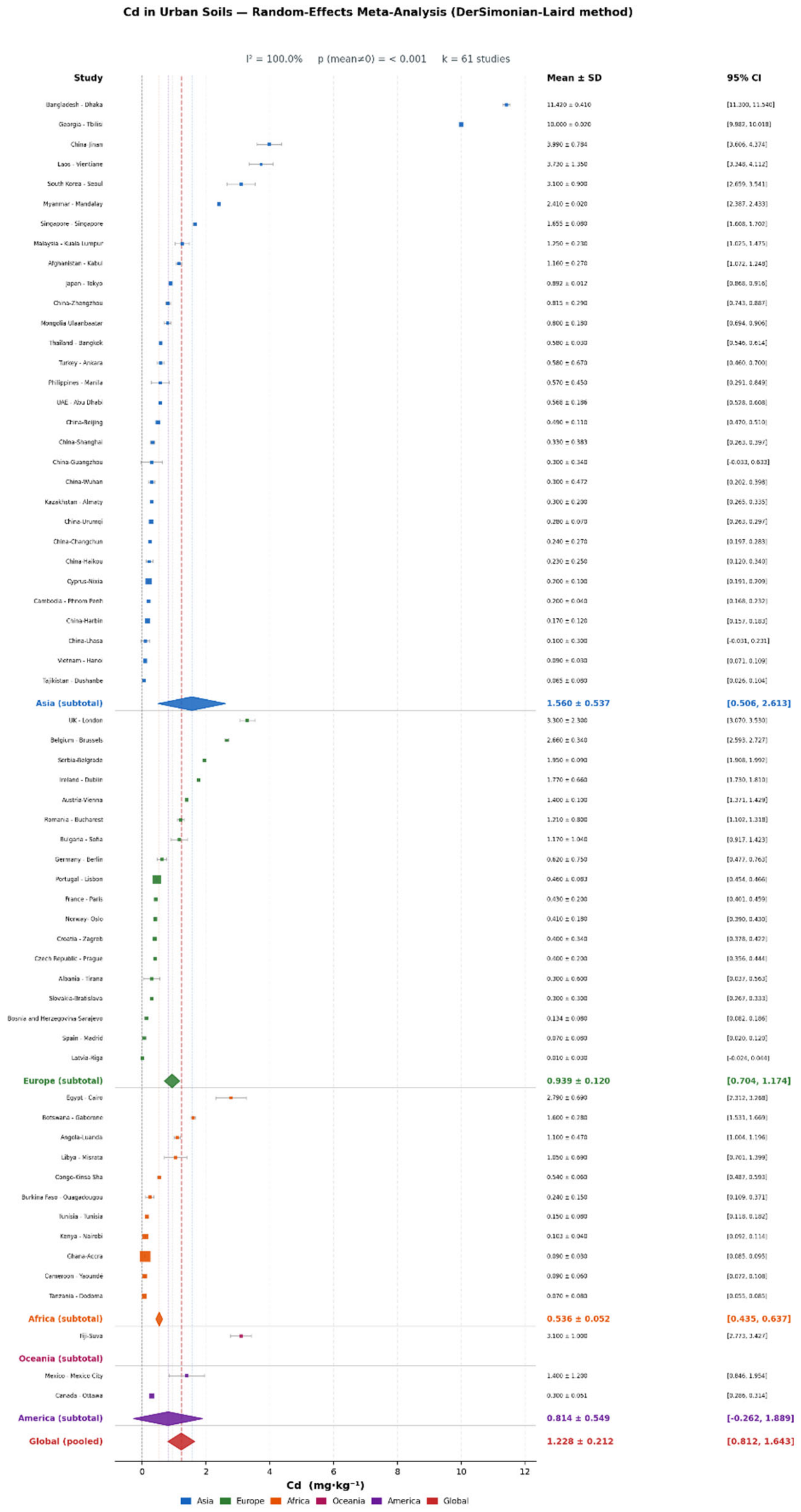

Figure S2. Forest plot of meta-analysis for Cd concentrations in global urban soils.

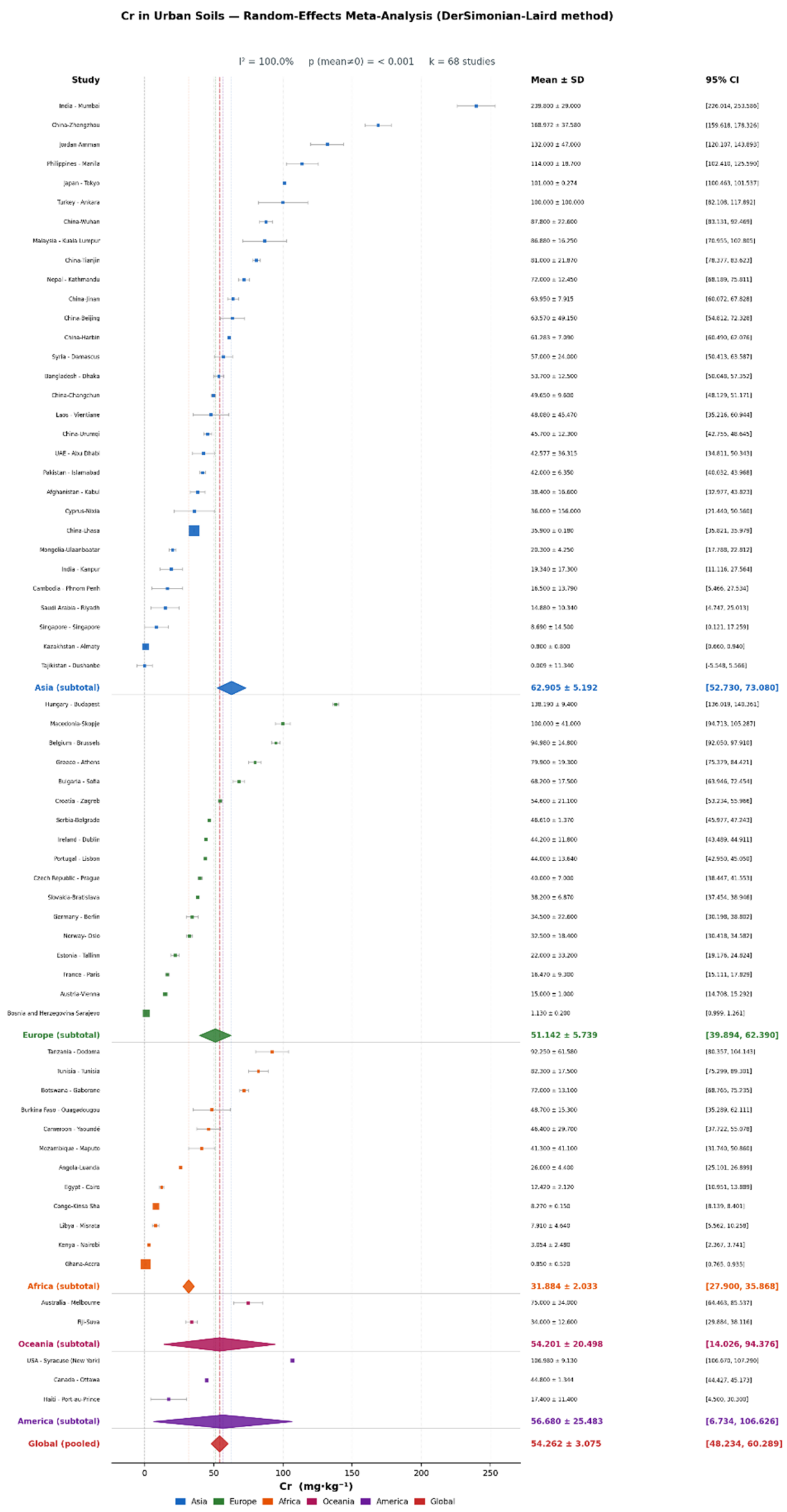

Figure S3. Forest plot of meta-analysis for Cr concentrations in global urban soils.

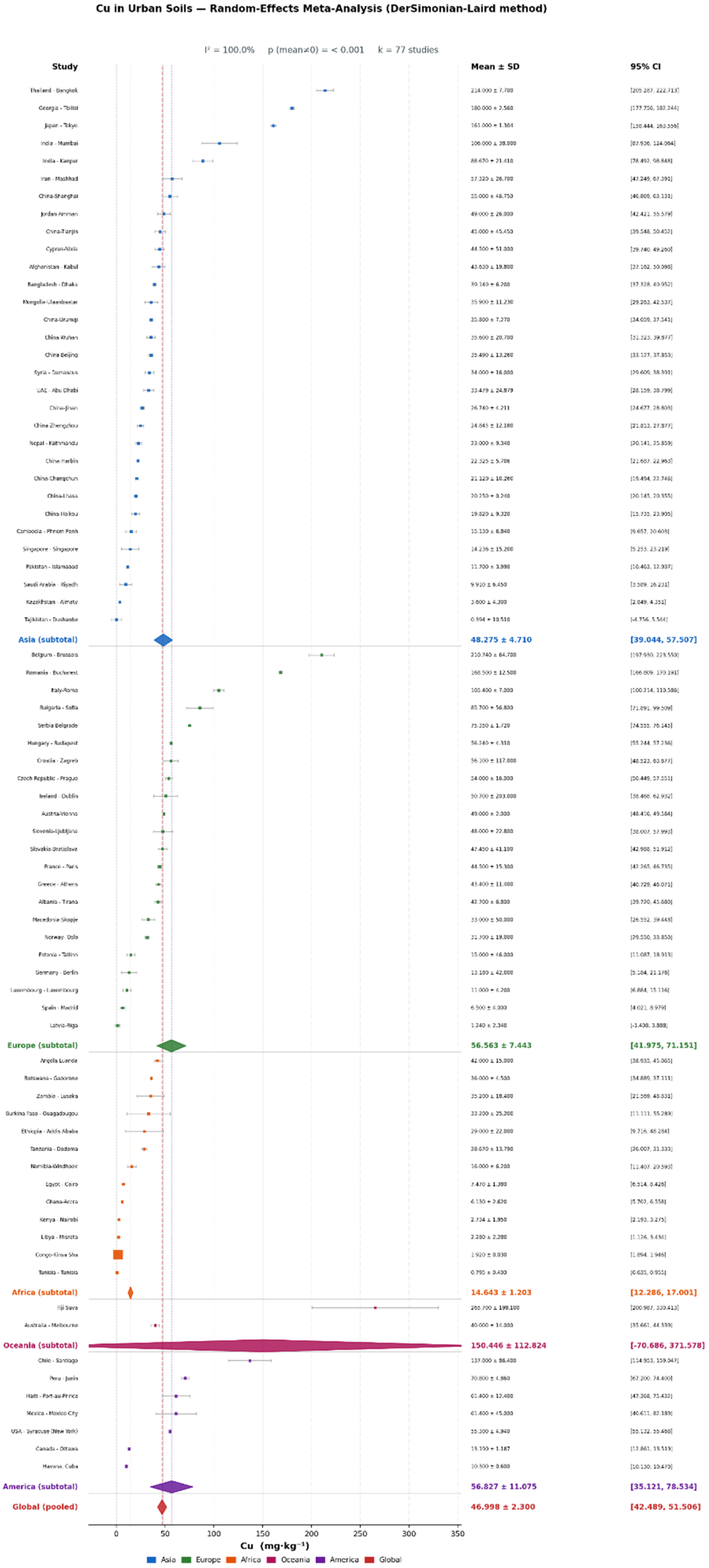

Figure S4 Forest plot of meta-analysis for Cu concentrations in global urban soils.

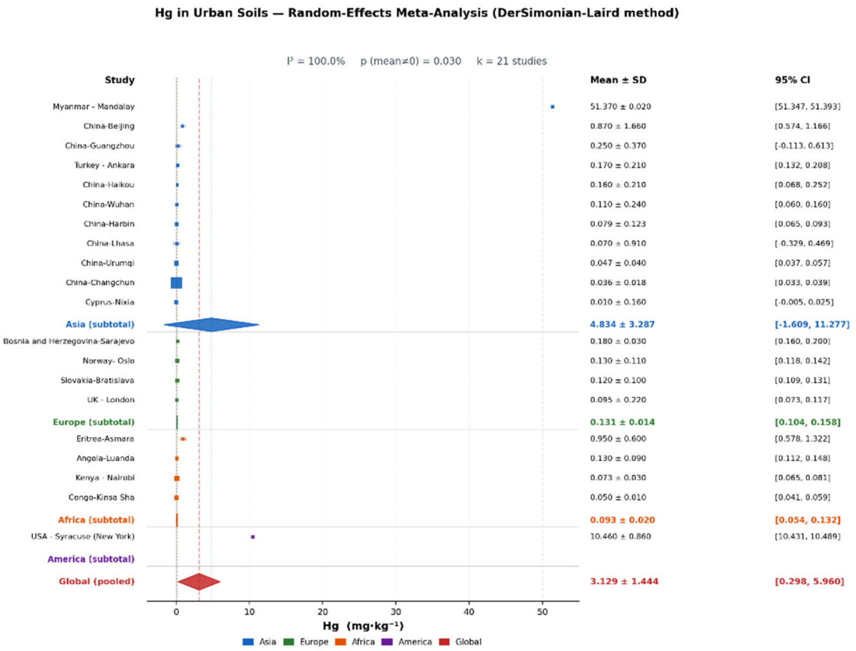

Figure S5. Forest plot of meta-analysis for Hg concentrations in global urban soils.

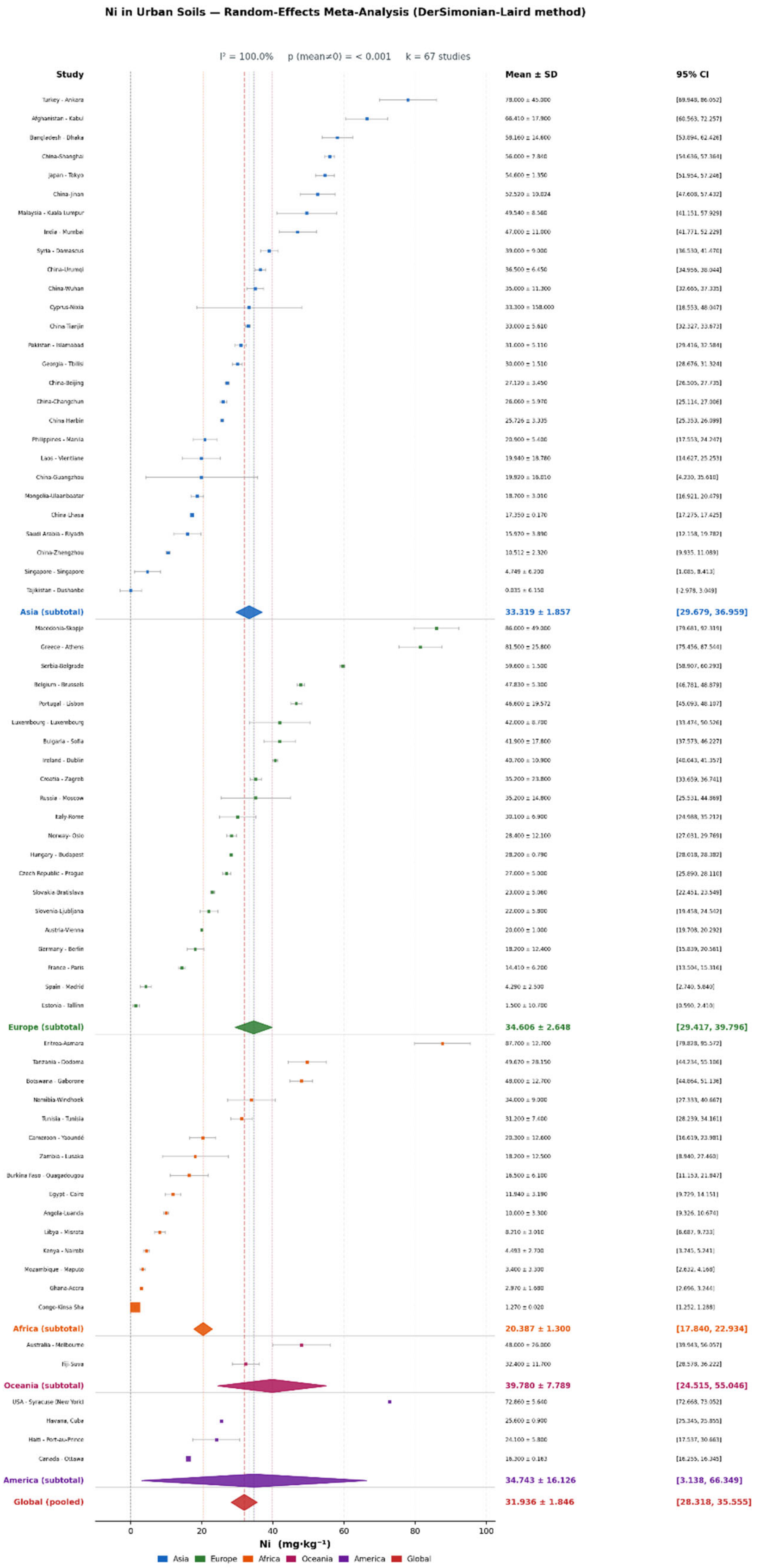

Figure S6. Forest plot of meta-analysis for Ni concentrations in global urban soils.

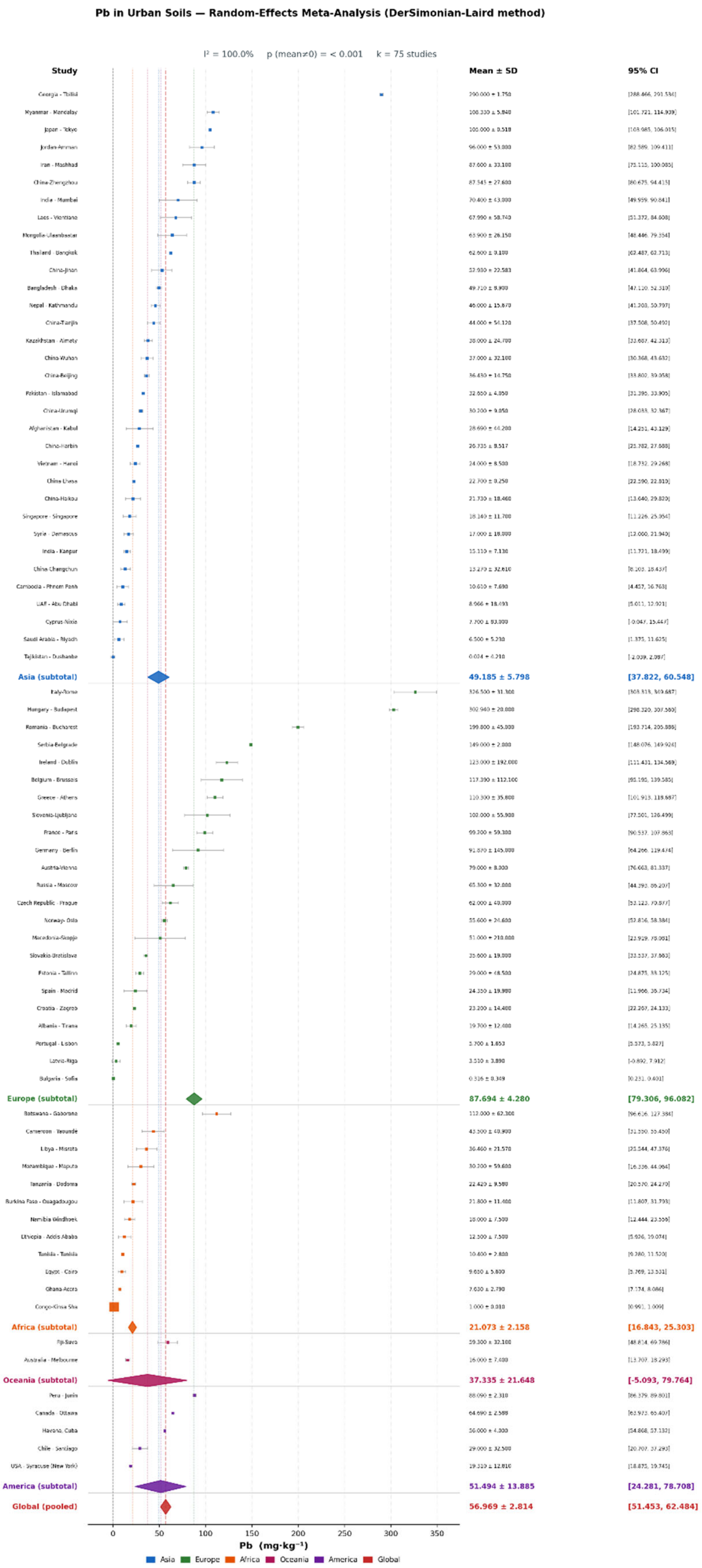

Figure S7. Forest plot of meta-analysis for Pb concentrations in global urban soils.

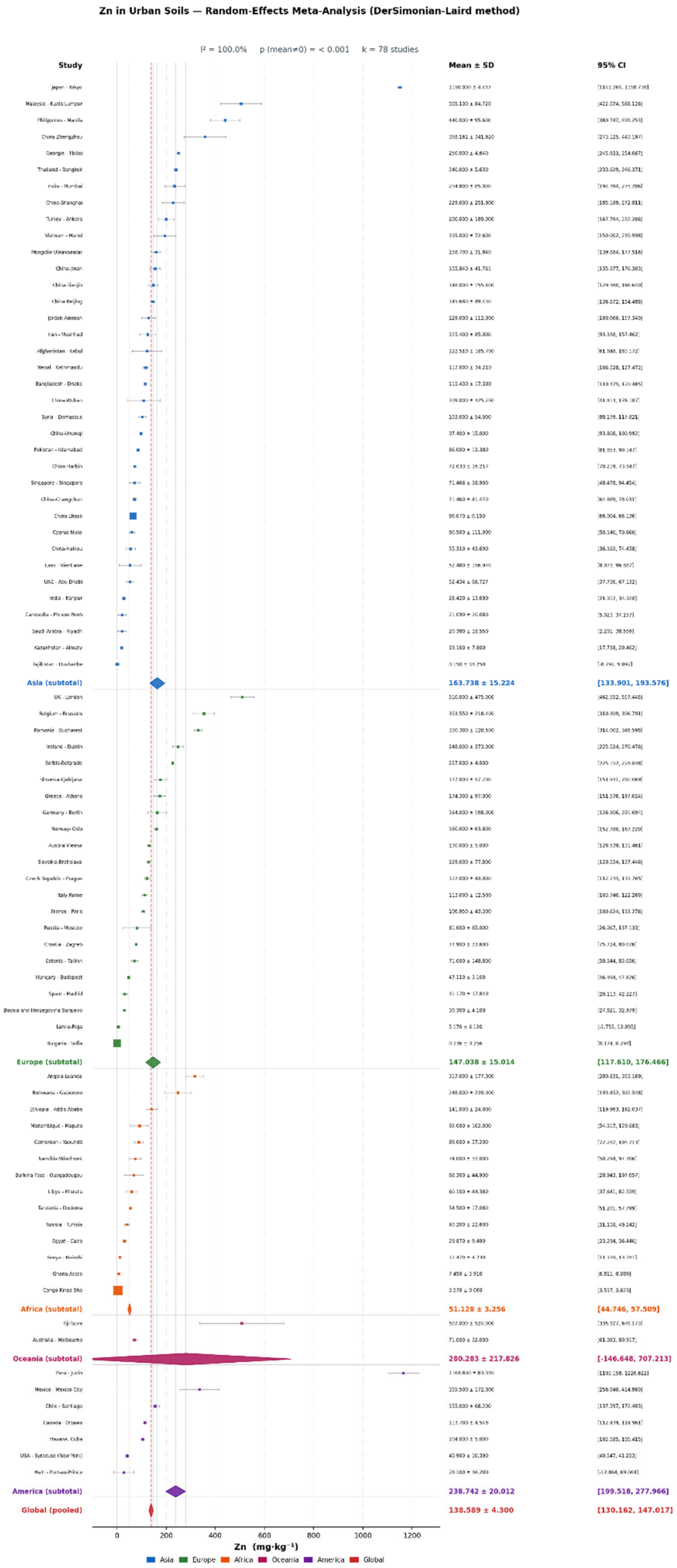

Figure S8. Forest plot of meta-analysis for Zn concentrations in global urban soils.

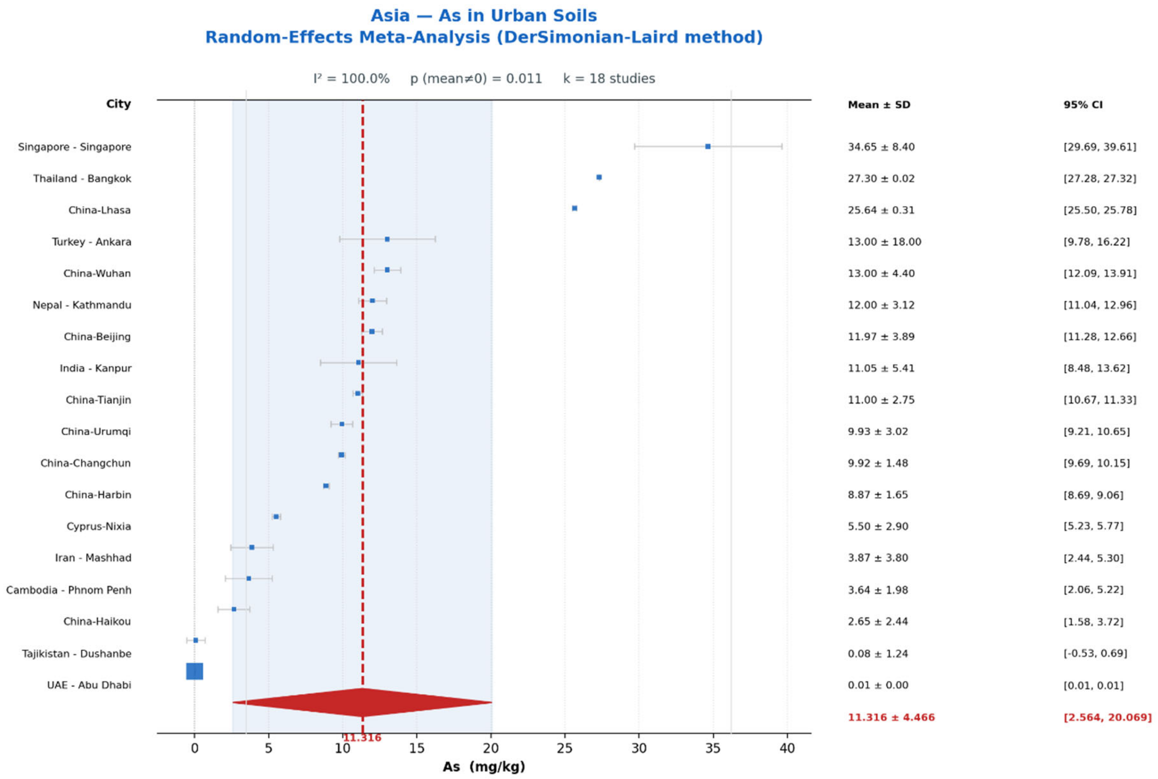

Figure S9. Forest plot of meta-analysis for As concentrations in urban soils of Asia.

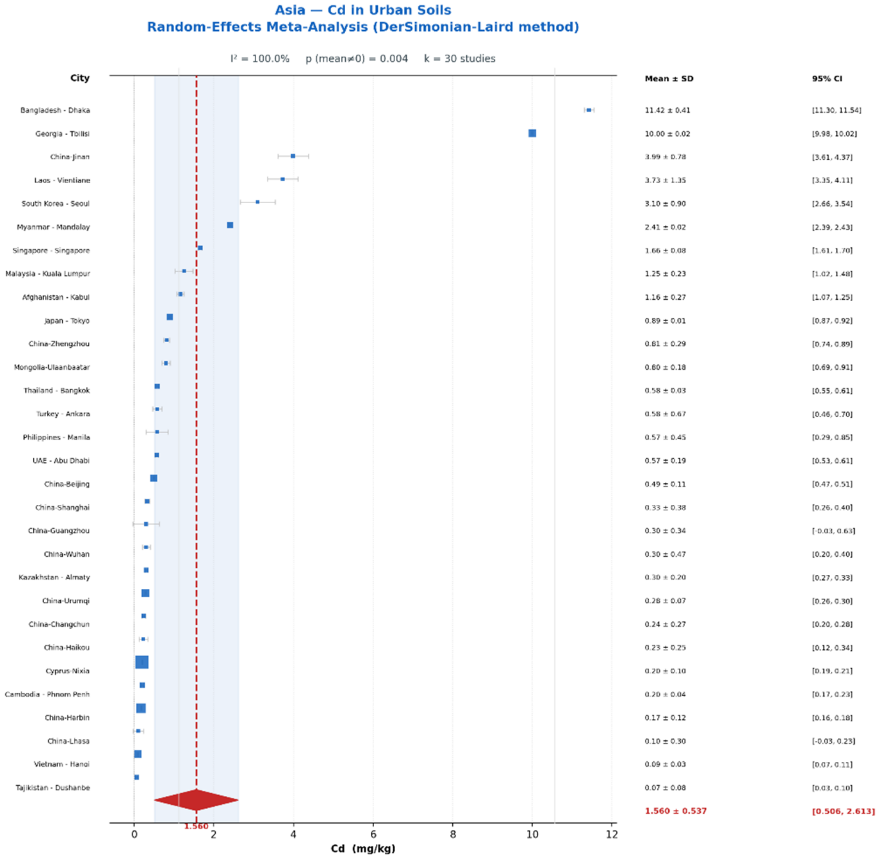

Figure S10. Forest plot of meta-analysis for Cd concentrations in urban soils of Asia.

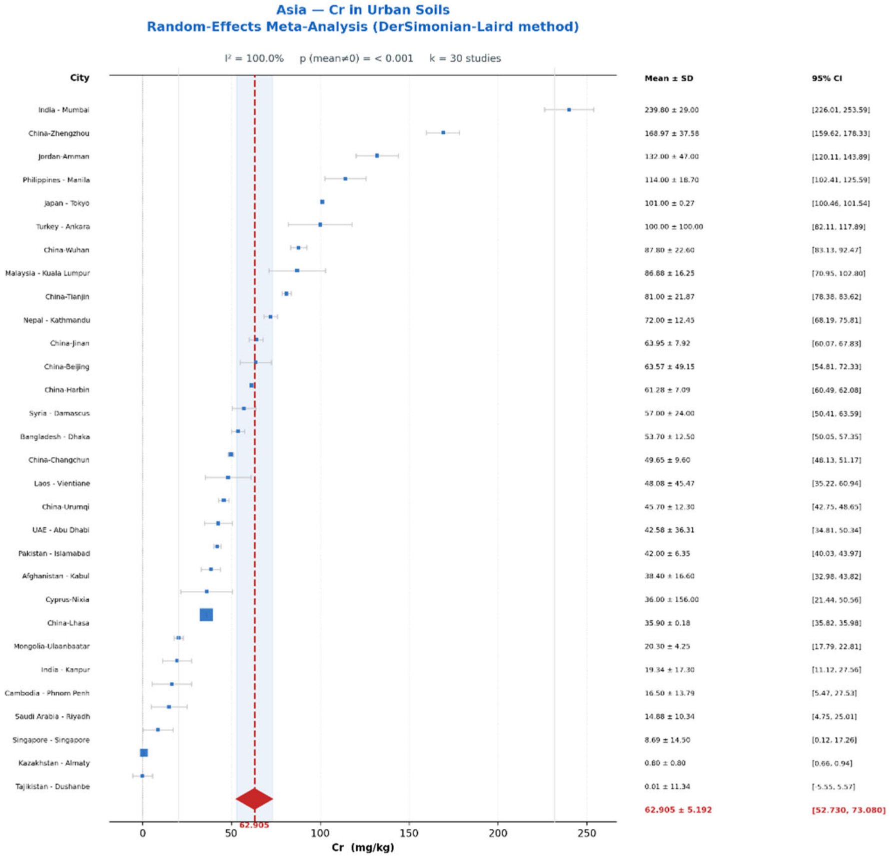

Figure S11. Forest plot of meta-analysis for Cr concentrations in urban soils of Asia.

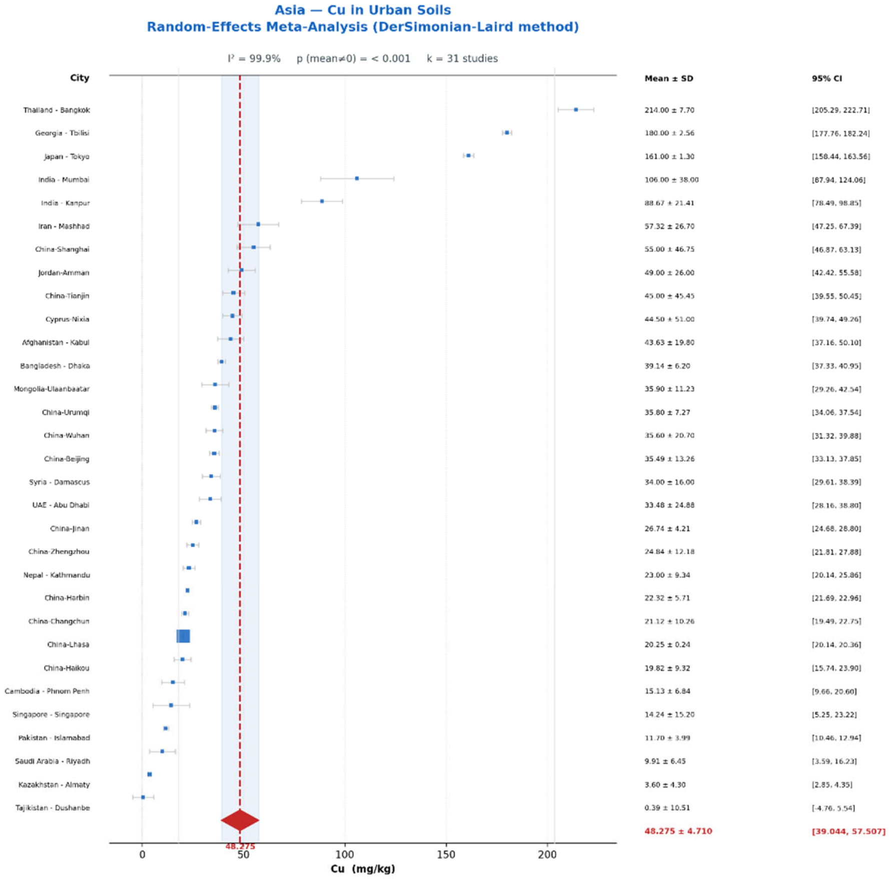

Figure S12. Forest plot of meta-analysis for Cu concentrations in urban soils of Asia.

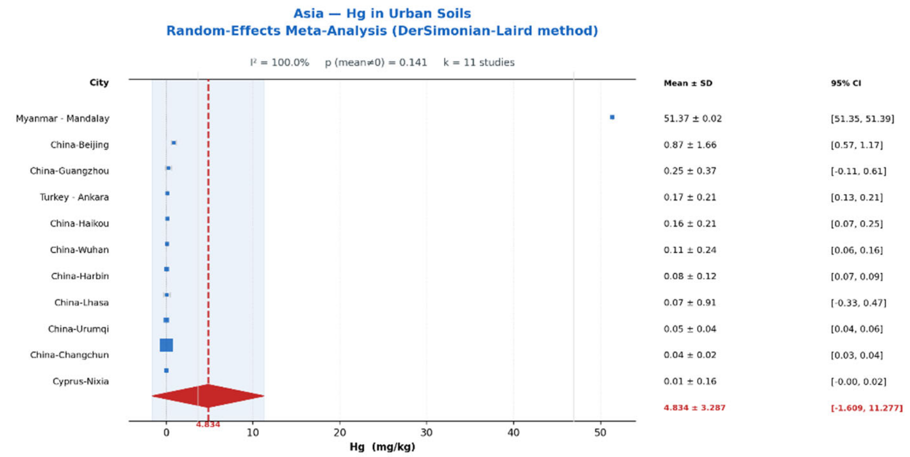

Figure S13. Forest plot of meta-analysis for Hg concentrations in urban soils of Asia.

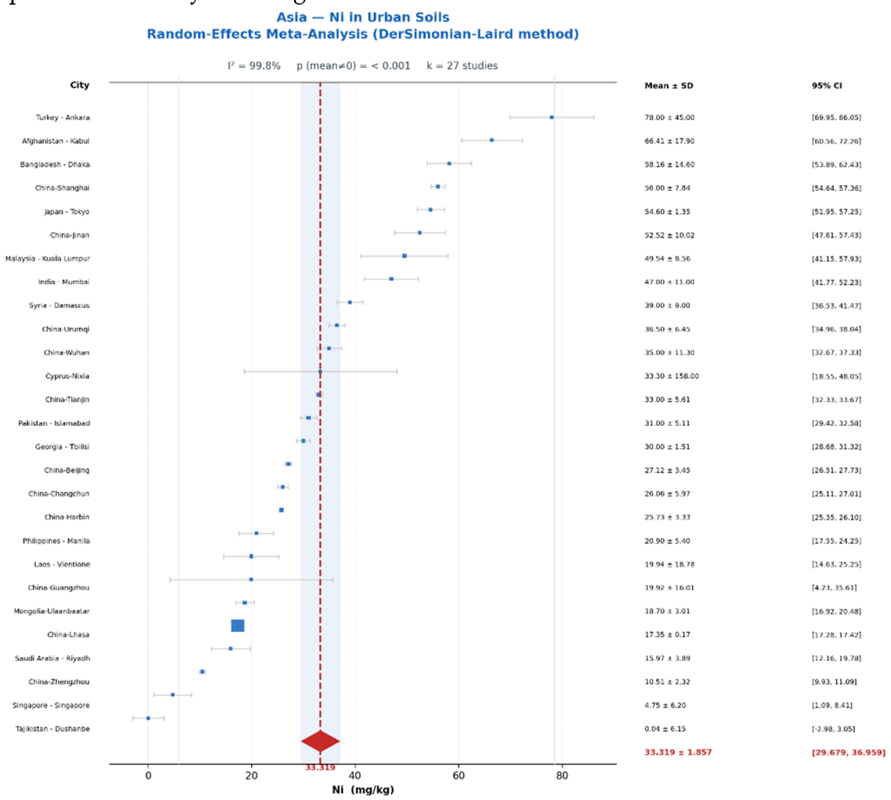

Figure S14. Forest plot of meta-analysis for Ni concentrations in urban soils of Asia.

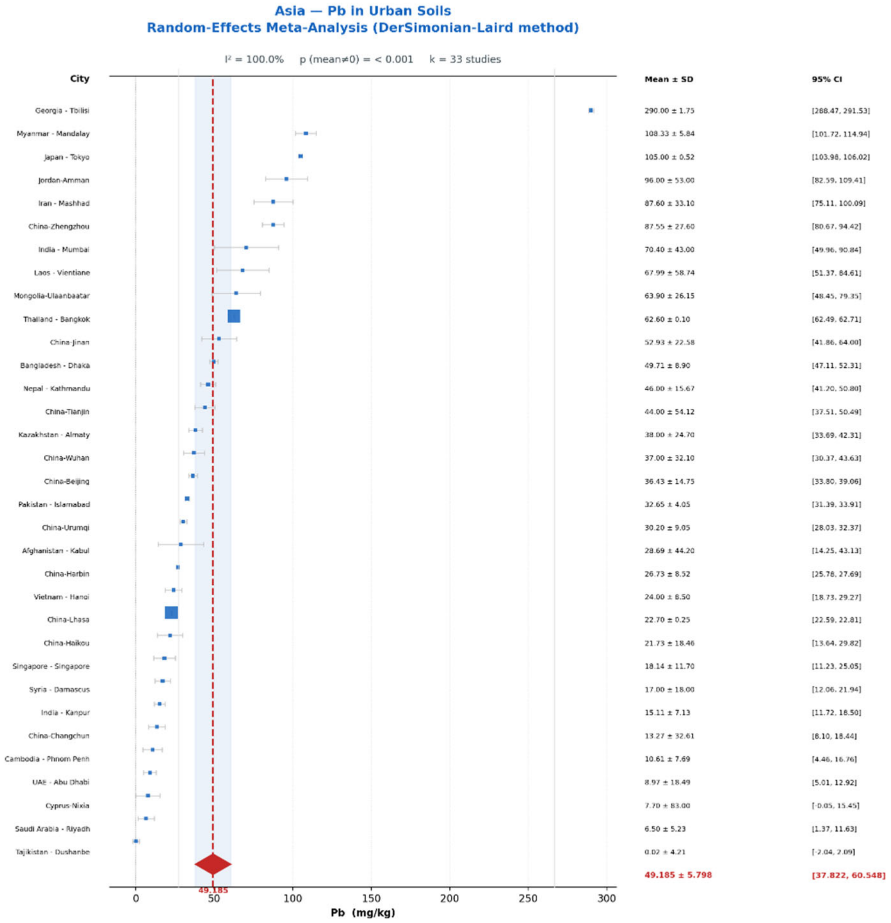

Figure S15. Forest plot of meta-analysis for Pb concentrations in urban soils of Asia.

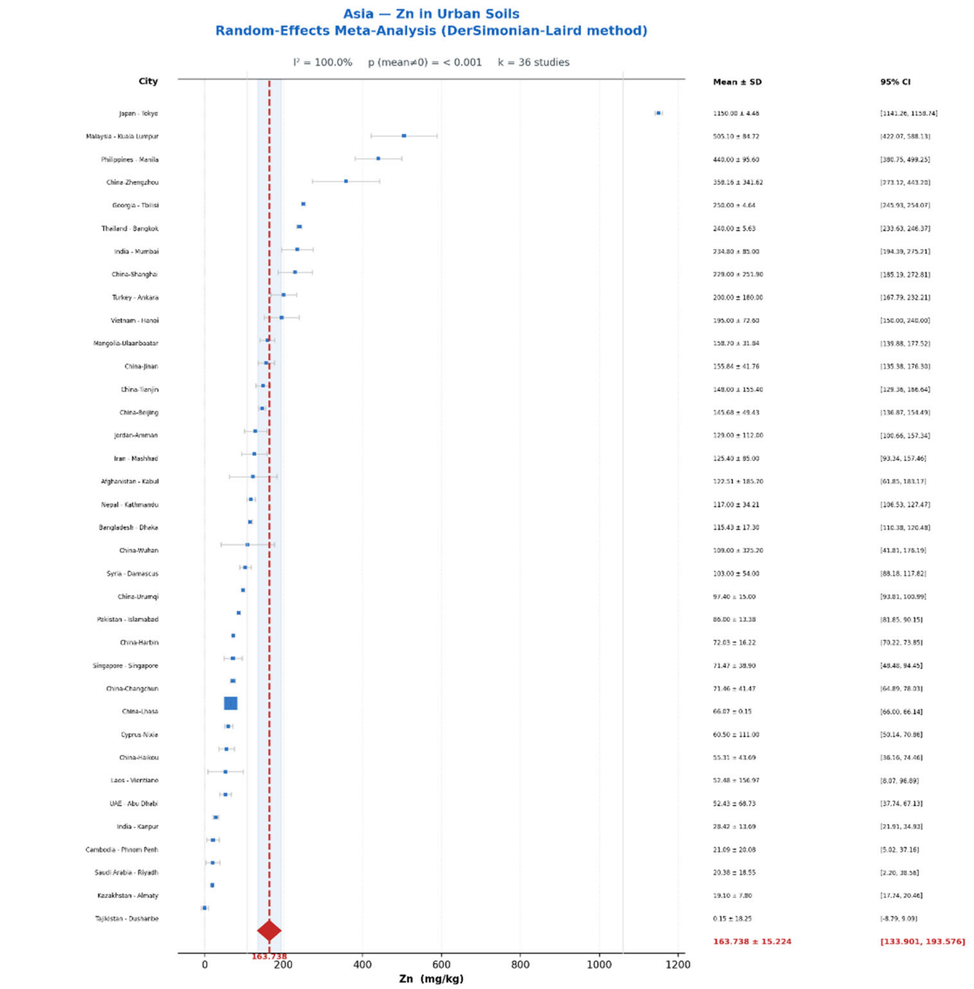

Figure S16. Forest plot of meta-analysis for Zn concentrations in urban soils of Asia.

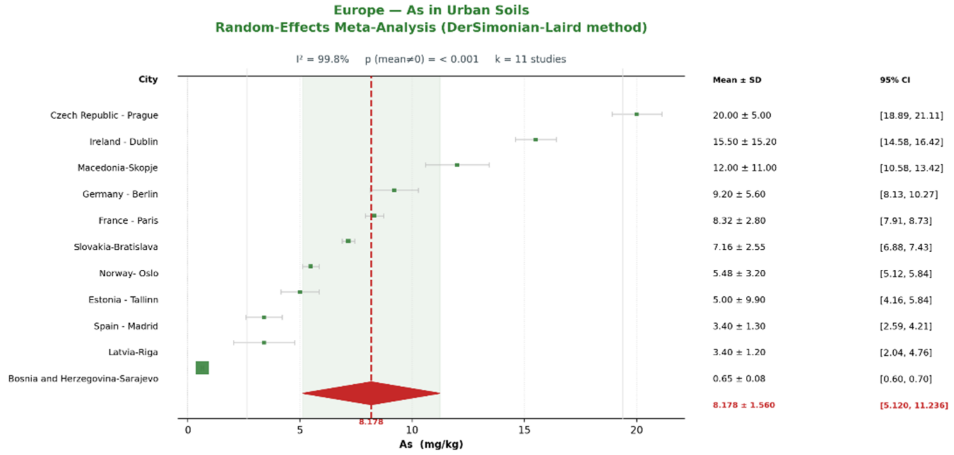

Figure S17. Forest plot of meta-analysis for As concentrations in urban soils of Europe.

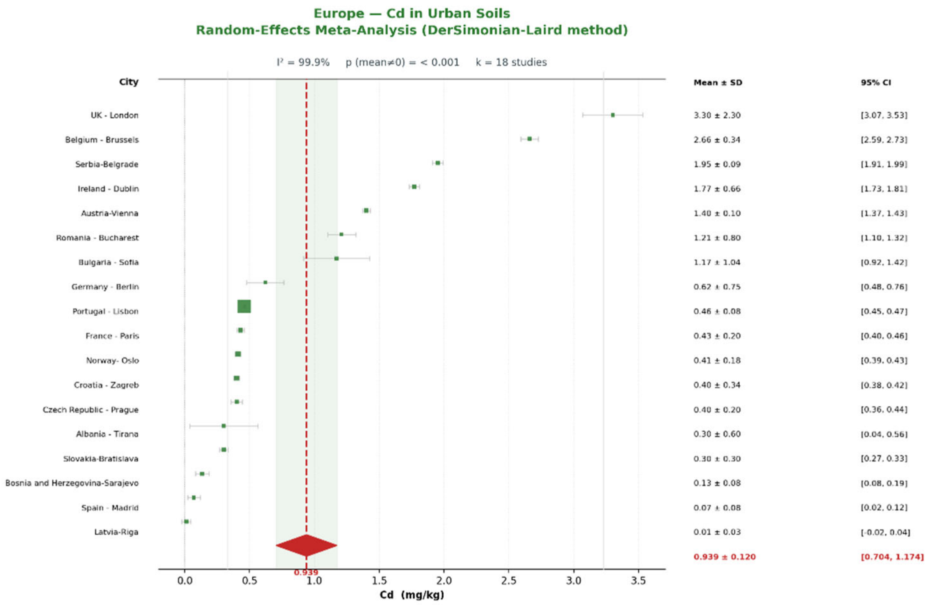

Figure S18. Forest plot of meta-analysis for Cd concentrations in urban soils of Europe.

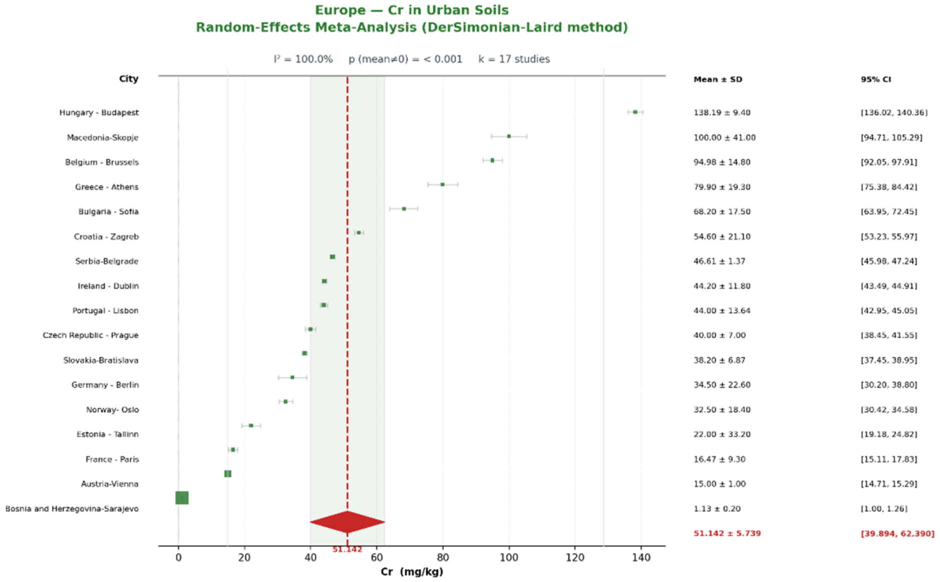

Figure S19. Forest plot of meta-analysis for Cr concentrations in urban soils of Europe.

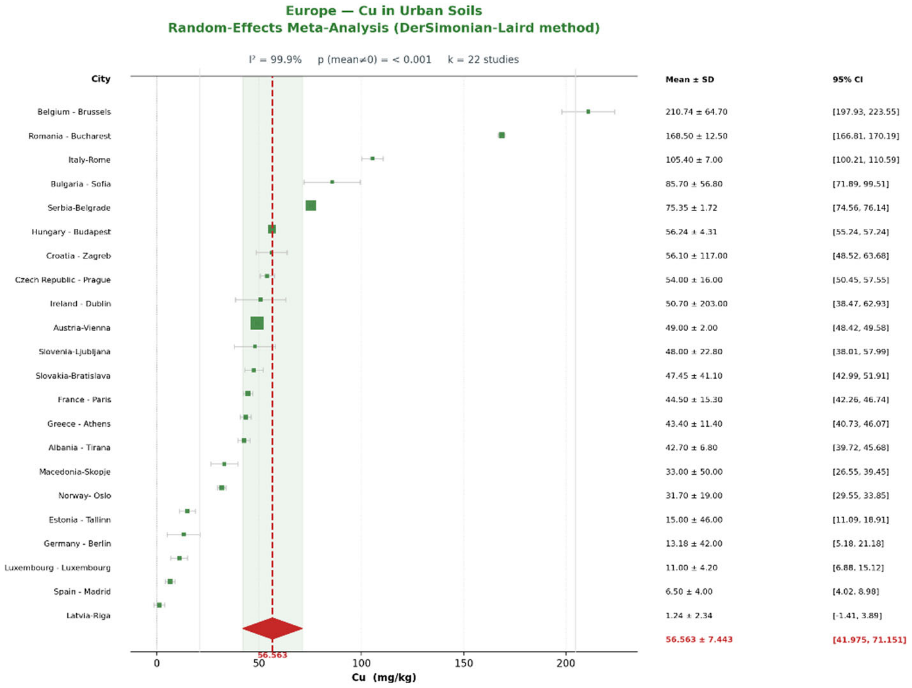

Figure S20. Forest plot of meta-analysis for Cu concentrations in urban soils of Europe.

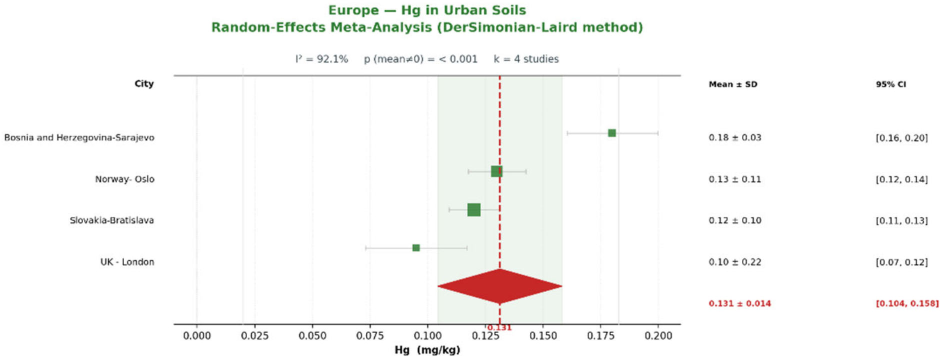

Figure S21. Forest plot of meta-analysis for Hg concentrations in urban soils of Europe.

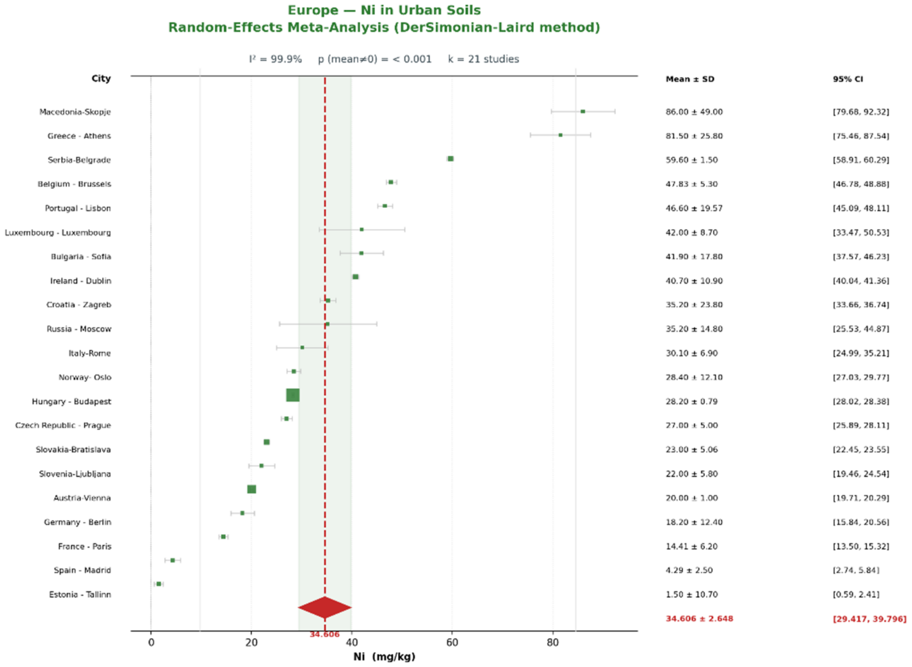

Figure S22. Forest plot of meta-analysis for Ni concentrations in urban soils of Europe.

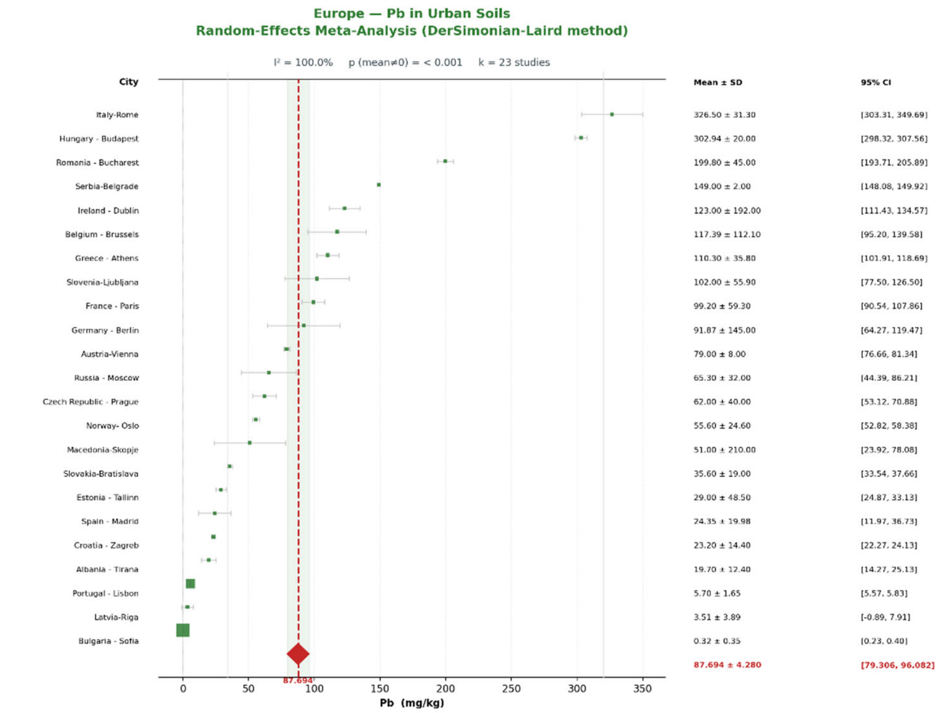

Figure S23. Forest plot of meta-analysis for Pb concentrations in urban soils of Europe.

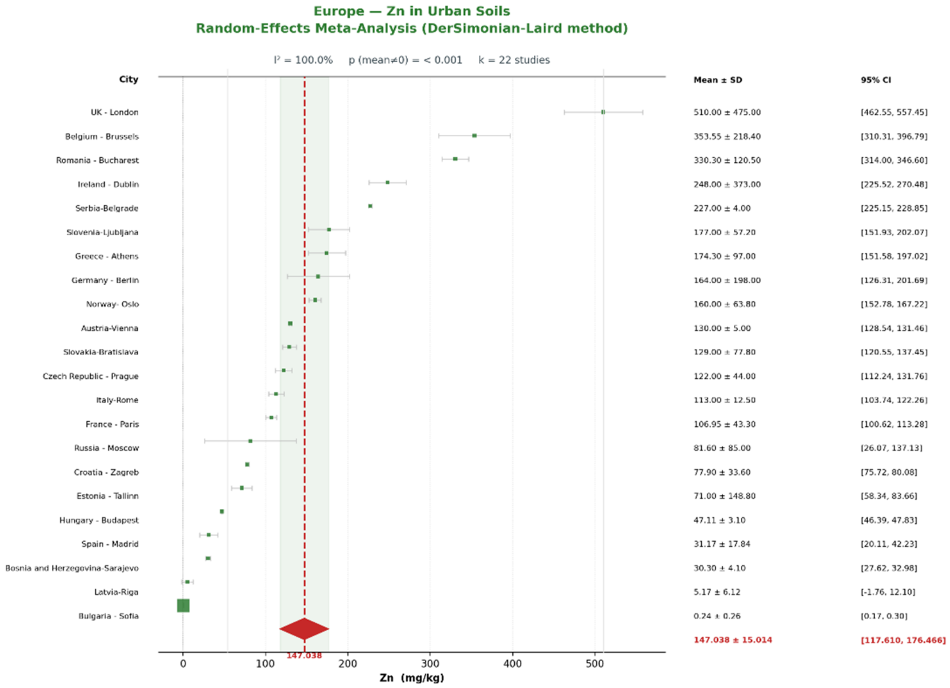

Figure S24. Forest plot of meta-analysis for Zn concentrations in urban soils of Europe.

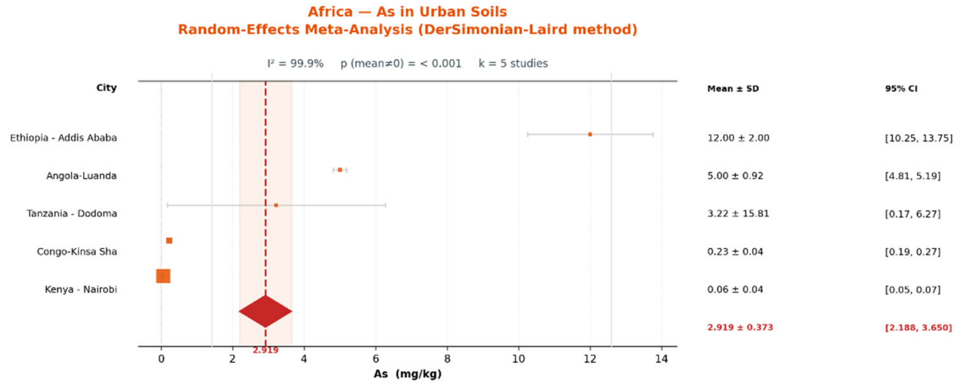

Figure S25. Forest plot of meta-analysis for As concentrations in urban soils of Africa.

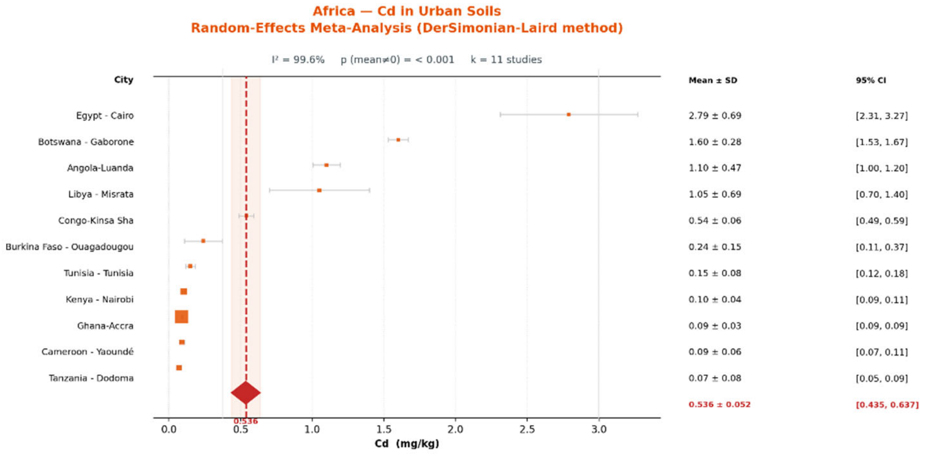

Figure S26. Forest plot of meta-analysis for Cd concentrations in urban soils of Africa.

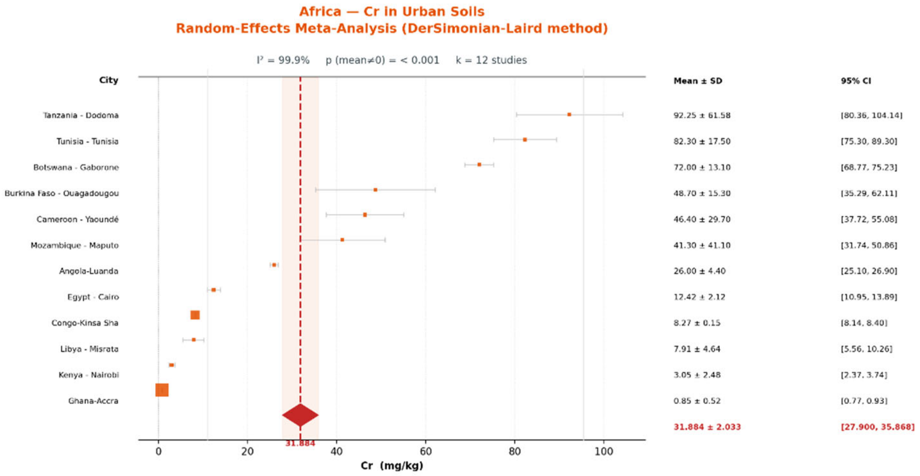

Figure S27. Forest plot of meta-analysis for Cr concentrations in urban soils of Africa.

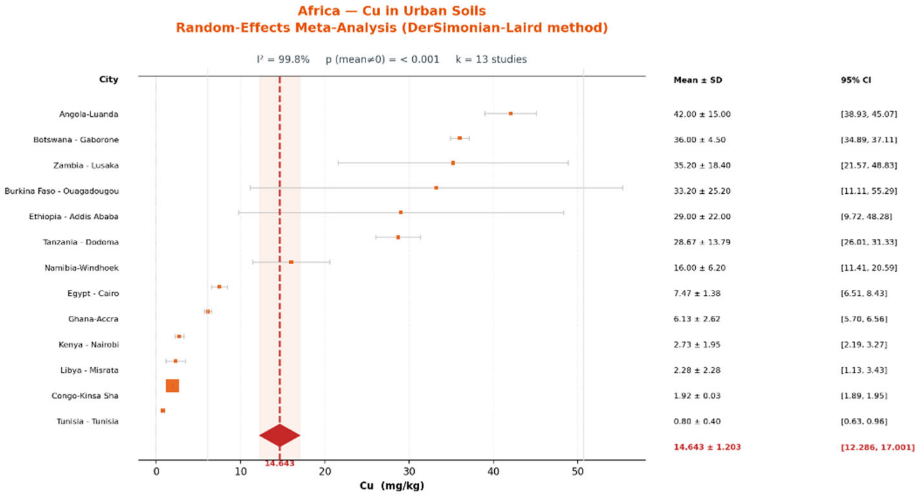

Figure S28. Forest plot of meta-analysis for Cu concentrations in urban soils of Africa.

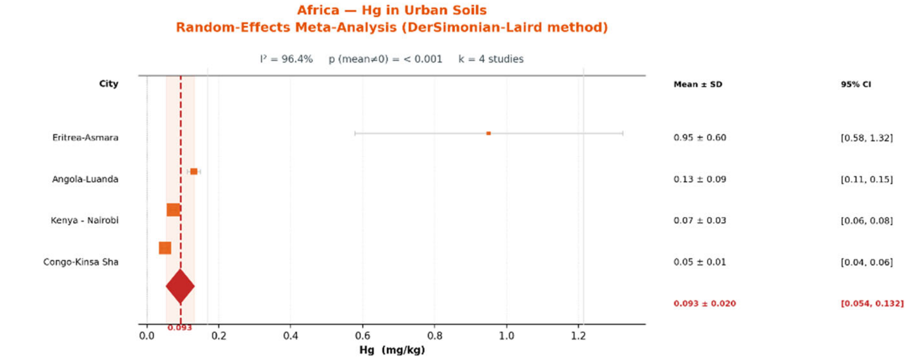

Figure S29. Forest plot of meta-analysis for Hg concentrations in urban soils of Africa.

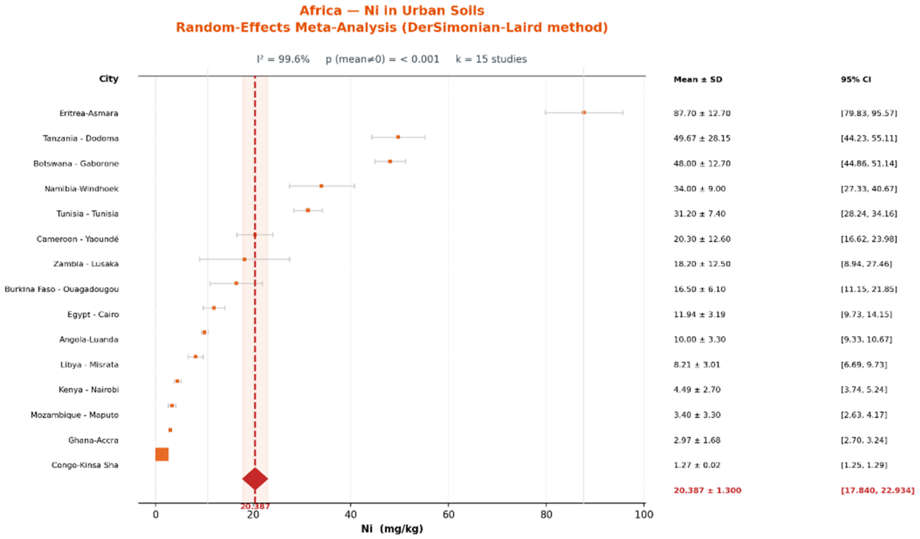

Figure S30. Forest plot of meta-analysis for Ni concentrations in urban soils of Africa.

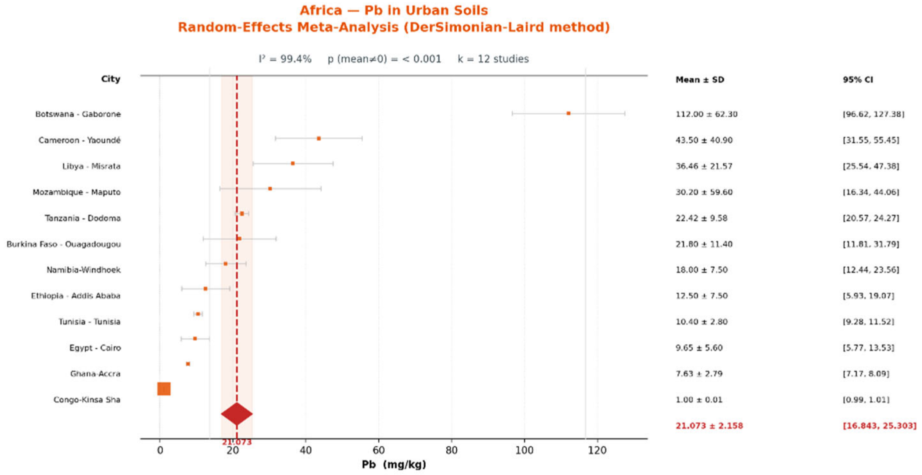

Figure S31. Forest plot of meta-analysis for Pb concentrations in urban soils of Africa.

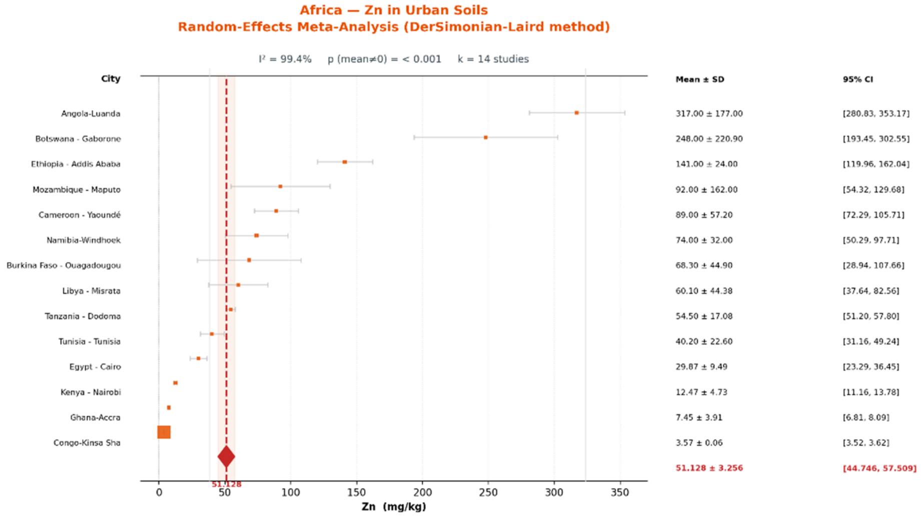

Figure S32. Forest plot of meta-analysis for Zn concentrations in urban soils of Africa.

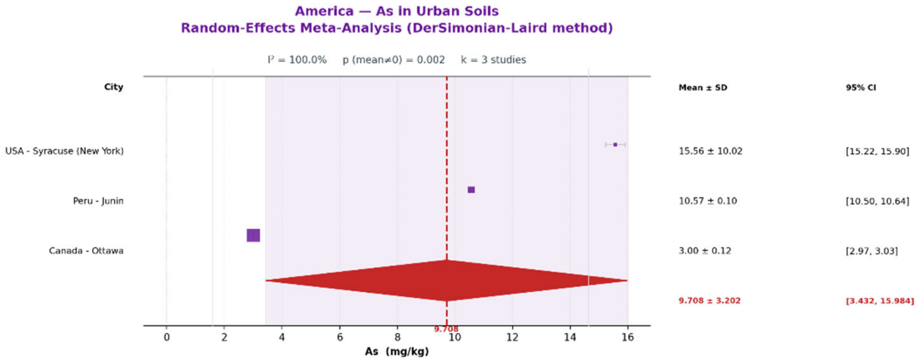

Figure S33. Forest plot of meta-analysis for As concentrations in urban soils of America.

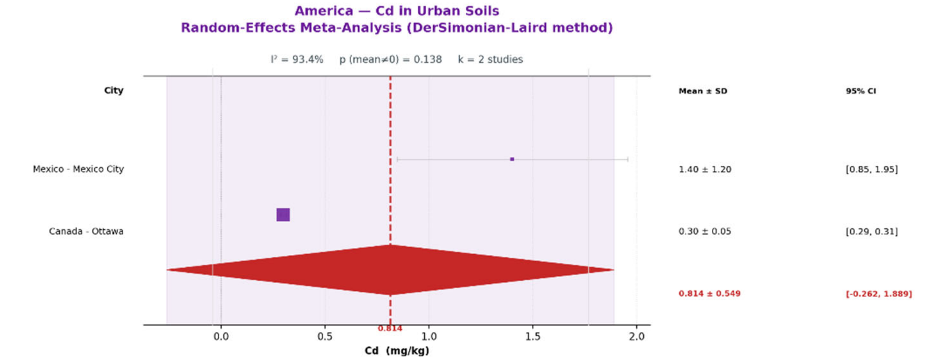

Figure S34. Forest plot of meta-analysis for Cd concentrations in urban soils of America.

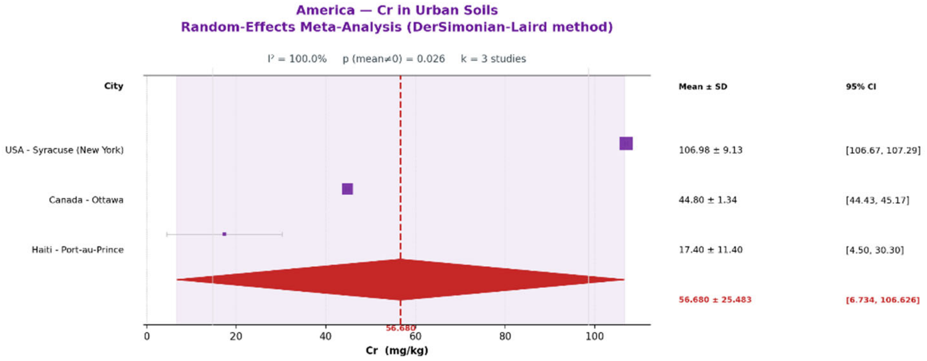

Figure S35. Forest plot of meta-analysis for Cr concentrations in urban soils of America.

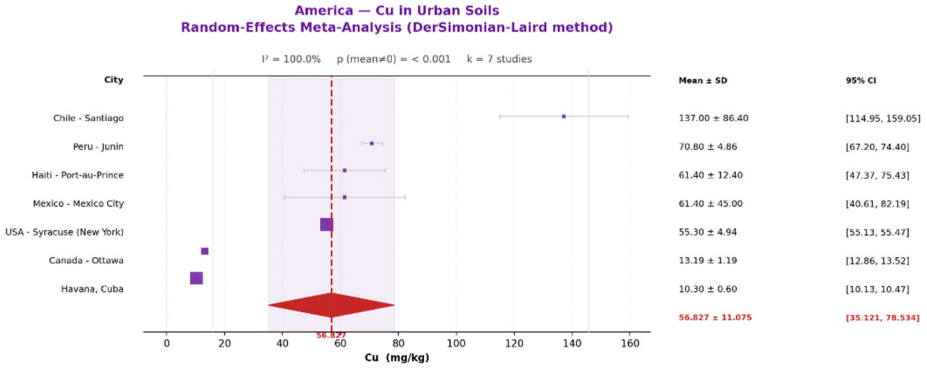

Figure S36. Forest plot of meta-analysis for Cu concentrations in urban soils of America.

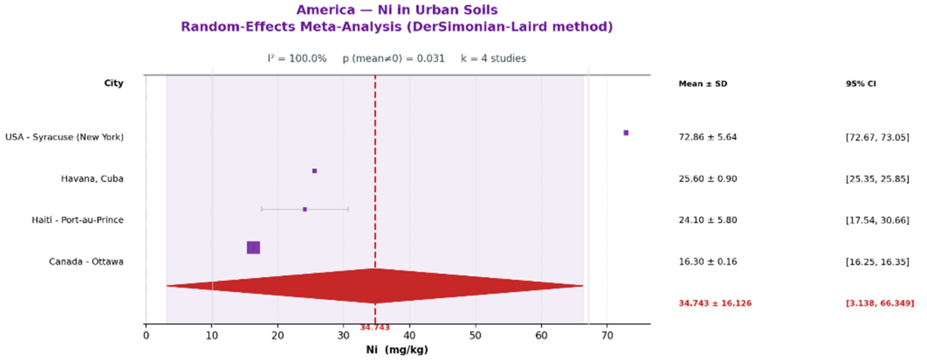

Figure S37. Forest plot of meta-analysis for Ni concentrations in urban soils of America.

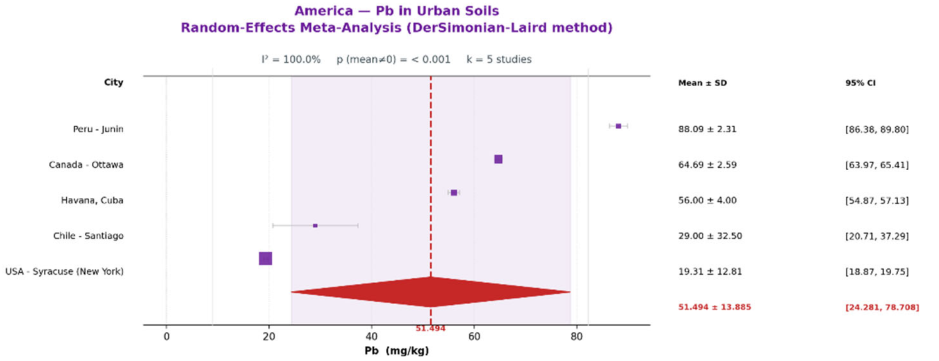

Figure S38. Forest plot of meta-analysis for Pb concentrations in urban soils of America.

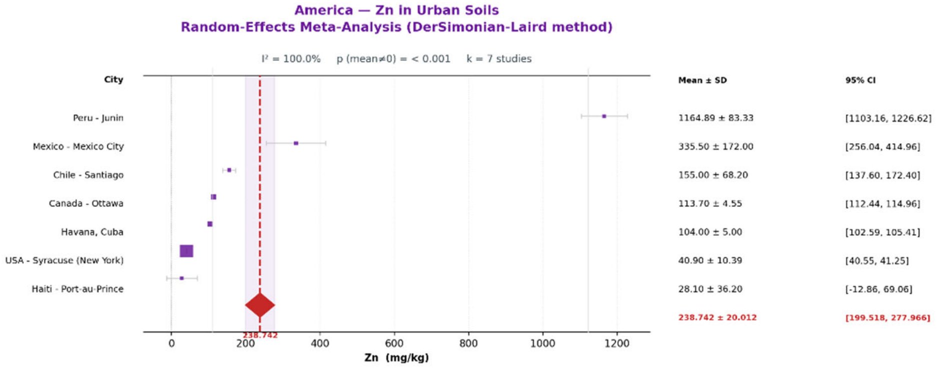

Figure S39. Forest plot of meta-analysis for Zn concentrations in urban soils of America.

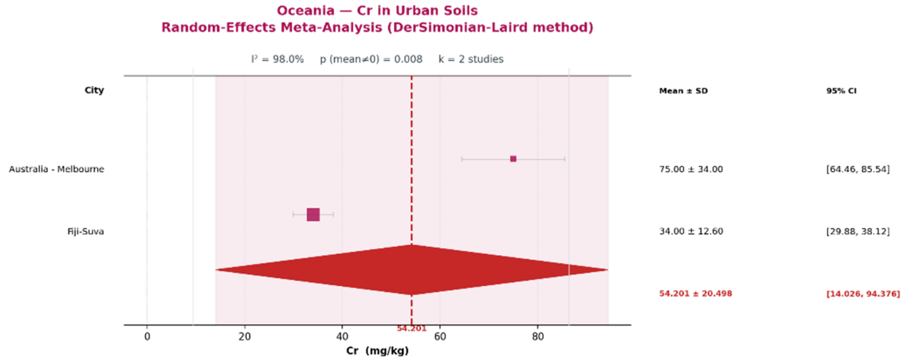

Figure S40. Forest plot of meta-analysis for Cr concentrations in urban soils of Oceania.

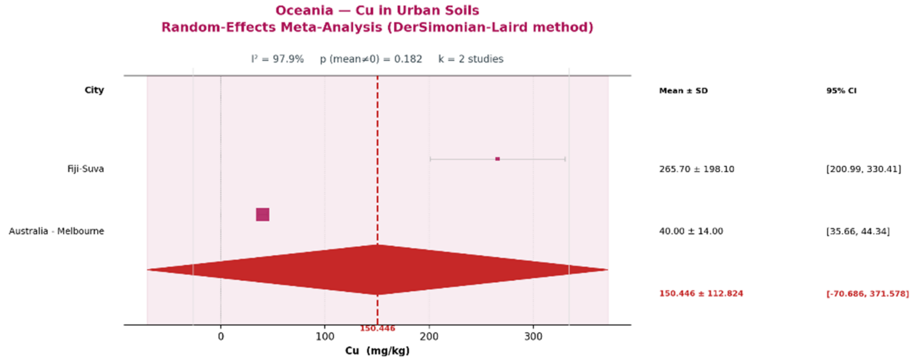

Figure S41. Forest plot of meta-analysis for Cu concentrations in urban soils of Oceania.

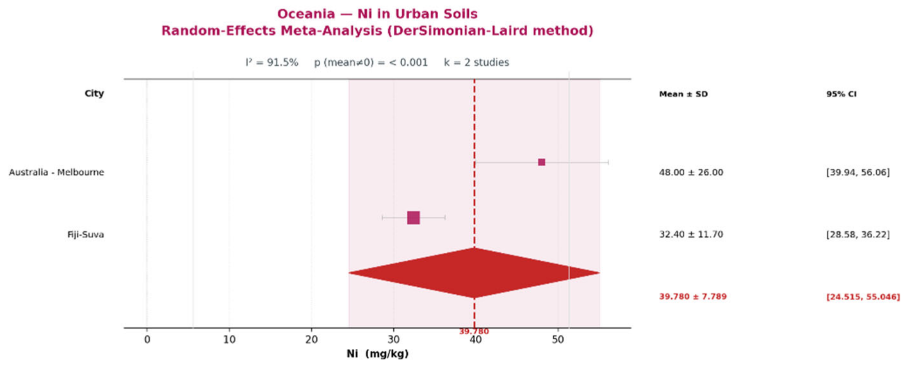

Figure S42. Forest plot of meta-analysis for Ni concentrations in urban soils of Oceania.

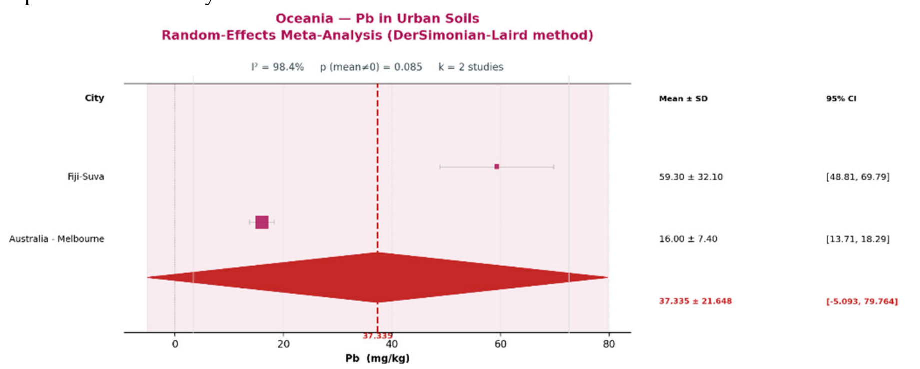

Figure S43. Forest plot of meta-analysis for Pb concentrations in urban soils of Oceania.

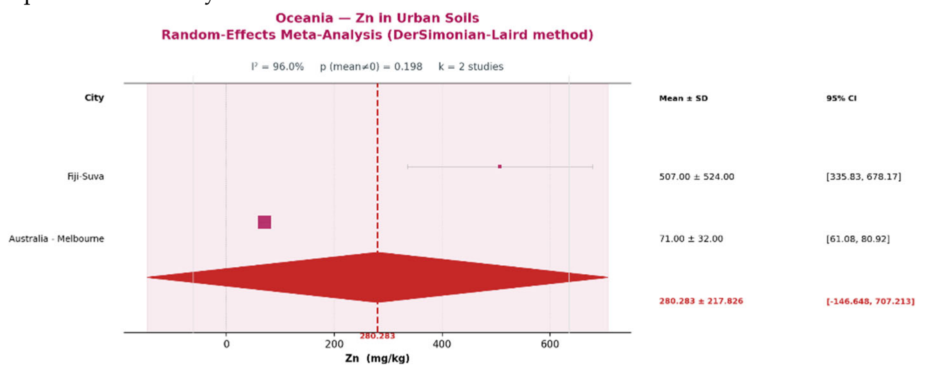

Figure S44. Forest plot of meta-analysis for Zn concentrations in urban soils of Oceania.

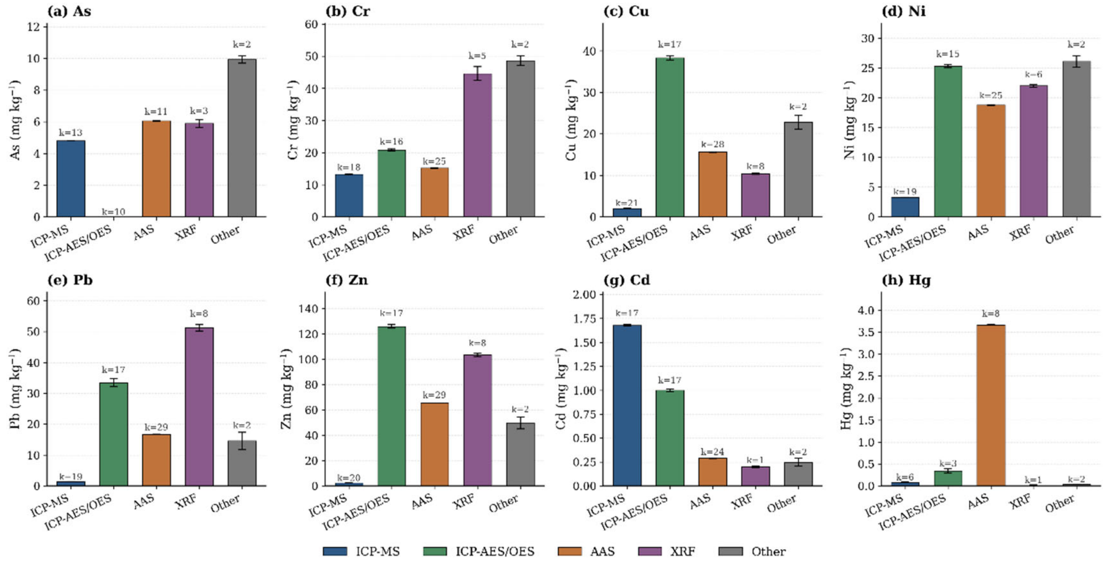

Figure S45. Method-stratified sensitivity of pooled PTE concentrations. Inverse-variance-weighted random-effects pooled means by analytical technique (ICP-MS, ICP-AES/OES, AAS, XRF, Other) for the eight priority PTEs; error bars = 95 % CI; k = number of studies per subgroup. Between-method differences reflect confounding between technique and study site (era, contamination level) rather than systematic analytical bias (see Section 3.4).

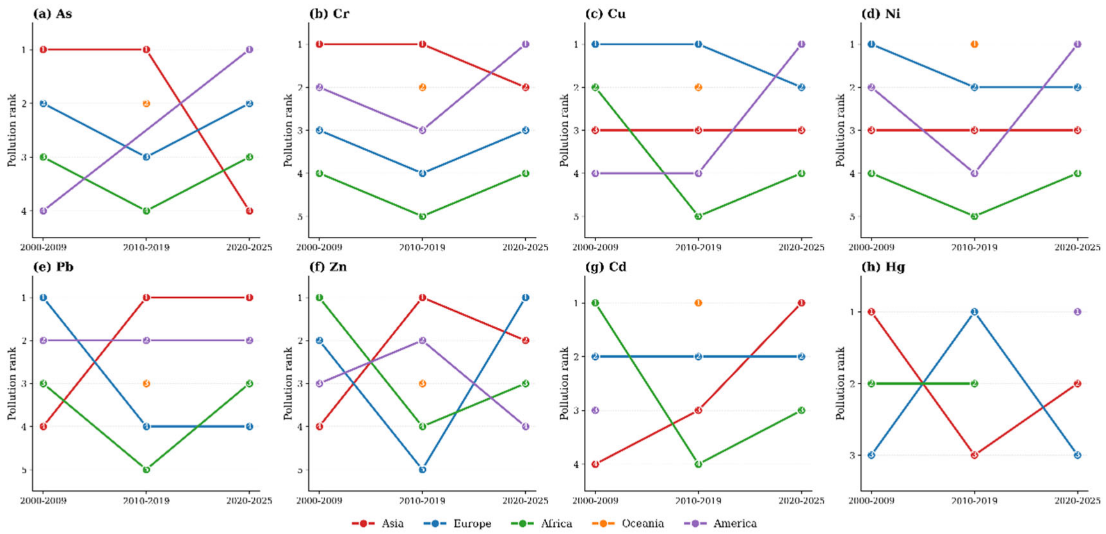

Figure S46. Decadal sensitivity of continental pollution rankings. Continental rankings of inverse-variance-weighted pooled means by sampling decade (2000–2009, 2010–2019, 2020–2025); rank 1 = continent with the highest pooled mean for that element-decade.

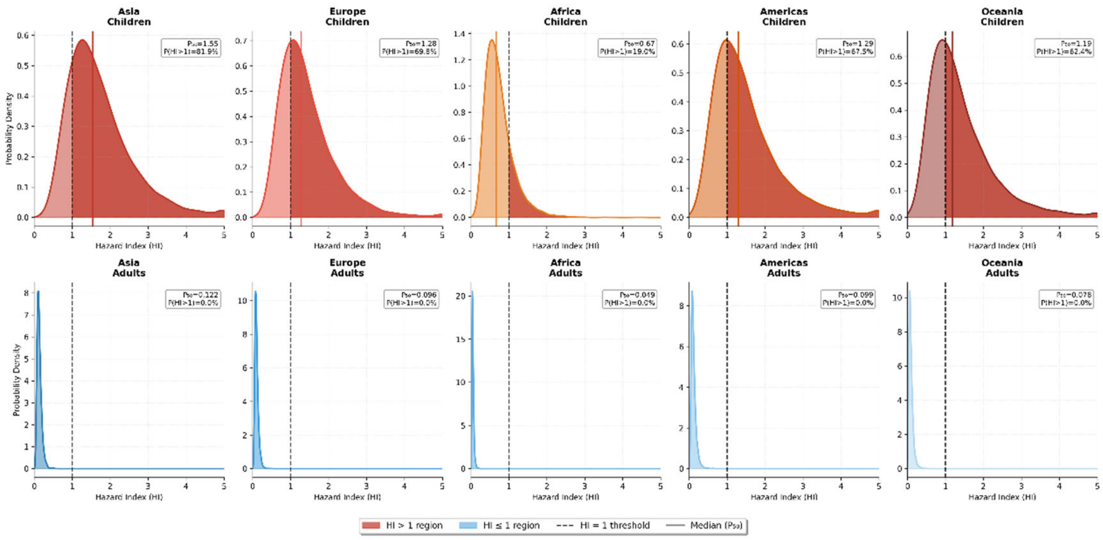

Figure S47. Probability density distributions of the cumulative Hazard Index (*HI*) from Monte Carlo simulation (*N* = 10,000 iterations), stratified by age group and continent. Red-shaded regions indicate *HI* > 1; vertical dashed lines mark the safety threshold (*HI* = 1); solid coloured lines indicate the median (*P*50). Children in Asia, Europe, and the Americas exhibit *P*50 > 1, while all adult subgroups remain well below the threshold. Values correspond to the revised Table A5. Oceania estimates (*k* = 2 underlying studies) should be interpreted as illustrative rather than statistically definitive.

B. Supplementary tables

Table S1. List of Global Core and Capital Cities and Metadata on PTEs Concentrations (mg·kg^{-1}).

Continent	City	As	Cr	Cu	Ni	Pb	Zn	Cd	Hg	Number of samples	Reference
Asia	China-Changchun	9.92	49.65	21.12	26.06	13.27	71.46	0.24	0.036	153	[16]
	China-Harbin	8.872	61.283	22.325	25.726	26.735	72.033	0.17	0.079	307	[17]
	China-Beijing	11.97	63.57	35.49	27.12	36.43	145.68	0.49	0.87	121	[30]
	China-Shanghai	—	128	55	56	119	229	0.33	—	127	[18]
	China-Tianjin	11	81	45	33	44	148	0.39	0.18	267	[19]
	China-Zhengzhou	—	168.972	24.845	10.512	87.545	358.161	0.815	—	62	[20]
	China-Jinan	592.48	63.95	26.74	52.52	52.93	155.84	3.99	3.75	16	[21]
	China-Wuhan	13	87.8	35.6	35	37	109	0.3	0.11	90	[70]
	China-Guangzhou	16.07	62.21	25.42	19.92	57.19	140.21	0.3	0.25	4	[29]
	China-Haikou	2.65	59.25	19.82	53.22	21.73	55.31	0.23	0.16	20	[22]
	China-Urumqi	9.93	45.7	35.8	36.5	30.2	97.4	0.28	0.047	67	[23]
	China-Lhasa	25.64	35.9	20.25	17.35	22.7	66.07	0.1	0.07	20	[24]
	South Korea - Seoul	—	—	84	—	240	271	3.1	—	16	[25]
	Japan - Tokyo	—	101	161	54.6	105	1150	0.892	—	1	[26]
	Mongolia-Ulaanbaatar	14	20.3	35.9	18.7	63.9	158.7	0.8	—	11	[27]
	Vietnam - Hanoi	—	34	22	14	24	195	0.09	—	10	[28]
	Laos - Vientiane	—	48.08	54.06	19.94	67.99	52.48	3.73	—	48	[31]
	Thailand - Bangkok	27.3	—	214	—	62.6	240	0.58	—	3	[32]
	Cambodia - Phnom Penh	3.64	16.5	15.13	—	10.61	21.09	0.2	—	6	[33]
	Myanmar - Mandalay	19.49	—	—	—	108.33	—	2.41	51.37	3	[34]
	Bangladesh - Dhaka	—	53.7	39.14	58.16	49.71	115.43	11.42	—	45	[35]
	Malaysia - Kuala Lumpur	—	86.88	225	49.54	239.3	505.1	1.25	—	4	[36]
	Philippines - Manila	—	114	98.7	20.9	213.6	440	0.57	—	10	[27]
	Singapore - Singapore	34.65	8.69	14.236	4.749	18.14	71.466	1.655	—	11	[37]
	India - Kanpur	11.05	19.34	88.67	80.6	15.11	28.42	2.09	4.33	17	[38]
	India - Mumbai	—	239.8	106	47	70.4	234.8	—	—	17	[39]

Table S1. *Cont.*

Continent	City	As	Cr	Cu	Ni	Pb	Zn	Cd	Hg	Number of samples	Reference
Asia	Nepal - Kathmandu	12	72	23	19	46	117	60	—	41	[40]
	Pakistan - Islamabad	—	42	11.7	31	32.65	86	—	—	40	[41]
	Saudi Arabia - Riyadh	—	14.88	9.91	15.97	6.5	20.38	0.11	—	4	[42]
	UAE - Abu Dhabi	0.007	42.577	33.479	68.795	8.966	52.434	0.568	—	84	[43]
	Georgia - Tbilisi	—	—	180	30	290	250	10	—	5	[44]
	Turkey - Ankara	13	100	250	78	158	200	0.58	0.17	120	[45]
	Syria - Damascus	—	57	34	39	17	103	—	—	51	[46]
	Jordan-Amman	—	132	49	116	96	129	6	—	60	[47]
	Iran - Mashhad	3.87	119.12	57.32	110.2	87.6	125.4	—	—	27	[48]
	Afghanistan - Kabul	—	38.4	43.63	66.41	28.69	122.51	1.16	—	36	[49]
	Cyprus-Nixia	5.5	36	44.5	33.3	7.7	60.5	0.2	0.01	441	[50]
	Tajikistan - Dushanbe	0.08	0.009	0.394	0.035	0.024	0.15	0.065	—	16	[51]
	Kazakhstan - Almaty	—	0.8	3.6	—	38	19.1	0.3	—	126	[71]
Europe	Austria-Vienna	—	15	49	20	79	130	1.4	—	45	[53]
	Albania - Tirana	—	174.2	42.7	305.9	19.7	95.5	0.3	—	20	[54]
	Ireland - Dublin	15.5	44.2	50.7	40.7	123	248	1.77	0.339	1058	[55]
	Estonia - Tallinn	5	22	15	1.5	29	71	—	—	531	[56]
	Bulgaria - Sofia	56	68.2	85.7	41.9	0.316	0.236	1.17	—	65	[57]
	Bosnia and Herzegovina-Sarajevo	0.65	1.13	—	—	—	30.3	0.134	0.18	9	[58]
	Belgium - Brussels	—	94.98	210.74	47.83	117.39	353.55	2.66	—	98	[59]
	Germany - Berlin	9.2	34.5	13.18	18.2	91.87	164	0.62	—	106	[60]
	Russia - Moscow	8.25	62	84.5	35.2	65.3	81.6	0.5	—	9	[61]
	France - Paris	8.32	16.47	44.5	14.41	99.2	106.95	0.43	—	180	[62]
	Czech Republic - Prague	20	40	54	27	62	122	0.4	—	78	[63]
	Croatia - Zagreb	—	54.6	56.1	35.2	23.2	77.9	0.4	—	916	[64]
	Latvia-Riga	3.4	—	1.24	—	3.51	5.17	0.01	—	3	[65]
	Romania - Bucharest	—	—	168.5	—	199.8	330.3	1.21	—	210	[68]

Table S1. *Cont.*

Continent	City	As	Cr	Cu	Ni	Pb	Zn	Cd	Hg	Number of samples	Reference
Europe	Macedonia-Skopje	12	100	33	86	51	100	0.54	—	231	[69]
	Luxembourg - Luxembourg	18.3	110	11	42	—	—	—	—	4	[66]
	Norway- Oslo	5.48	32.5	31.7	28.4	55.6	160	0.41	0.13	300	[67]
	Serbia-Belgrade	—	46.61	75.35	59.6	149	227	1.95	—	18	[52]
	Portugal - Lisbon	—	44	—	46.6	5.7	—	0.46	—	648	[72]
	Slovakia-Bratislava	7.155	38.2	47.45	23	35.6	129	0.3	0.12	326	[73]
	Slovenia-Ljubljana	—	43	48	22	102	177	—	—	20	[74]
	Spain - Madrid	3.4	6.2	6.5	4.29	24.35	31.17	0.07	—	10	[75]
	Greece - Athens	—	79.9	43.4	81.5	110.3	174.3	—	—	70	[76]
	Hungary - Budapest	—	138.19	56.24	28.2	302.94	47.11	—	—	72	[77]
	Italy-Rome	—	—	105.4	30.1	326.5	113	—	—	7	[78]
	UK - London	—	—	131	—	660	510	3.3	0.095	385	[79]
Africa	Ethiopia - Addis Ababa	12	159.5	29	56.5	12.5	141	0.05	—	5	[80]
	Egypt - Cairo	—	12.42	7.47	11.94	9.65	29.87	2.79	—	8	[81]
	Angola-Luanda	5	26	42	10	351	317	1.1	0.13	92	[82]
	Botswana - Gaborone	—	72	36	48	112	248	1.6	—	63	[83]
	Burkina Faso - Ouagadougou	—	48.7	33.2	16.5	21.8	68.3	0.24	—	5	[84]
	Eritrea-Asmara	—	186.4	913.4	87.7	597.5	1144	3.3	0.95	10	[85]
	Congo-Kinsa Sha	0.23	8.27	1.92	1.27	1	3.57	0.54	0.05	5	[86]
	Ghana-Accra	—	0.85	6.13	2.97	7.63	7.45	0.09	—	144	[87]
	Cameroon - Yaoundé	—	46.4	156	20.3	43.5	89	0.09	—	45	[88]
	Kenya - Nairobi	0.058	3.054	2.734	4.493	—	12.47	0.103	0.073	50	[89]
	Libya - Misrata	—	7.91	2.28	8.21	36.46	60.1	1.05	—	15	[90]
	Mozambique - Maputo	—	41.3	59.7	3.4	30.2	92	—	—	71	[91]
	Namibia-Windhoek	—	—	16	34	18	74	—	—	7	[92]
	Tanzania - Dodoma	3.22	92.25	28.67	49.67	22.42	54.5	0.07	—	103	[93]
	Tunisia - Tunisia	—	82.3	0.795	31.2	10.4	40.2	0.15	—	24	[94]
	Zambia - Lusaka	—	39	35.2	18.2	1435	388	6.4	—	7	[95]

Table S1. *Cont.*

Continent	City	As	Cr	Cu	Ni	Pb	Zn	Cd	Hg	Number of samples	Reference
Oceania	Australia - Melbourne	8	75	40	48	16	71	—	—	40	[96]
	Australia - Sydney	—	37	98	10	644	375	2	—	9	[97]
	Fiji-Suva	—	34	265.7	32.4	59.3	507	3.1	—	36	[98]
America	Mexico - Mexico City	—	—	61.4	—	354.1	335.5	1.4	—	18	[99]
	USA - Syracuse (New York)	15.56	106.98	55.3	72.86	19.31	40.9	—	10.46	3324	[100]
	Canada - Ottawa	3	44.8	13.19	16.3	64.69	113.7	0.3	0.107	50	[101]
	Havana, Cuba	—	—	10.3	25.6	56	104	—	—	48	[102]
	Haiti - Port-au-Prince	—	17.4	61.4	24.1	—	28.1	—	—	3	[103]
	Chile - Santiago	—	—	137	—	29	155	—	—	59	[104]
	Peru - Junin	10.57	—	70.8	—	88.09	1164.89	—	—	7	[105]

— indicates there is no relevant data.

Table S2. Geo-accumulation Index (I_{geo}) and Nemerow Composite Pollution Index (P_N) of PTEs in Urban Soils.

City	**As** (I_{geo})	**Cr** (I_{geo})	**Cu** (I_{geo})	**Ni** (I_{geo})	**Pb** (I_{geo})	**Zn** (I_{geo})	**Cd** (I_{geo})	**Hg** (I_{geo})	P_N
China-Changchun	-0.76	-0.50	-0.28	-0.30	-1.70	-0.76	0.69	-1.44	6.40
China-Harbin	-0.92	-0.58	-0.60	-0.65	-0.54	-0.63	0.22	-0.30	8.44
China-Beijing	-0.49	-0.61	0.01	-0.79	0.19	0.36	1.29	3.16	2.93
China-Shanghai	—	0.48	0.70	0.47	1.61	1.04	1.18	—	56.08
China-Tianjin	-0.61	-0.18	0.41	-0.29	0.17	0.41	1.42	0.88	4.34
China-Zhengzhou	—	0.88	-0.45	-1.94	1.17	1.69	2.49	—	1.66
China-Jinan	5.14	-0.52	-0.34	0.38	0.44	0.49	4.78	5.27	2.12
China-Wuhan	-0.37	-0.06	0.07	-0.21	-0.08	-0.03	1.04	0.17	1.69
China-Guangzhou	-0.06	-0.56	-0.42	-1.02	0.55	0.33	1.04	1.36	0.81
China-Haikou	-2.66	-0.63	-0.77	0.40	-0.84	-1.01	0.66	0.71	0.98
China-Urumqi	-0.76	-1.00	0.08	-0.14	-0.37	-0.19	0.94	-1.05	30.59
China-Lhasa	0.61	-1.35	-0.74	-1.22	-0.78	-0.75	-0.54	-0.48	2.06
South Korea - Seoul	—	—	-0.84	—	0.68	-0.73	-0.54	—	0.98
Japan - Tokyo	—	0.27	1.84	0.60	1.81	3.12	1.14	—	2.10
Mongolia-Ulaanbaatar	0.64	-3.47	-2.06	-3.59	-1.23	-1.50	-2.49	—	6.61
Vietnam - Hanoi	—	-0.82	-1.03	-4.10	1.68	2.12	-0.74	—	6.90
Laos - Vientiane	—	-0.32	0.26	-3.59	3.18	0.22	4.64	—	0.55
Thailand - Bangkok	3.60	—	3.88	—	1.21	3.02	1.47	—	0.08
Cambodia - Phnom Penh	0.69	-1.86	2.13	—	-1.27	1.97	3.74	—	1.48
Myanmar - Mandalay	-1.21	—	—	—	-1.05	—	-1.64	4.10	0.02
Bangladesh - Dhaka	—	0.25	0.06	-2.04	-0.59	0.62	6.99	—	112.19
Malaysia - Kuala Lumpur	—	-0.05	2.91	0.72	2.19	2.75	1.47	—	22.53
Philippines - Manila	—	-1.74	-0.79	-0.52	2.43	1.16	0.34	—	8.35
Singapore - Singapore	-0.79	-2.79	-3.40	-5.66	-4.63	-2.65	0.14	—	1.27
India - Kanpur	0.56	-1.96	0.98	-1.57	-0.75	-1.89	2.22	3.27	0.02
India - Mumbai	—	2.19	1.50	-2.35	1.47	1.16	—	—	0.37
Nepal - Kathmandu	3	0.46	-0.38	-0.66	0.62	0.14	7.06	—	1.37
Pakistan - Islamabad	—	-0.51	-1.36	-1.51	0.12	-0.53	—	—	0.71
Saudi Arabia - Riyadh	—	-3.33	-3.92	-2.23	-4.53	-4.46	-5.35	—	12.45
UAE - Abu Dhabi	-8.33	—	0.16	-0.71	-1.74	-1.02	-1.99	—	3.66
Georgia - Tbilisi	—	—	2.58	-3	0.95	—	2.15	—	1.85

Table S2. *Cont.*

City	As (I_{geo})	Cr (I_{geo})	Cu (I_{geo})	Ni (I_{geo})	Pb (I_{geo})	Zn (I_{geo})	Cd (I_{geo})	Hg (I_{geo})	P_N
Turkey - Ankara	2.53	-0.07	3.06	-1.62	1.59	1.15	-7.28	-1.40	0.14
Syria - Damascus	—	-0.73	-0.78	-2.62	-1.78	-0.54	—	—	3.69
Jordan-Amman	—	0.09	0.93	1.19	—	-0.10	—	—	1.73
Iran - Mashhad	-0.73	-1.04	-0.87	-0.92	-2.36	-0.80	—	—	52.50
Afghanistan - Kabul	—	-1.81	-0.63	-0.18	-0.06	—	1.37	—	0.84
Cyprus-Nixia	-0.58	-0.58	-0.59	-2.85	-0.58	-0.58	—	-5.49	1.85
Tajikistan - Dushanbe	-4.81	-12.70	-7.21	-12.74	-11.10	—	-2.79	—	0.50
Kazakhstan - Almaty	—	-3.49	-0.32	—	-0.34	-0.85	-1.32	—	1.15
Mexico - Mexico City	—	—	0.71	—	5.67	1.43	-0.15	—	8.14
USA - Syracuse (New York)	1.79	4.32	3.26	3.17	3.59	5.27	—	—	0.41
Canada - Ottawa	0.95	—	3.03	—	4.82	6.08	-2.47	-4.77	1.06
Havana, Cuba	—	—	-0.44	-0.44	0.78	-0.17	—	—	2.08
Chile - Santiago	—	—	0.54	—	-2.86	-1.27	—	—	5.93
Haiti - Port-au-Prince	—	-1.37	-0.58	-1.85	—	-3.01	—	—	3.76
Peru - Junin	-2.83	—	-1.08	—	-0.25	-3.27	—	—	2.33
Austria-Vienna	-4.91	-7.05	—	—	—	-3.89	-4.07	—	1.21
Albania - Tirana	-8.23	-1.65	-2.52	-1.52	-3.26	-2.44	-3.17	—	6.26
Ireland - Dublin	1.88	—	2.11	—	2.37	4.19	-2.97	—	2.43
Estonia - Tallinn	—	-1.46	-1.42	-1.35	-3.28	-2.53	-2.91	—	7.18
Bulgaria - Sofia	-3.26	-6.18	-1.19	-3.90	0.61	0.62	-2.83	—	2.48
Bosnia and Herzegovina-Sarajevo	2.54	-4.08	-2.79	-2.81	-1.11	-1.94	-2.68	—	5.45
Belgium - Brussels	—	-1.29	-0.94	-1.31	-1.48	-0.94	-1.81	—	1.88
Germany - Berlin	-1.23	-2.43	-0.63	-1.20	-0.63	-0.87	-1.73	—	1.46
Russia - Moscow	—	—	-0.12	—	-0.51	-1.49	-1.58	—	9.72
France - Paris	0.87	-0.21	-2.24	-1.66	-0.43	-0.49	-1.55	—	3.85
Czech Republic - Prague	—	—	2.49	—	2.74	1.14	-0.31	—	3.18
Croatia - Zagreb	0.36	-1.14	0.67	-0.33	-8.66	-9.80	-0.04	—	6.43
Latvia-Riga	-0.13	-1.66	5.61	0.04	0.31	-0.56	0.15	—	43.19
Romania - Bucharest	—	0.65	—	1.68	-2.42	—	0.16	—	2.48
Macedonia-Skopje	—	-2.17	-0.88	-1.31	-0.93	-1.21	0.22	—	3.04
Luxembourg - Luxembourg	—	—	1.33	—	2.31	0.60	0.38	—	1.97

Table S1. *Cont.*

City	As (I_{geo})	Cr (I_{geo})	Cu (I_{geo})	Ni (I_{geo})	Pb (I_{geo})	Zn (I_{geo})	Cd (I_{geo})	Hg (I_{geo})	P_N
Norway - Oslo	0.28	-1.39	0.53	-0.74	-0.27	0.02	0.87	—	2.25
Serbia-Belgrade	—	0.10	1.61	0.54	-0.38	0.86	0.90	—	1.78
Portugal - Lisbon	—	—	0.92	—	2.11	1.39	1.21	—	1.93
Slovakia-Bratislava	0.78	-1.25	3.49	0.44	1.23	1.05	1.98	—	9.94
Slovenia-Ljubljana	—	1.38	-0.16	-0.52	2.68	-1.75	—	—	1.71
Spain - Madrid	—	—	-0.77	-2.58	1.12	-0.99	—	—	4.88
Greece - Athens	-0.85	-2.25	1.74	-4.32	-0.86	-0.76	—	—	27.18
Hungary - Budapest	—	-1.04	-0.52	-0.58	-0.58	-0.14	—	—	17.73
Italy-Rome	—	-0.67	0.02	0.99	0.02	0.08	—	—	14.76
UK - London	-0.91	-0.78	-0.30	-1.32	—	—	—	—	18.80
Fiji-Suva	—	0.06	1.25	0.10	-0.25	1.23	-0.39	—	136.58
Australia - Melbourne	—	-1.63	-1.37	-1.07	2.18	2.48	—	—	9.03
Australia - Sydney	—	-0.50	-0.08	-4.46	2.75	0.72	2.89	—	6.01
Ethiopia - Addis Ababa	—	4.78	—	2.39	-0.44	-0.53	—	—	1.21
Egypt - Cairo	—	-2.39	-2.67	-1.51	-2.48	-2.60	0.91	—	10.52
Angola-Luanda	-1.24	-0.57	-0.58	—	-0.59	-0.58	-0.65	—	5.53
Botswana - Gaborone	—	0.70	-0.02	0.37	0.22	—	—	—	143.16
Burkina Faso - Ouagadougou	—	-0.21	0.06	-0.46	2.57	—	—	—	1.34
Eritrea-Asmara	—	0.31	2.61	0.55	1.56	—	—	—	0.24
Congo-Kinsassa	—	-5.88	-7.89	—	-7.94	—	—	—	1.28
Ghana-Accra	—	-7.46	-3.88	-4.66	-4.30	—	—	—	7.18
Cameroon - Yaoundé	—	0.28	—	-1.46	0.23	—	—	—	9.27
Kenya - Nairobi	-11.34	-7.20	-6.78	-5.65	—	-5.59	-8.19	-6.68	0.88
Libya - Misrata	—	-0.99	0.25	-0.12	-1.96	0.10	—	—	2.98
Mozambique - Maputo	—	-0.28	2.11	-0.78	1.37	4.35	—	—	0.82
Namibia-Windhoek	—	—	0.55	2.25	2.74	1.95	—	—	2.96
Tanzania - Dodoma	0.10	-1.47	-2.19	-1.97	-0.42	-1.53	-0.58	—	0.89
Tunisia - Tunisia	—	-7.27	-7.49	-8.42	-9.55	-8.82	-6.09	—	0.16
Zambia - Lusaka	—	—	-1.76	—	-2.79	-1.91	-4.91	—	1.02

I_{geo} is the Geo-accumulation index; P_N is the Nemerow composite pollution index. The classification of pollution degrees is based on Müller (1969) and Nemerow (1974). — indicates no data available.

Table S3. Non-Carcinogenic Health Risk Indices (*HQ*/*HI*) for Children and Adults Based on US EPA Mode.

Continent	Hazard Quotient of Children	As	Cr	Cu	Ni	Pb	Zn	Cd	Hg	*HI*
Asia	HQ_{ing}	$7.73×10^{-1}$	$4.02×10^{-1}$	$1.23×10^{-1}$	$1.26×10^{-2}$	$1.16×10^{-2}$	$3.55×10^{-3}$	$2.04×10^{-2}$	$8.82×10^{-2}$	—
	HQ_{inh}	$1.04×10^{-12}$	$7.75×10^{-12}$	$5.05×10^{-13}$	$6.76×10^{-7}$	$6.36×10^{-6}$	$1.96×10^{-7}$	$1.13×10^{-4}$	$1.70×10^{-5}$	—
	HQ_{der}	$3.17×10^{-4}$	$3.75×10^{-2}$	$1.72×10^{-4}$	$2.62×10^{-4}$	$4.33×10^{-3}$	$9.93×10^{-5}$	$1.14×10^{-2}$	$7.06×10^{-3}$	—
	ΣHQ	$7.73×10^{-1}$	$4.39×10^{-1}$	$1.23×10^{-1}$	$1.29×10^{-2}$	$1.59×10^{-2}$	$3.65×10^{-3}$	$3.19×10^{-2}$	$9.53×10^{-2}$	**$1.50×10^{0}$**
America	HQ_{ing}	$3.27×10^{-1}$	$3.03×10^{-1}$	$1.28×10^{-1}$	$1.27×10^{-2}$	$1.79×10^{-2}$	$2.94×10^{-3}$	$5.00×10^{-3}$	$3.30×10^{-3}$	—
	HQ_{inh}	$4.40×10^{-13}$	$5.84×10^{-12}$	$5.26×10^{-13}$	$6.78×10^{-13}$	$9.81×10^{-12}$	$1.62×10^{-13}$	$2.76×10^{-11}$	$6.36×10^{-13}$	—
	HQ_{der}	$1.34×10^{-4}$	$2.83×10^{-2}$	$1.79×10^{-4}$	$2.63×10^{-4}$	$6.68×10^{-3}$	$8.22×10^{-5}$	$2.80×10^{-3}$	$2.64×10^{-4}$	—
	ΣHQ	$3.27×10^{-1}$	$3.31×10^{-1}$	$1.28×10^{-1}$	$1.29×10^{-2}$	$2.46×10^{-2}$	$3.02×10^{-3}$	$7.79×10^{-3}$	$3.56×10^{-3}$	$8.38×10^{-1}$
Africa	HQ_{ing}	$1.60×10^{-1}$	$3.40×10^{-1}$	$1.66×10^{-1}$	$1.59×10^{-2}$	$8.83×10^{-2}$	$5.59×10^{-3}$	$6.79×10^{-3}$	$7.62×10^{-2}$	—
	HQ_{inh}	$2.15×10^{-13}$	$6.56×10^{-12}$	$6.82×10^{-13}$	$8.52×10^{-13}$	$4.84×10^{-11}$	$3.08×10^{-13}$	$3.74×10^{-11}$	$1.47×10^{-11}$	—
	HQ_{der}	$6.56×10^{-5}$	$3.17×10^{-2}$	$2.32×10^{-4}$	$3.30×10^{-4}$	$3.30×10^{-2}$	$1.57×10^{-4}$	$3.80×10^{-3}$	$6.10×10^{-3}$	—
	ΣHQ	$1.60×10^{-1}$	$3.72×10^{-1}$	$1.66×10^{-1}$	$1.62×10^{-2}$	$1.21×10^{-1}$	$5.75×10^{-3}$	$1.06×10^{-2}$	$8.23×10^{-2}$	$9.34×10^{-1}$
Europe	HQ_{ing}	$8.25×10^{-2}$	$3.53×10^{-1}$	$1.64×10^{-1}$	$1.23×10^{-2}$	$1.48×10^{-2}$	$3.36×10^{-3}$	$2.12×10^{-2}$	$6.05×10^{-3}$	—
	HQ_{inh}	$1.11×10^{-13}$	$6.81×10^{-12}$	$6.73×10^{-13}$	$6.57×10^{-13}$	$8.13×10^{-12}$	$1.85×10^{-13}$	$1.17×10^{-10}$	$1.17×10^{-12}$	—
	HQ_{der}	$3.38×10^{-5}$	$3.29×10^{-2}$	$2.29×10^{-4}$	$2.55×10^{-4}$	$5.53×10^{-3}$	$9.41×10^{-5}$	$1.19×10^{-2}$	$4.84×10^{-4}$	—
	ΣHQ	$8.25×10^{-2}$	$3.86×10^{-1}$	$1.64×10^{-1}$	$1.25×10^{-2}$	$2.04×10^{-2}$	$3.45×10^{-3}$	$3.31×10^{-2}$	$6.53×10^{-3}$	$7.08×10^{-1}$
Oceania	HQ_{ing}	—	$1.77×10^{-1}$	$2.71×10^{-1}$	$5.77×10^{-3}$	$4.63×10^{-2}$	$7.37×10^{-3}$	$1.54×10^{-2}$	—	—
	HQ_{inh}	—	$3.41×10^{-12}$	$1.11×10^{-12}$	$3.09×10^{-13}$	$2.54×10^{-11}$	$4.06×10^{-13}$	$8.48×10^{-11}$	—	—
	HQ_{der}	—	$1.65×10^{-2}$	$3.79×10^{-4}$	$1.20×10^{-4}$	$1.73×10^{-2}$	$2.06×10^{-4}$	$8.61×10^{-3}$	—	—
	ΣHQ	—	$1.93×10^{-1}$	$2.71×10^{-1}$	$5.89×10^{-3}$	$6.35×10^{-2}$	$7.58×10^{-3}$	$2.40×10^{-2}$	—	$5.65×10^{-1}$

Table S3. *Cont.*

Continent	Hazard Quotient of Adult	As	Cr	Cu	Ni	Pb	Zn	Cd	Hg	HI
Asia	HQ_{ing}	$4.62×10^{-2}$	$2.40×10^{-2}$	$7.34×10^{-3}$	$7.55×10^{-4}$	$6.93×10^{-4}$	$2.12×10^{-4}$	$1.22×10^{-3}$	$5.27×10^{-3}$	—
	HQ_{inh}	$6.01×10^{-13}$	$4.48×10^{-12}$	$2.92×10^{-13}$	$3.91×10^{-13}$	$3.68×10^{-12}$	$1.13×10^{-13}$	$6.50×10^{-11}$	$9.84×10^{-12}$	—
	HQ_{der}	$6.75×10^{-4}$	$7.99×10^{-2}$	$3.66×10^{-4}$	$5.58×10^{-4}$	$9.22×10^{-3}$	$2.12×10^{-4}$	$2.43×10^{-2}$	$1.50×10^{-2}$	—
	$ΣHQ$	$4.69×10^{-2}$	$1.04×10^{-1}$	$7.70×10^{-3}$	$1.31×10^{-3}$	$9.92×10^{-3}$	$4.24×10^{-4}$	$2.55×10^{-2}$	$2.03×10^{-2}$	$2.16×10^{-1}$
America	HQ_{ing}	$1.96×10^{-2}$	$1.81×10^{-2}$	$7.64×10^{-3}$	$7.57×10^{-4}$	$1.07×10^{-3}$	$1.76×10^{-4}$	$2.99×10^{-4}$	$1.97×10^{-4}$	—
	HQ_{inh}	$2.54×10^{-13}$	$3.37×10^{-12}$	$3.04×10^{-13}$	$3.92×10^{-13}$	$5.67×10^{-12}$	$9.36×10^{-14}$	$1.59×10^{-11}$	$3.68×10^{-13}$	—
	HQ_{der}	$2.86×10^{-4}$	$6.02×10^{-2}$	$3.81×10^{-4}$	$5.59×10^{-4}$	$1.42×10^{-2}$	$1.75×10^{-4}$	$5.96×10^{-3}$	$5.62×10^{-4}$	—
	$ΣHQ$	$1.98×10^{-2}$	$7.83×10^{-2}$	$8.02×10^{-3}$	$1.32×10^{-3}$	$1.53×10^{-2}$	$3.51×10^{-4}$	$6.26×10^{-3}$	$7.59×10^{-4}$	$1.30×10^{-1}$
Africa	HQ_{ing}	$9.57×10^{-3}$	$2.03×10^{-2}$	$9.90×10^{-3}$	$9.51×10^{-4}$	$5.28×10^{-3}$	$3.34×10^{-4}$	$4.06×10^{-4}$	$4.56×10^{-3}$	—
	HQ_{inh}	$1.24×10^{-13}$	$3.79×10^{-12}$	$3.94×10^{-13}$	$4.92×10^{-13}$	$2.80×10^{-11}$	$1.78×10^{-13}$	$2.16×10^{-11}$	$8.50×10^{-12}$	—
	HQ_{der}	$1.40×10^{-4}$	$6.76×10^{-2}$	$4.94×10^{-4}$	$7.03×10^{-4}$	$7.02×10^{-2}$	$3.33×10^{-4}$	$8.10×10^{-3}$	$1.30×10^{-2}$	—
	$ΣHQ$	$9.70×10^{-3}$	$8.79×10^{-2}$	$1.04×10^{-2}$	$1.65×10^{-3}$	$7.55×10^{-2}$	$6.68×10^{-4}$	$8.50×10^{-3}$	$1.75×10^{-2}$	$2.12×10^{-1}$
Europe	HQ_{ing}	$4.93×10^{-3}$	$2.11×10^{-2}$	$9.78×10^{-3}$	$7.34×10^{-4}$	$8.86×10^{-4}$	$2.01×10^{-4}$	$1.27×10^{-3}$	$3.61×10^{-4}$	—
	HQ_{inh}	$6.41×10^{-14}$	$3.93×10^{-12}$	$3.89×10^{-13}$	$3.80×10^{-13}$	$4.70×10^{-12}$	$1.07×10^{-13}$	$6.77×10^{-11}$	$6.74×10^{-13}$	—
	HQ_{der}	$7.19×10^{-5}$	$7.02×10^{-2}$	$4.88×10^{-4}$	$5.42×10^{-4}$	$1.18×10^{-2}$	$2.00×10^{-4}$	$2.53×10^{-2}$	$1.03×10^{-3}$	—
	$ΣHQ$	$5.00×10^{-3}$	$9.13×10^{-2}$	$1.03×10^{-2}$	$1.28×10^{-3}$	$1.27×10^{-2}$	$4.01×10^{-4}$	$2.66×10^{-2}$	$1.39×10^{-3}$	$1.49×10^{-1}$
Oceania	HQ_{ing}	—	$1.06×10^{-2}$	$1.62×10^{-2}$	$3.45×10^{-4}$	$2.76×10^{-3}$	$4.41×10^{-4}$	$9.19×10^{-4}$	—	—
	HQ_{inh}	—	$1.97×10^{-12}$	$6.43×10^{-13}$	$1.78×10^{-13}$	$1.47×10^{-11}$	$2.35×10^{-13}$	$4.90×10^{-11}$	—	—
	HQ_{der}	—	$3.52×10^{-2}$	$8.06×10^{-4}$	$2.55×10^{-4}$	$3.68×10^{-2}$	$4.39×10^{-4}$	$1.83×10^{-2}$	—	—
	$ΣHQ$	—	$4.57×10^{-2}$	$1.70×10^{-2}$	$6.00×10^{-4}$	$3.95×10^{-2}$	$8.80×10^{-4}$	$1.93×10^{-2}$	—	$1.23×10^{-1}$

HQ_{ing}, HQ_{inh} and HQ_{der} represent the hazard quotients for ingestion, inhalation, and dermal contact pathways, respectively. *HI* is the total hazard index. Values in bold exceed the safe threshold ($HI > 1$). — indicates no available data.

Table S4. Carcinogenic Risk (*CR*) of PTEs for Children and Adults Based on US EPA Model.

Continent	Carcinogenic Risk in children	As	Cr	Ni	Cd	ΣCR
Asia	CR_{ing}	3.48×10^{-4}	2.01×10^{-4}	—	1.24×10^{-7}	—
	CR_{inh}	1.93×10^{-13}	9.31×10^{-13}	1.17×10^{-8}	7.09×10^{-9}	—
	CR_{der}	5.85×10^{-6}	4.50×10^{-5}	6.01×10^{-5}	6.97×10^{-7}	—
	ΣCR_i	3.54×10^{-4}	2.46×10^{-4}	6.01×10^{-5}	8.28×10^{-7}	3.13×10^{-4}
America	CR_{ing}	1.47×10^{-4}	1.51×10^{-4}	—	3.05×10^{-8}	—
	CR_{inh}	8.17×10^{-14}	7.01×10^{-13}	1.17×10^{-14}	1.74×10^{-15}	—
	CR_{der}	2.47×10^{-6}	3.39×10^{-5}	6.03×10^{-5}	1.71×10^{-7}	—
	ΣCR_i	1.50×10^{-4}	1.85×10^{-4}	6.03×10^{-5}	2.01×10^{-7}	2.49×10^{-4}
Africa	CR_{ing}	7.20×10^{-5}	1.70×10^{-4}	—	4.14×10^{-8}	—
	CR_{inh}	4.00×10^{-14}	7.88×10^{-13}	1.47×10^{-14}	2.36×10^{-15}	—
	CR_{der}	1.21×10^{-6}	3.81×10^{-5}	7.57×10^{-5}	2.32×10^{-7}	—
	ΣCR_i	7.32×10^{-5}	2.08×10^{-4}	7.57×10^{-5}	2.73×10^{-7}	2.85×10^{-4}
Europe	CR_{ing}	3.71×10^{-5}	1.77×10^{-4}	—	1.30×10^{-7}	—
	CR_{inh}	2.06×10^{-14}	8.18×10^{-13}	1.14×10^{-14}	7.38×10^{-15}	—
	CR_{der}	6.23×10^{-7}	3.95×10^{-5}	5.84×10^{-5}	7.25×10^{-7}	—
	ΣCR_i	3.77×10^{-5}	2.16×10^{-4}	5.84×10^{-5}	8.55×10^{-7}	2.76×10^{-4}
Oceania	CR_{ing}	—	8.85×10^{-5}	—	9.38×10^{-8}	—
	CR_{inh}	—	4.10×10^{-13}	5.35×10^{-15}	5.34×10^{-15}	—
	CR_{der}	—	1.98×10^{-5}	2.75×10^{-5}	5.25×10^{-7}	—
	ΣCR_i	—	1.08×10^{-4}	2.75×10^{-5}	6.19×10^{-7}	1.36×10^{-4}

Table S4. *Cont.*

Continent	Carcinogenic Risk in Adult	As	Cr	Ni	Cd	*ΣCR*
Asia	CR_{ing}	2.08×10^{-5}	1.20×10^{-5}	—	7.44×10^{-9}	—
	CR_{inh}	1.12×10^{-13}	5.38×10^{-13}	6.76×10^{-15}	4.10×10^{-15}	—
	CR_{der}	1.24×10^{-5}	9.59×10^{-5}	1.28×10^{-4}	1.48×10^{-6}	—
	ΣCR_i	3.32×10^{-5}	1.08×10^{-4}	1.28×10^{-4}	1.49×10^{-6}	2.50×10^{-4}
America	CR_{ing}	8.81×10^{-6}	9.05×10^{-6}	—	1.82×10^{-9}	—
	CR_{inh}	4.72×10^{-14}	4.05×10^{-13}	6.78×10^{-15}	1.00×10^{-15}	—
	CR_{der}	5.27×10^{-6}	7.22×10^{-5}	1.28×10^{-4}	3.63×10^{-7}	—
	ΣCR_i	1.41×10^{-5}	8.12×10^{-5}	1.28×10^{-4}	3.65×10^{-7}	2.15×10^{-4}
Africa	CR_{ing}	4.30×10^{-6}	1.02×10^{-5}	—	2.48×10^{-9}	—
	CR_{inh}	2.31×10^{-14}	4.55×10^{-13}	8.52×10^{-15}	1.36×10^{-15}	—
	CR_{der}	2.58×10^{-6}	8.11×10^{-5}	1.61×10^{-4}	4.94×10^{-7}	—
	ΣCR_i	6.88×10^{-6}	9.13×10^{-5}	1.61×10^{-4}	4.96×10^{-7}	2.56×10^{-4}
Europe	CR_{ing}	2.22×10^{-6}	1.06×10^{-5}	—	7.74×10^{-9}	—
	CR_{inh}	1.19×10^{-14}	4.72×10^{-13}	6.57×10^{-15}	4.26×10^{-15}	—
	CR_{der}	1.33×10^{-6}	8.42×10^{-5}	1.24×10^{-4}	1.54×10^{-6}	—
	ΣCR_i	3.54×10^{-6}	9.47×10^{-5}	1.24×10^{-4}	1.55×10^{-6}	2.22×10^{-4}
Oceania	CR_{ing}	—	5.29×10^{-6}	—	5.61×10^{-9}	—
	CR_{inh}	—	2.37×10^{-13}	3.09×10^{-15}	3.09×10^{-15}	—
	CR_{der}	—	4.22×10^{-5}	5.85×10^{-5}	1.12×10^{-6}	—
	ΣCR_i	—	4.75×10^{-5}	5.85×10^{-5}	1.12×10^{-6}	1.07×10^{-4}

CR_{ing}, CR_{inh} and CR_{der} represent the carcinogenic risks via ingestion, inhalation, and dermal contact pathways, respectively. ΣCR_i denotes the total carcinogenic risk (*TCR*). Values in bold exceed the US EPA's maximum acceptable threshold (1×10^{-4}). — indicates no data available.

Table S5. Temporal Variations of PTEs Concentrations in Selected Urban Soils (mg·kg^{-1}).

City	Time	As	Cr	Cu	Ni	Pb	Zn	Cd	Hg	Reference
China-Beijing	2007	7.67	25.48	31.43	15.85	65.25	177.74	—	8.28	[120]
	2010	—	33.96	30.762	22.1	33.724	52.006	0.638	—	[121]
	2019	7.28	65.96	22.7	30.55	32.27	85.31	0.15	0.14	[70]
China-Zhengzhou	2006	4.34	168.97	24.85	10.51	87.55	358.16	0.82	0.028	[20]
	2008	5.11	77.3	59.11	—	28.5	91.67	0.067	0.035	[129]
	2015	8.97	52.11	17.64	52.11	24.51	63.43	0.14	0.056	[128]
China-Chongqing	2004	13.22	79.45	46.57	37.82	50.77	128.62	0.212	0.483	[124]
	2008	5.86	93.63	30	30.89	29.39	89.22	0.29	0.102	[123]
	2013	5.7	41.8	31.3	—	28.9	85.2	—	0.127	[122]
China-Xi'an	2009	12.2	87.3	40.4	32.8	46.9	99.9	—	—	[127]
	2013	10.79	84.98	38.68	32.2	42.97	119.73	—	—	[125]
	2018	12.15	69.74	32.52	30.67	37.11	101.73	—	—	[126]
